# Supplementary material for: First evidence of a monodominant (Englerodendron, Amherstieae, Detarioideae, Leguminosae) tropical moist forest from the early Miocene (21.73 Ma) of Ethiopia
Source: PLoS One. 2023 Jan 11;18(1):e0279491. doi: 10.1371/journal.pone.0279491 (PMC9833558; doi:10.1371/journal.pone.0279491)
Supplement: S4 File — (PDF) [file pone.0279491.s004.pdf]

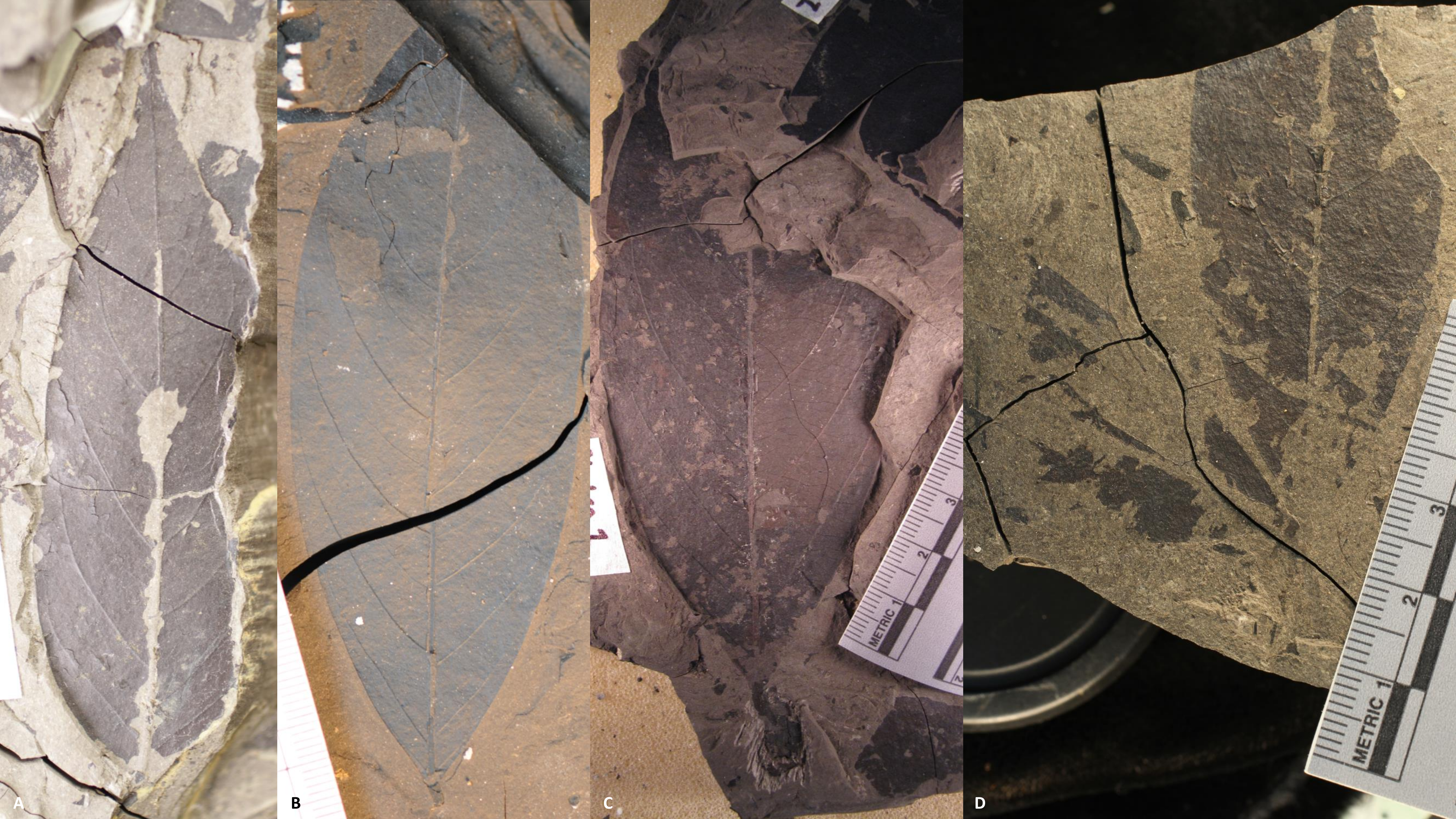
A
B
C
D
METRIC
METRIC

A. <u>Englerodendron mulugetanum</u> sp. nov. leaflet. MU7-35-A5A #23. Details of leaflet apex, base, and secondary venation.
B. <u>Englerodendron mulugetanum</u> sp. nov leaflet. MU13-28 #1. Details of leaflet shape, pulvinate petiole, secondary venation.
C. <u>Englerodendron mulugetanum</u> sp. nov leaflet. MU33-19 (1, piece 283) #29. Details of leaflet base shape, pulvinate petiole and attachment, and secondary and tertiary venation.
D. <u>Englerodendron mulugetanum</u> sp. nov leaflet. MU40-22. Terminal paripinnate leaflet pair with pulvinate petioles.
