# Supplementary material for: First evidence of a monodominant (Englerodendron, Amherstieae, Detarioideae, Leguminosae) tropical moist forest from the early Miocene (21.73 Ma) of Ethiopia
Source: PLoS One. 2023 Jan 11;18(1):e0279491. doi: 10.1371/journal.pone.0279491 (PMC9833558; doi:10.1371/journal.pone.0279491)
Supplement: S6 File — (PDF) [file pone.0279491.s006.pdf]

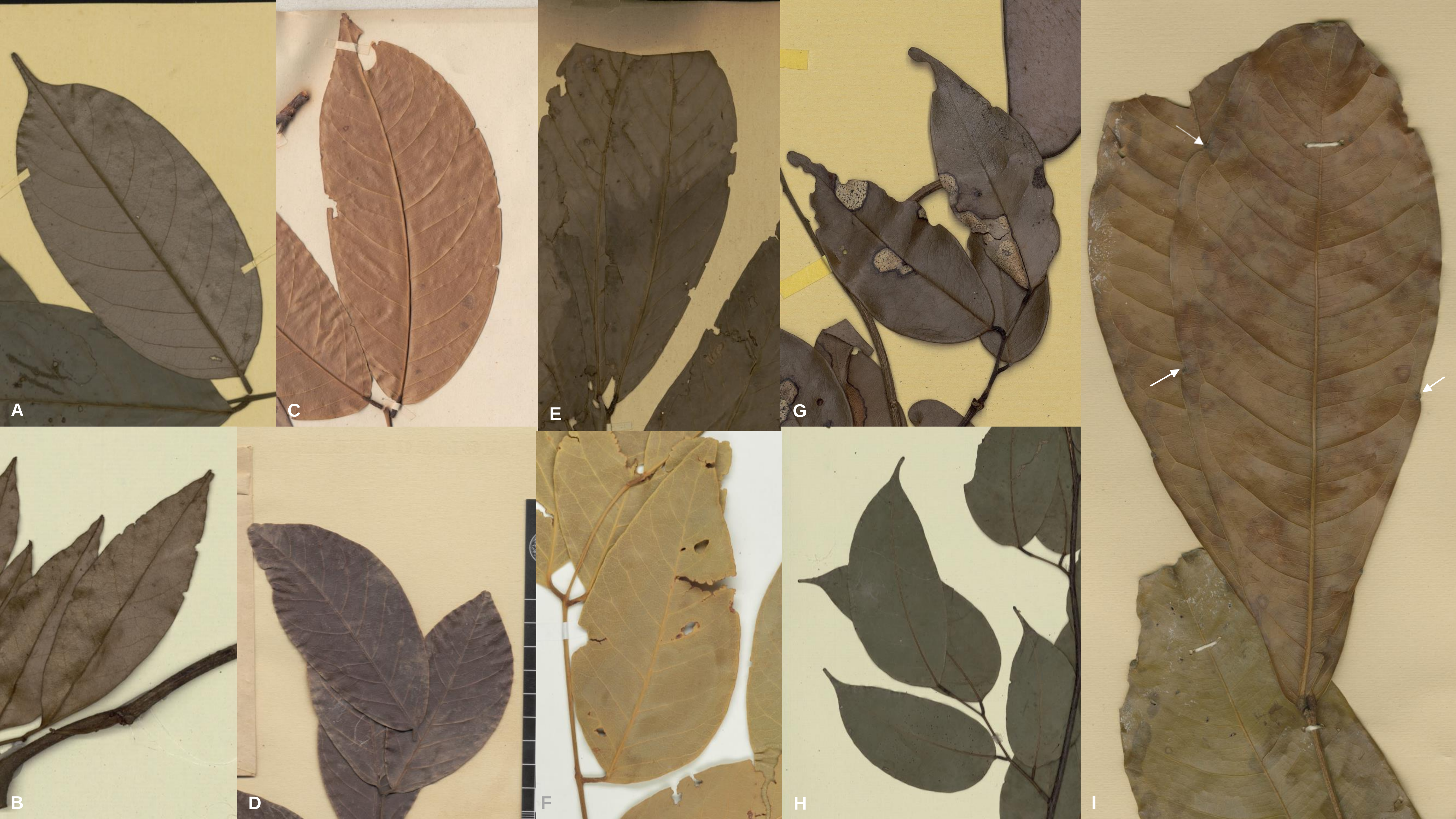
A
C
E
G
B
D
F
H
I

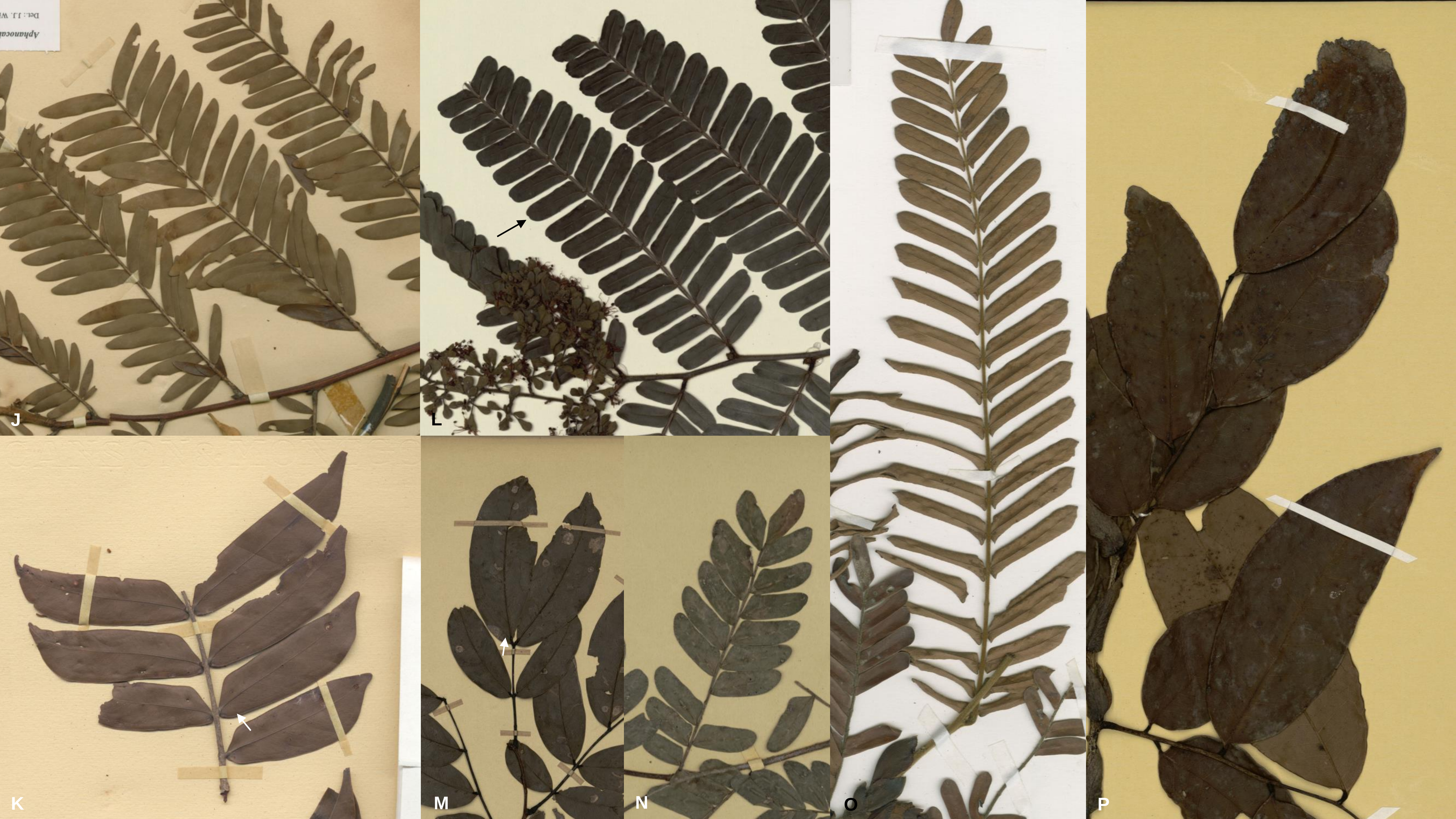
J
L
K
M
N
O
P

A. Anthonotha gilletii leaflet. MNHN-P-P03489813

B. Anthonotha noldeae leaflet. MNHN-P-P03503979

C. Berlinia auriculata leaflet. ISOTYPE. MNHN-P-P00190800

D. Isoberlinia scheffleri leaflets. MNHN-P-P00390071

E. Berlinia robynsianum leaflet. ISOTYPE. MNHN-P-P00486609

F. Isoberlinia doka leaflet. MNHN-P-P00925194

G. Librevillea klainei leaf and leaflets. Note alternate leaflet attachment and elongate petioles. MNHN-P-P02783512

H. Oddoniodendron romeroi leaf and leaflets. Note alternate leaflet attachment and elongate petioles. MNHN-P-P00299782

I. Gilbertiodendron robynsianum leaf and leaflets. Arrows denote marginal glands. Also note brochidodromous venation. ISOTYPE. MNHN-P-P00486609

J. Aphanocalyx heitzii leaf and leaflets. Note small, asymmetric leaflets. HOLOTYPE. MNHN-P-P00466357

K. Tetraberlinia moreliana leaf and leaflets. Arrow denotes 'fan' of basal veins. HOLOTYPE. MNHN-P-P00374732

L. Microberlinia bisulcata leaves and leaflets. Arrow denotes emarginated leaflet tip. MNHN-P-P00527607

M. Brachystegia laurentii leaf and leaflets. Arrow denotes prominent basal vein. MNHN-P-P00533242

N. Scorodophloeus zenkeri leaf and leaflets. MNHN-P-P03481343

O. Talbotiella korupensis leaf and leaflets. ISOTYPE. MNHN-P-P02142709

P. Crudia klainei leaves and leaflets. Note the twisted petiolules, alternate leaflet attachments, and brochidodromous venation. MNHN-P-P00187956.

CITATIONS

Muséum national d’Histoire naturelle, Paris (France)

Collection: Vascular plants (P)

Specimens:

P03489813
P03503979
P00190800
P00390071
P00486609
P00925194
P02783512
P00299782
P00486609
P00466357
P00374732
P00527607
P00533242
P03481343
P02142709
P00187956
