# Supplementary material for: First evidence of a monodominant (Englerodendron, Amherstieae, Detarioideae, Leguminosae) tropical moist forest from the early Miocene (21.73 Ma) of Ethiopia
Source: PLoS One. 2023 Jan 11;18(1):e0279491. doi: 10.1371/journal.pone.0279491 (PMC9833558; doi:10.1371/journal.pone.0279491)

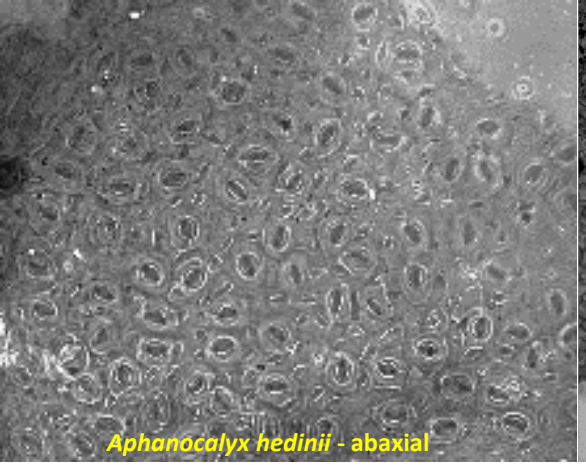

Aphanocalyx hedinii - abaxial

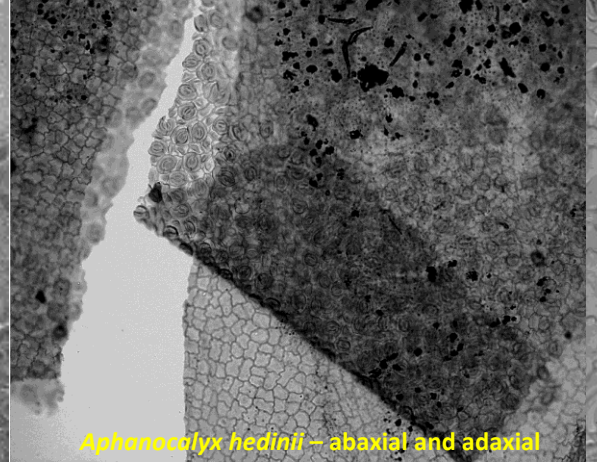

Aphanocalyx hedinii – abaxial and adaxial

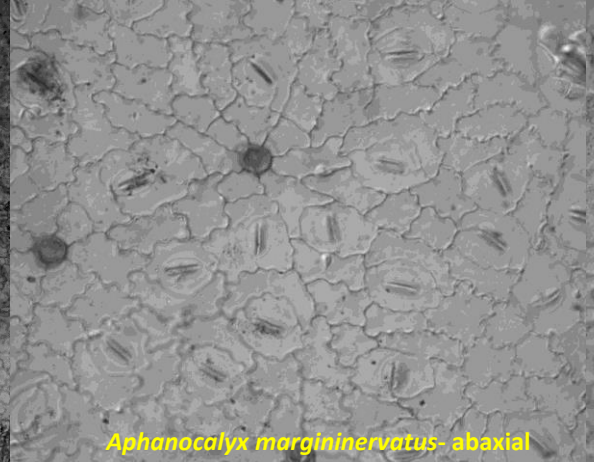

Aphanocalyx margininervatus- abaxial

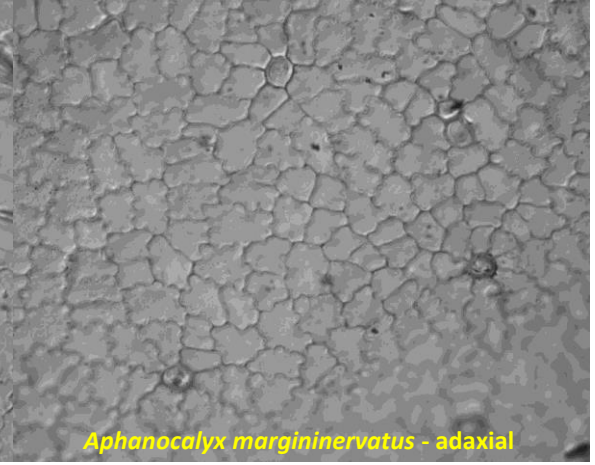

Aphanocalyx margininervatus - adaxial

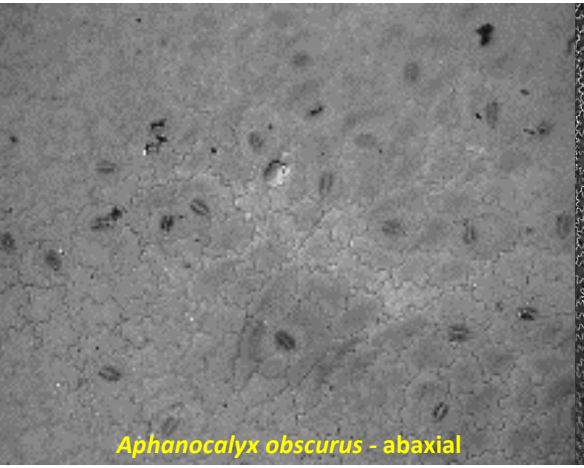

Aphanocalyx obscurus - abaxial

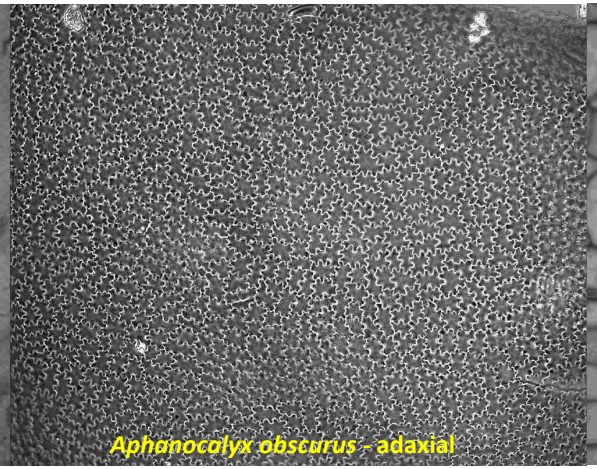

Aphanocalyx obscurus - adaxial

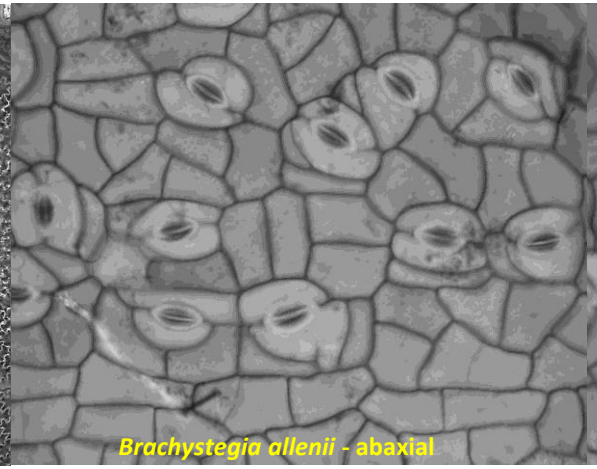

Brachystegia allenii - abaxial

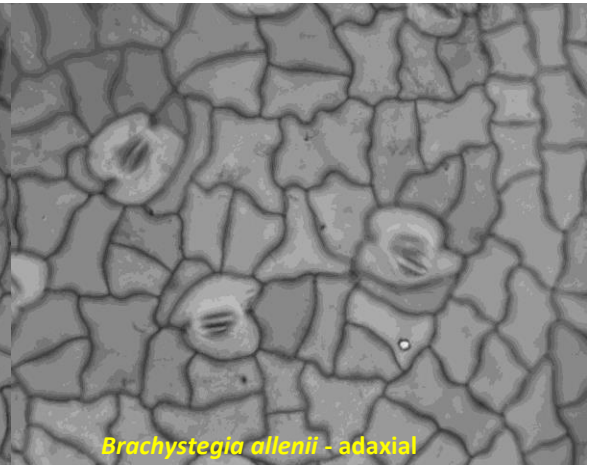

Brachystegia allenii - adaxial

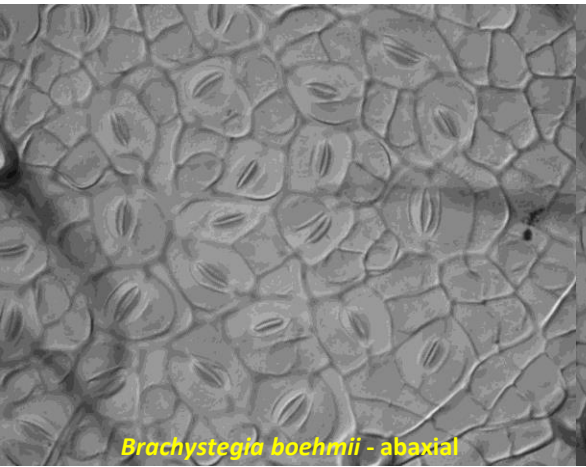

Brachystegia boehmii - abaxial

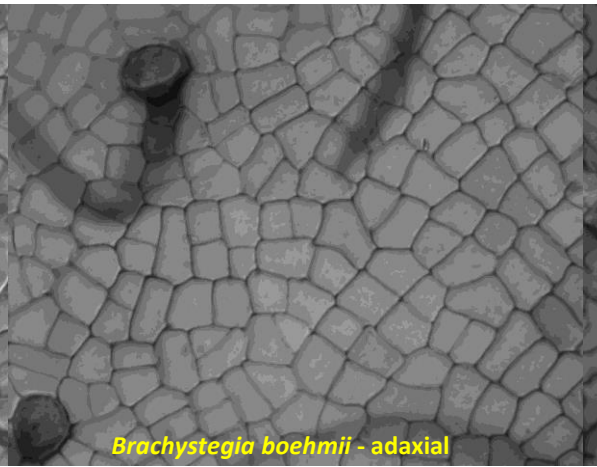

Brachystegia boehmii - adaxial

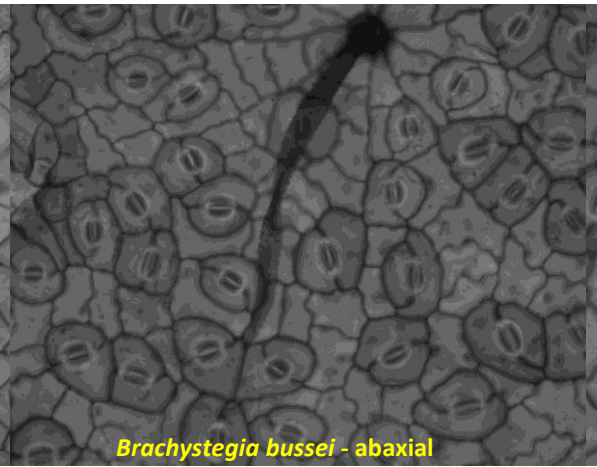

Brachystegia bussei - abaxial

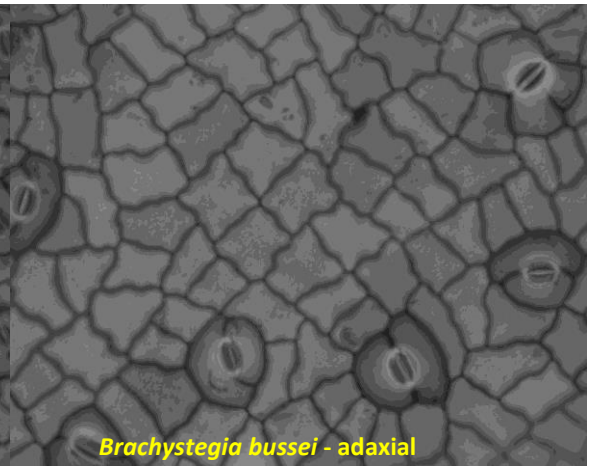

Brachystegia bussei - adaxial

Berlinia Clade – Subclade A

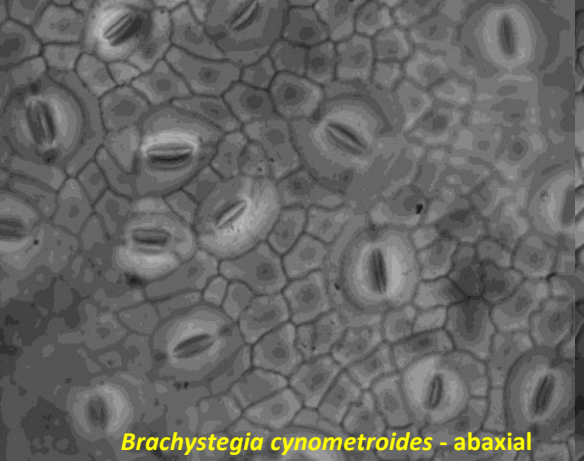
Brachystegia cynometroides - abaxial

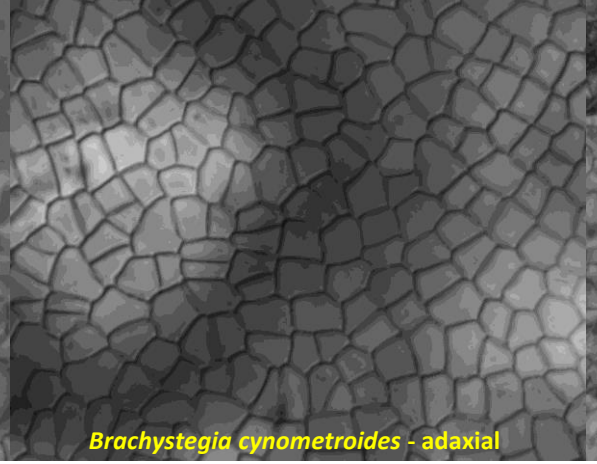
Brachystegia cynometroides - adaxial

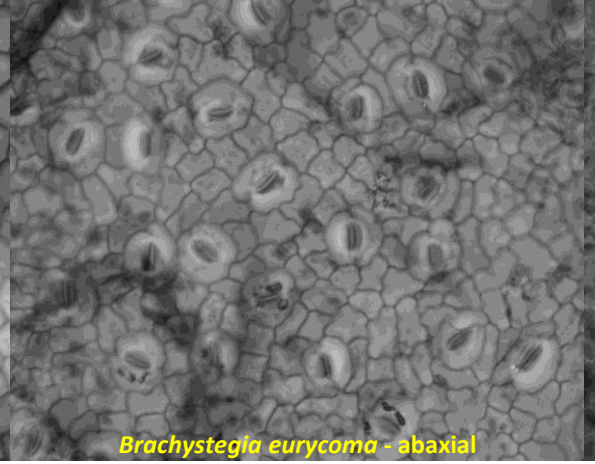
Brachystegia eurycoma - abaxial

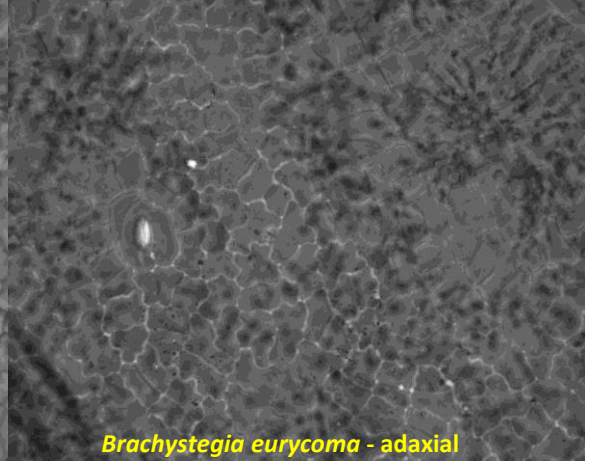
Brachystegia eurycoma - adaxial

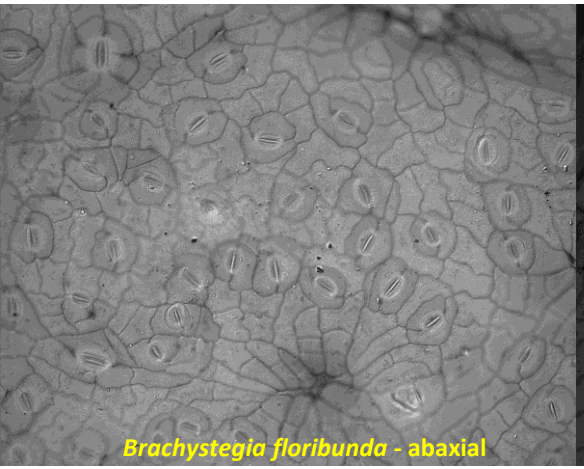
Brachystegia floribunda - abaxial

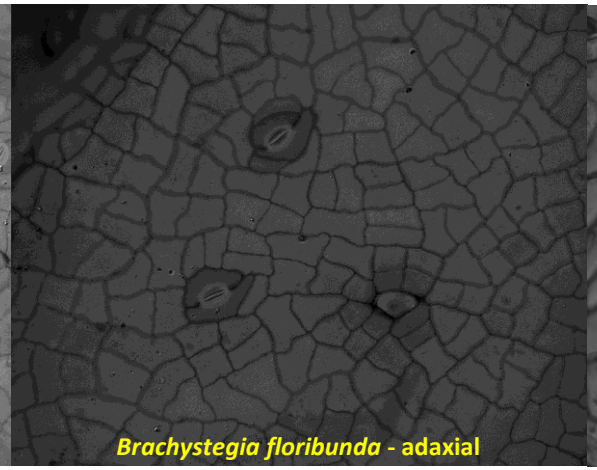
Brachystegia floribunda - adaxial

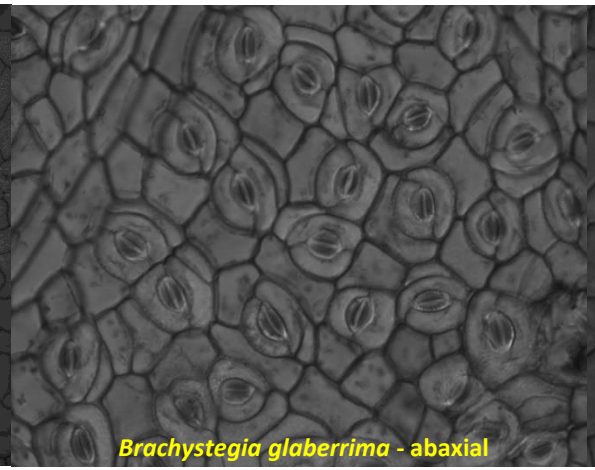
Brachystegia glaberrima - abaxial

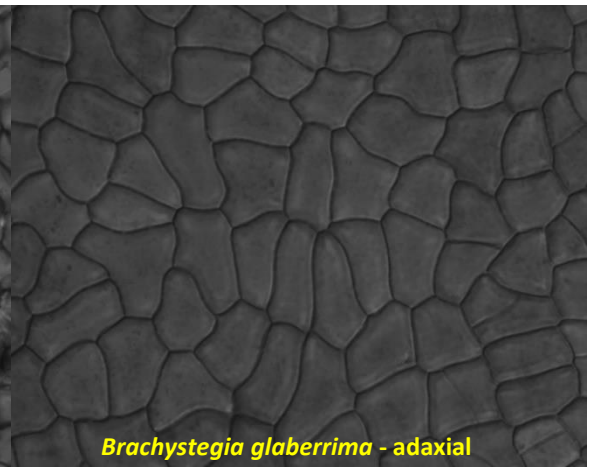
Brachystegia glaberrima - adaxial

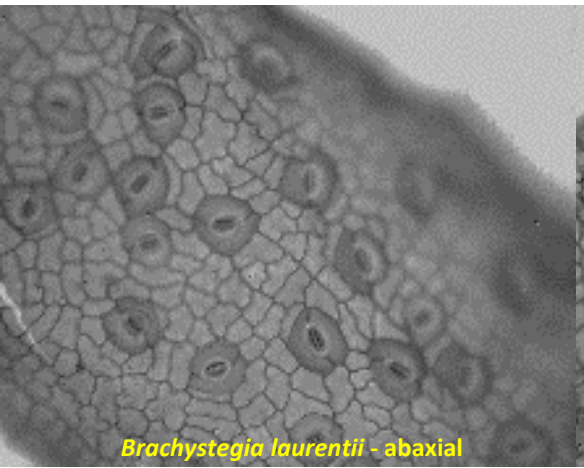
Brachystegia laurentii - abaxial

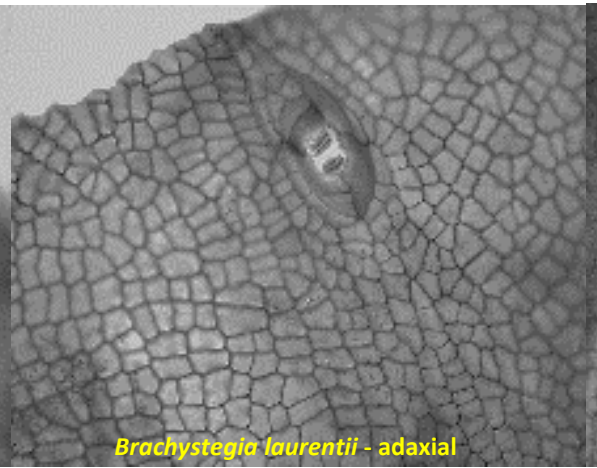
Brachystegia laurentii - adaxial

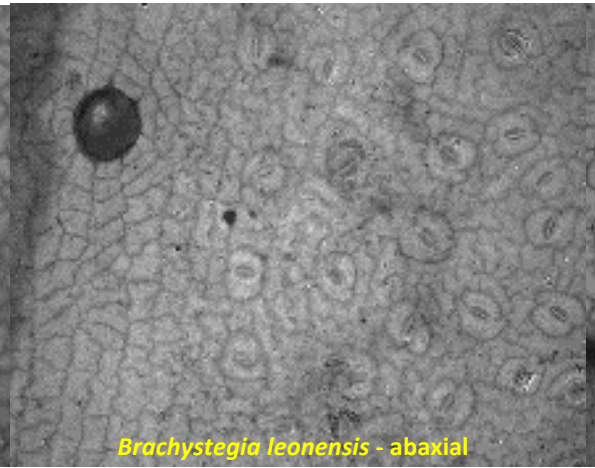
Brachystegia leonensis - abaxial

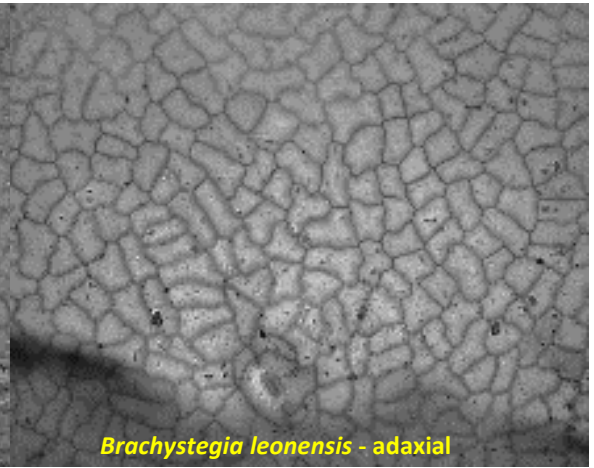
Brachystegia leonensis - adaxial

Berlinia Clade – Subclade A

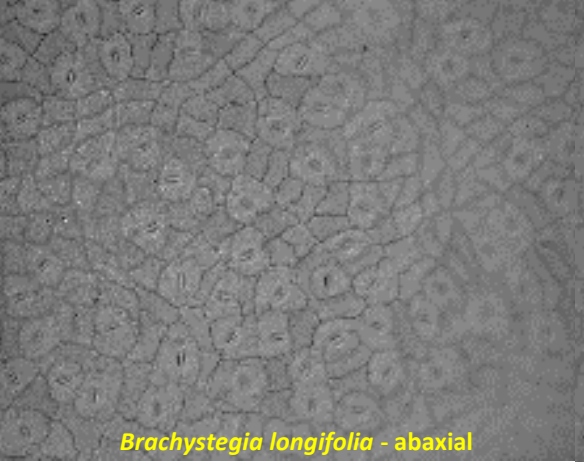
Brachystegia longifolia - abaxial

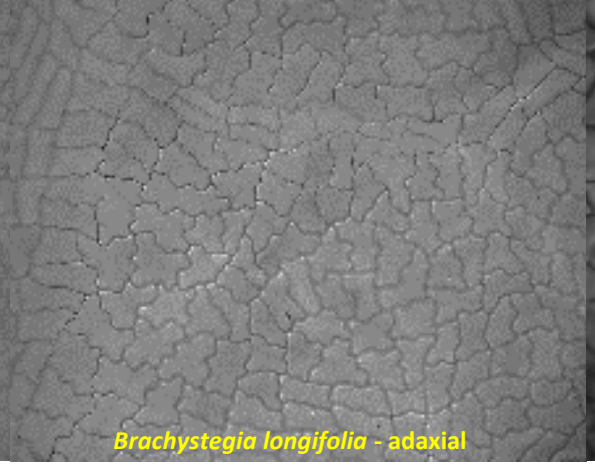
Brachystegia longifolia - adaxial

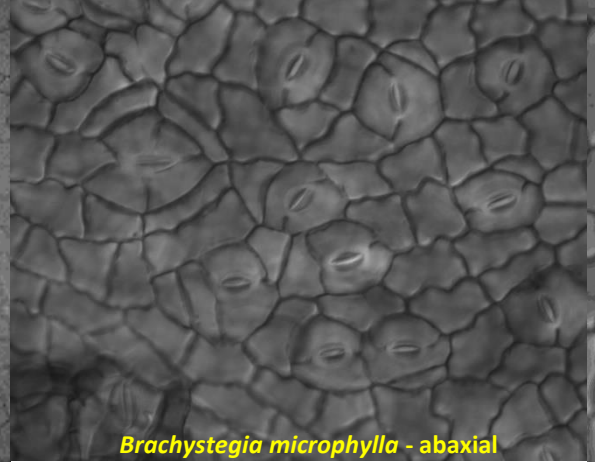
Brachystegia microphylla - abaxial

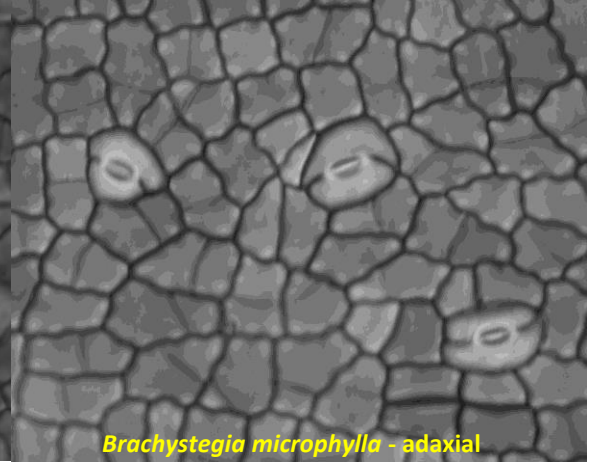
Brachystegia microphylla - adaxial

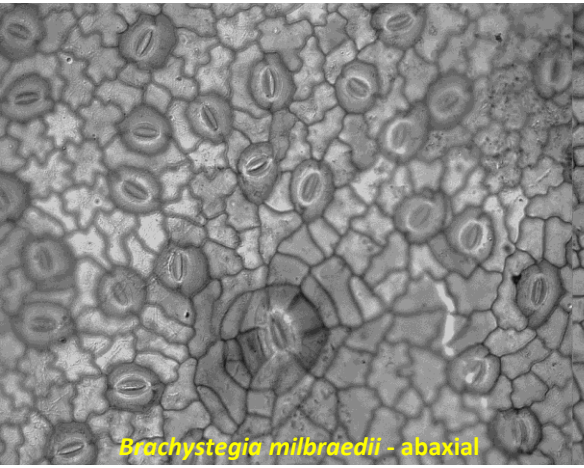
Brachystegia milbraedii - abaxial

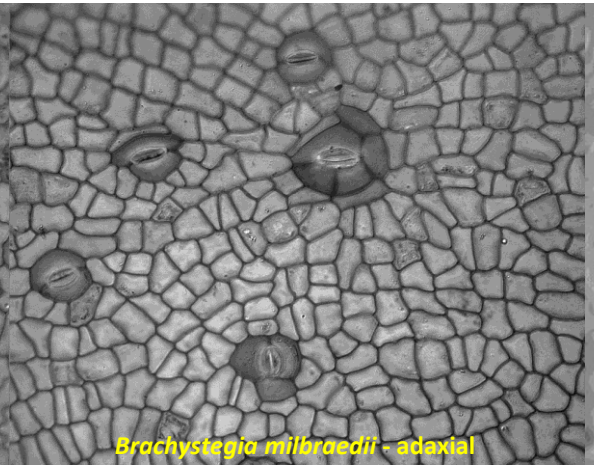
Brachystegia milbraedii - adaxial

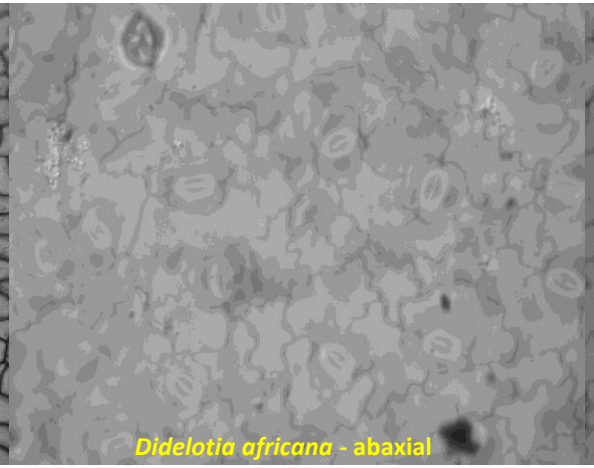
Didelotia africana - abaxial

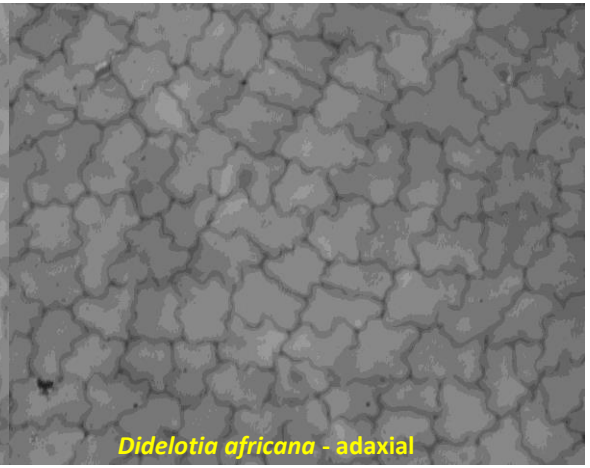
Didelotia africana - adaxial

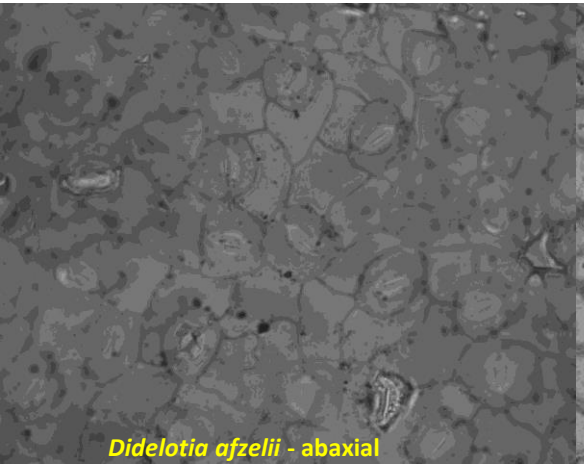
Didelotia afzelii - abaxial

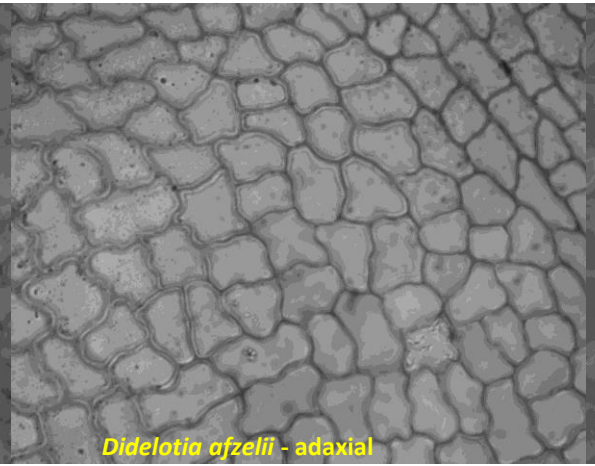
Didelotia afzelii - adaxial

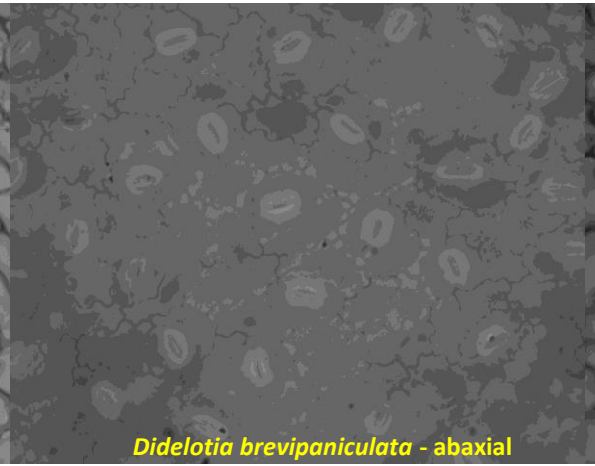
Didelotia brevipaniculata - abaxial

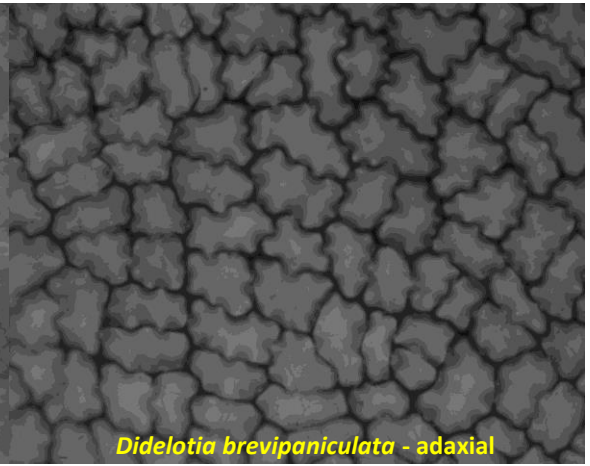
Didelotia brevipaniculata - adaxial

Berlinia Clade – Subclade A

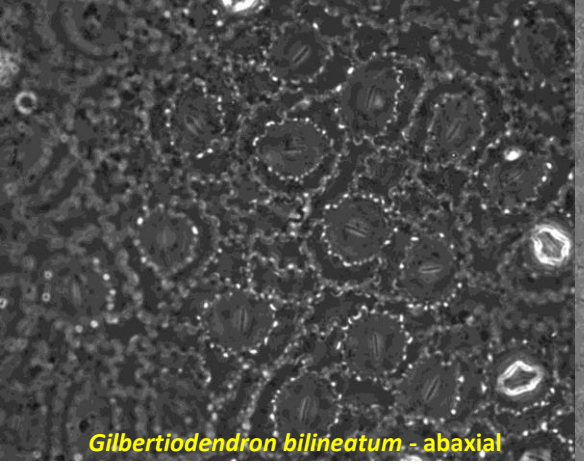

Gilbertiodendron bilineatum - abaxial

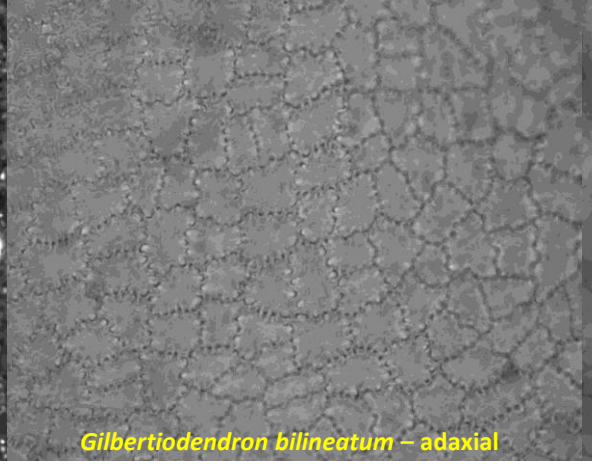

Gilbertiodendron bilineatum – adaxial

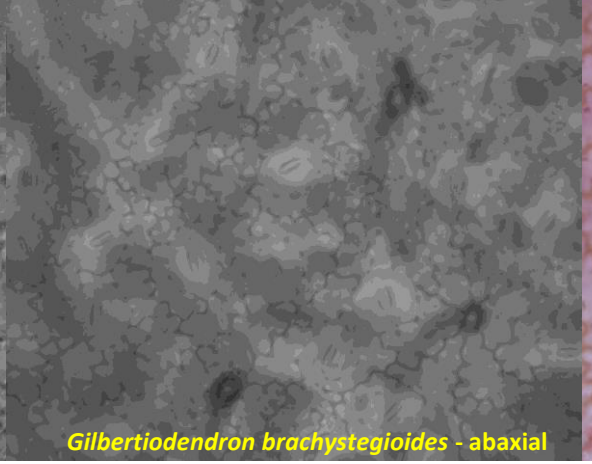

Gilbertiodendron brachystegioides - abaxial

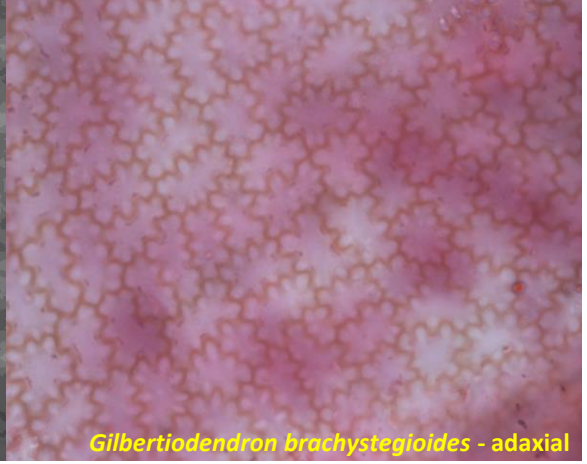

Gilbertiodendron brachystegioides - adaxial

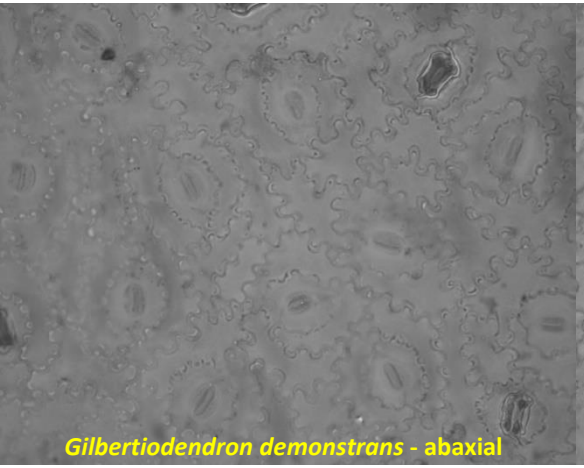

Gilbertiodendron demonstrans - abaxial

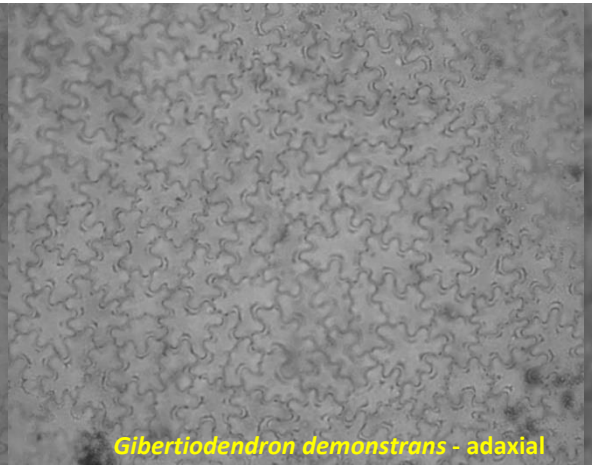

Gibertiodendron demonstrans - adaxial

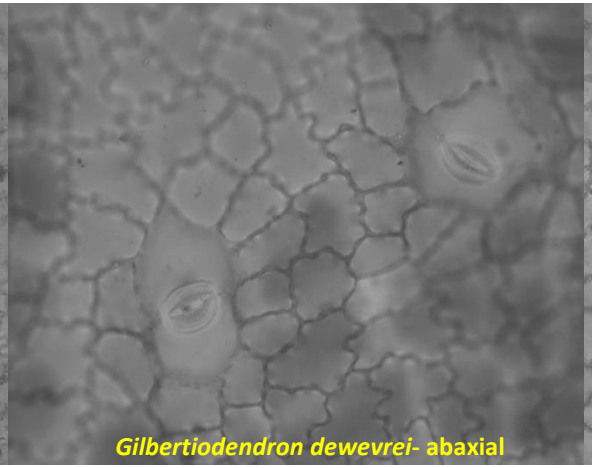

Gilbertiodendron dewevrei- abaxial

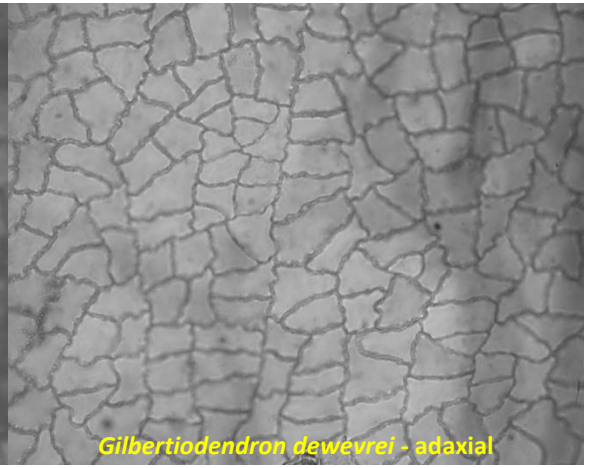

Gilbertiodendron dewevrei - adaxial

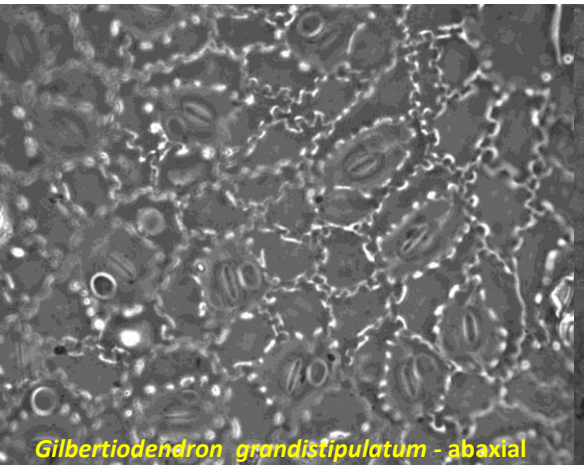

Gilbertiodendron grandistipulatum - abaxial

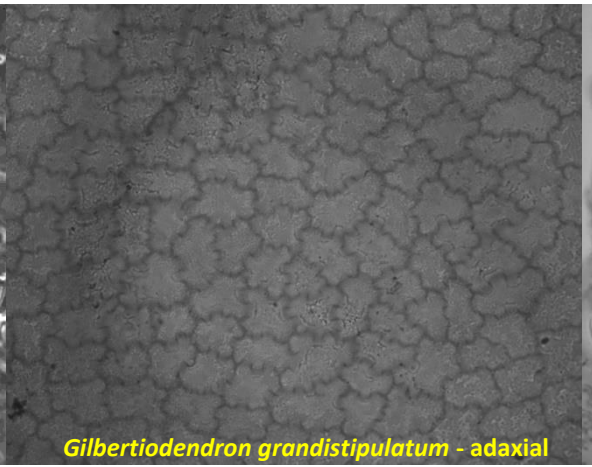

Gilbertiodendron grandistipulatum - adaxial

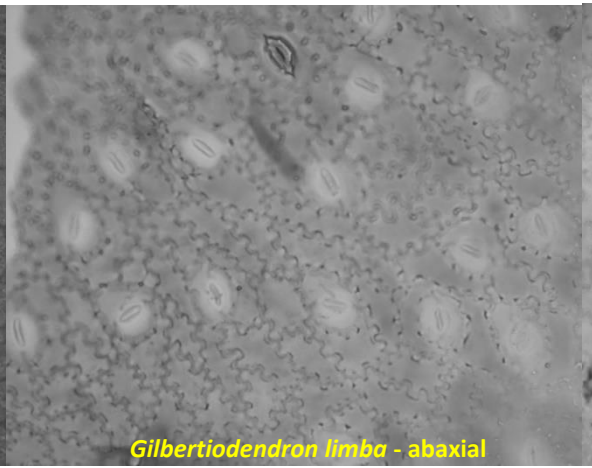

Gilbertiodendron limba - abaxial

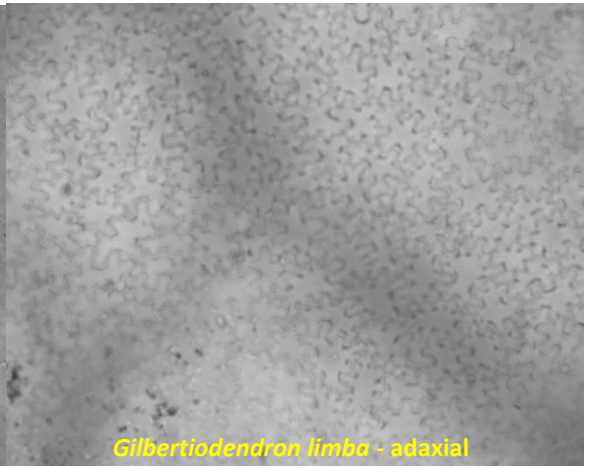

Gilbertiodendron limba - adaxial

Berlinia Clade – Subclade A

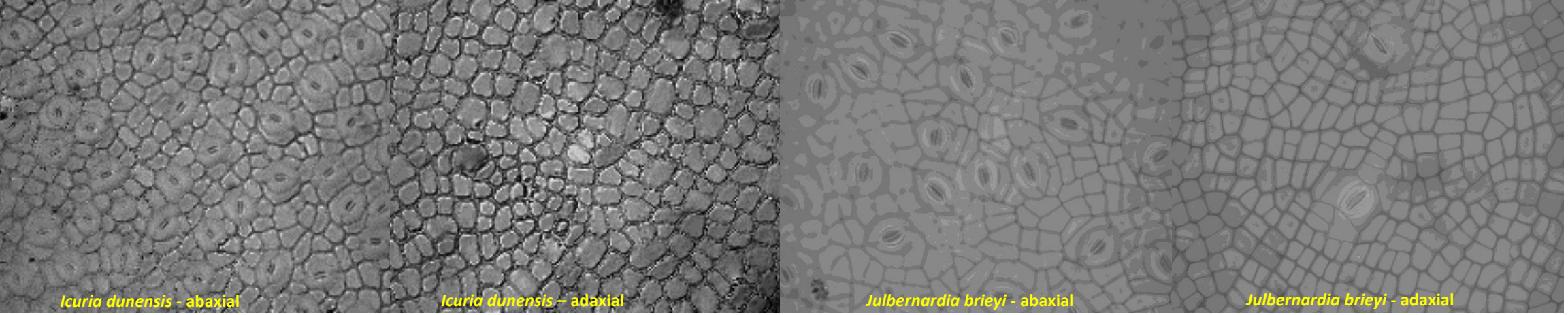

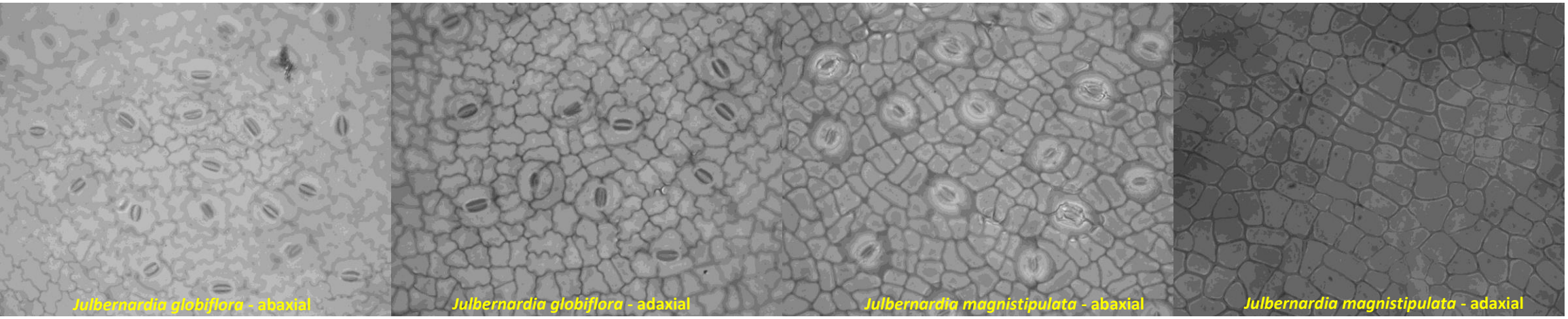

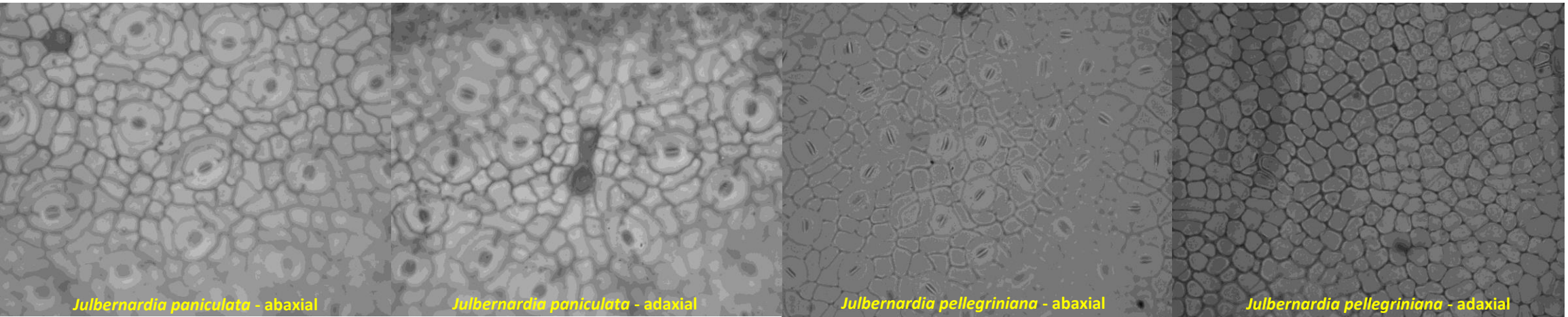

Berlinia Clade – Subclade A

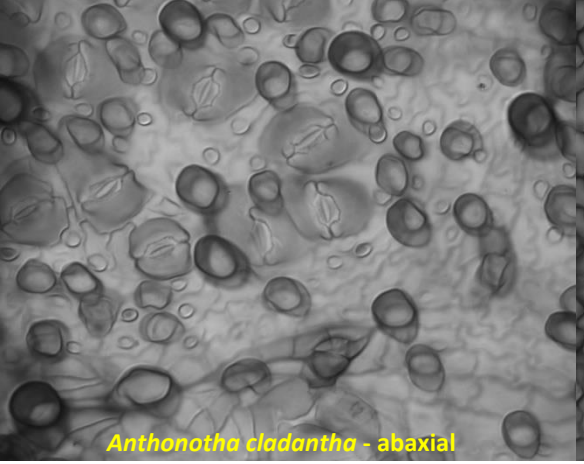
Anthonotha cladantha - abaxial

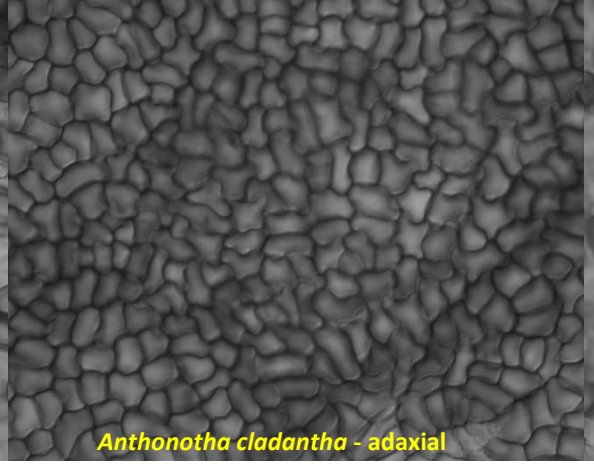
Anthonotha cladantha - adaxial

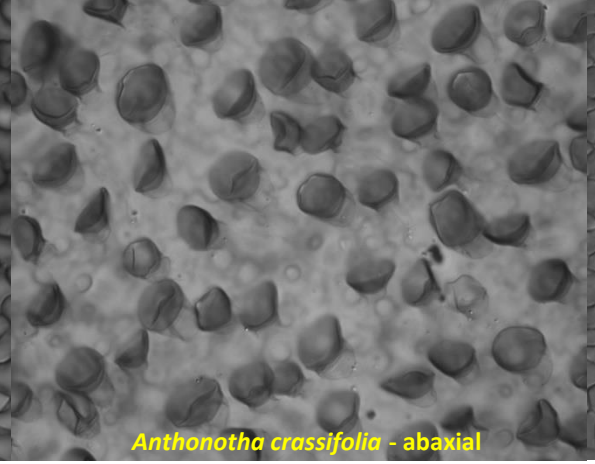
Anthonotha crassifolia - abaxial

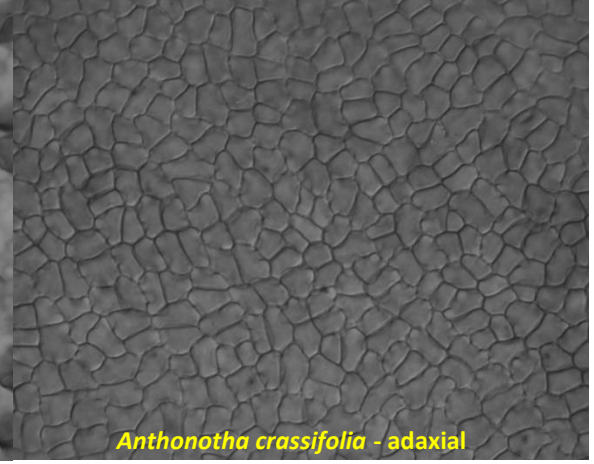
Anthonotha crassifolia - adaxial

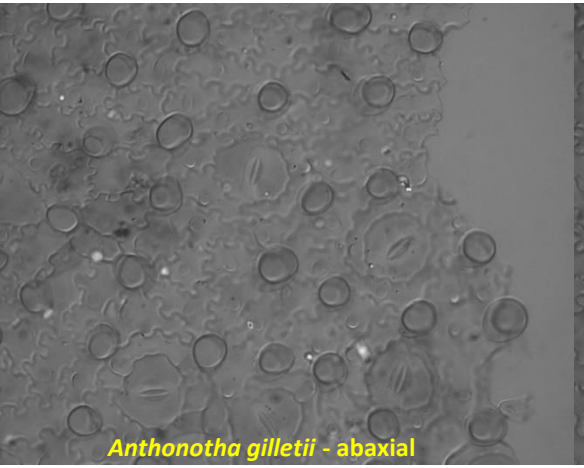
Anthonotha gilletii - abaxial

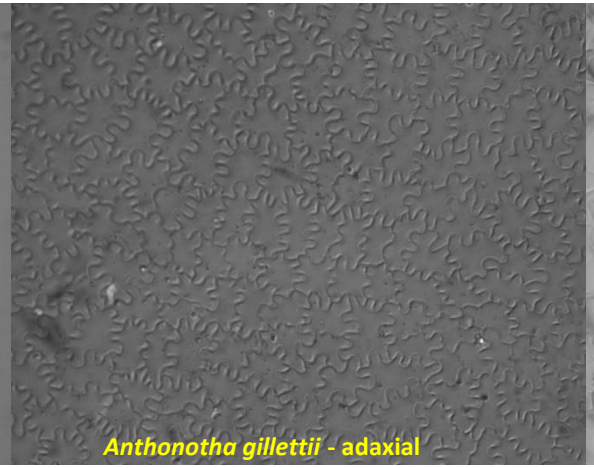
Anthonotha gillettii - adaxial

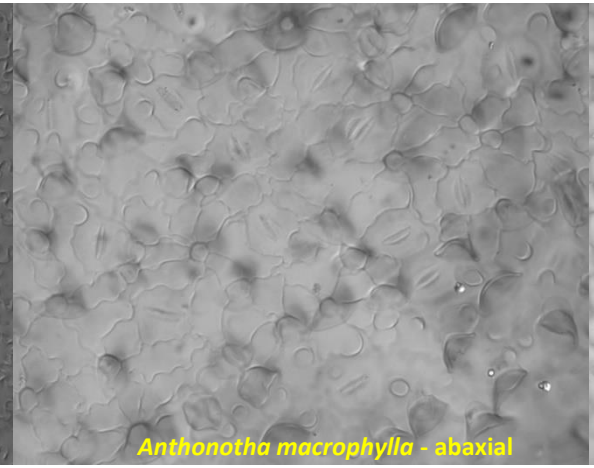
Anthonotha macrophylla - abaxial

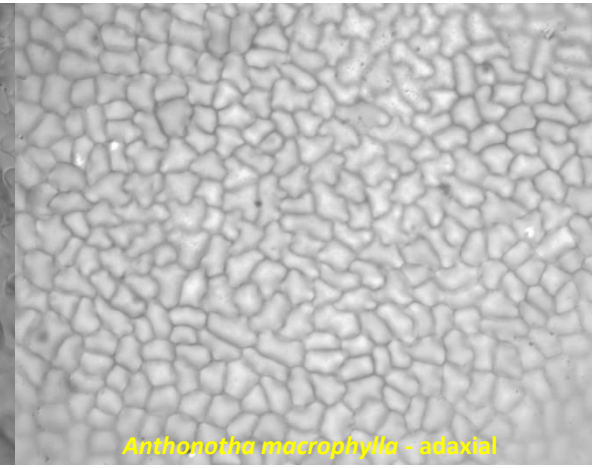
Anthonotha macrophylla - adaxial

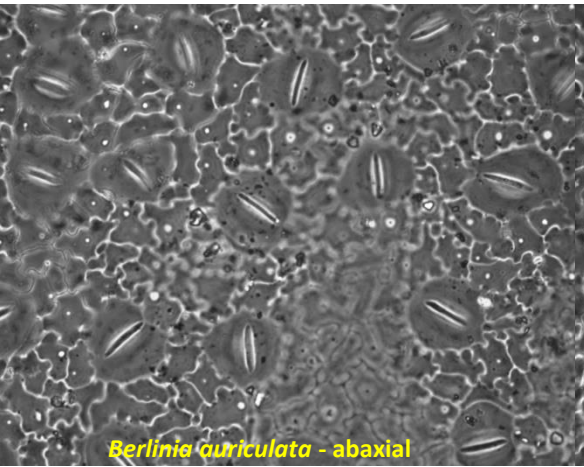
Berlinia auriculata - abaxial

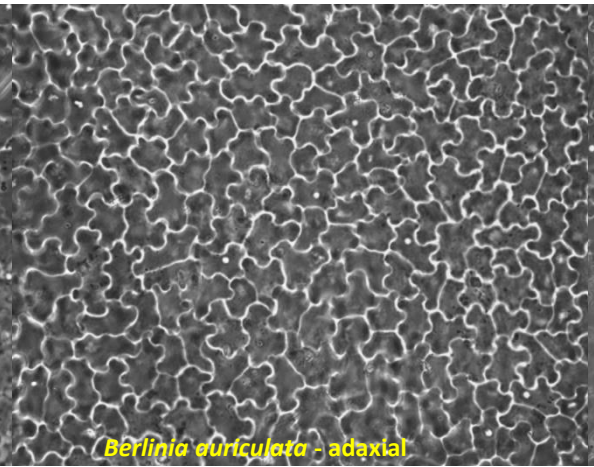
Berlinia auriculata - adaxial

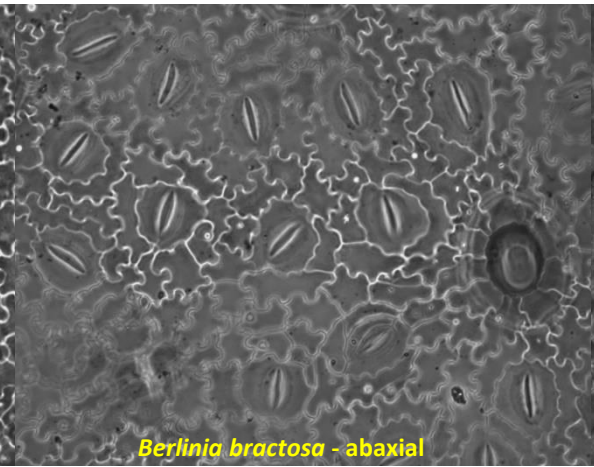
Berlinia bractosa - abaxial

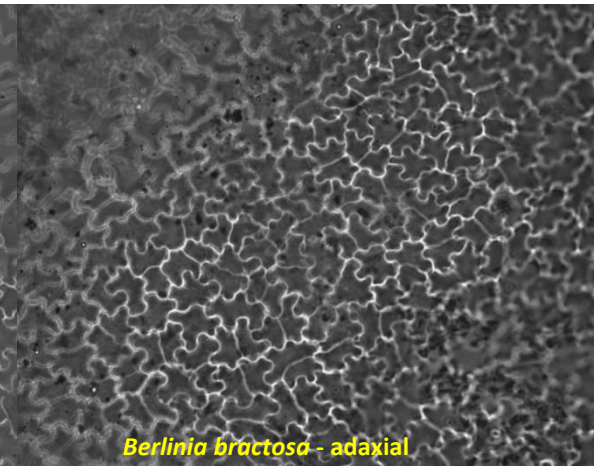
Berlinia bractosa - adaxial

Berlinia Clade – Subclade B

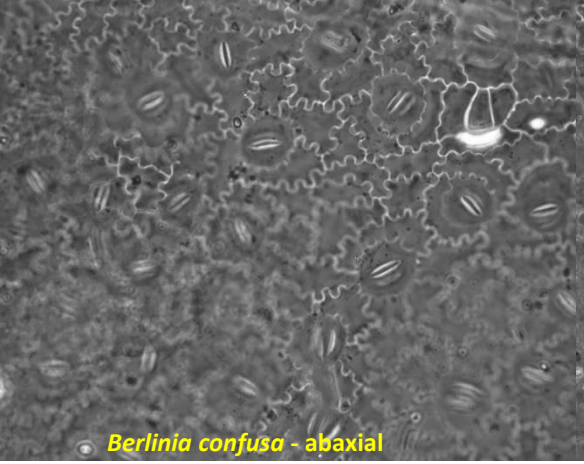
Berlinia confusa - abaxial

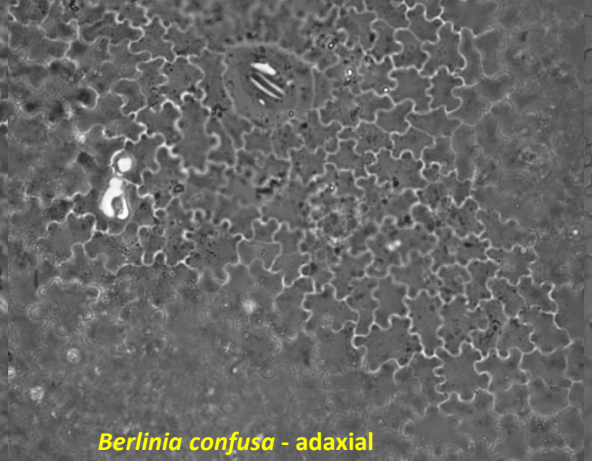
Berlinia confusa - adaxial

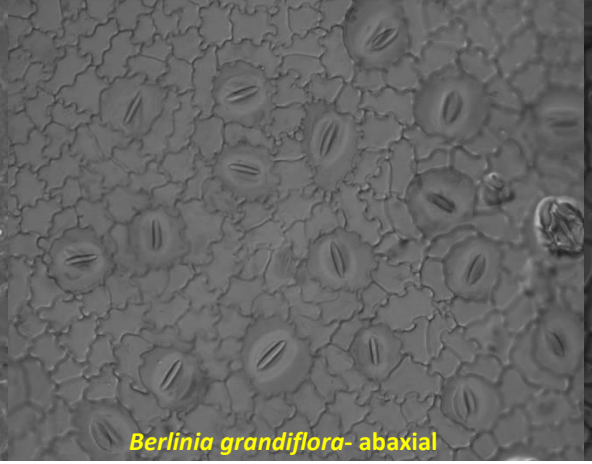
Berlinia grandiflora- abaxial

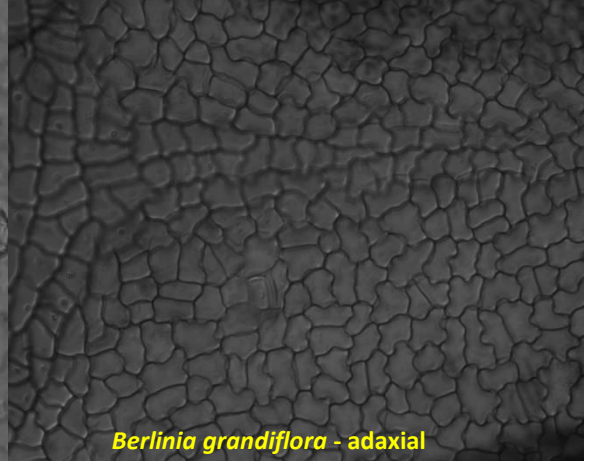
Berlinia grandiflora - adaxial

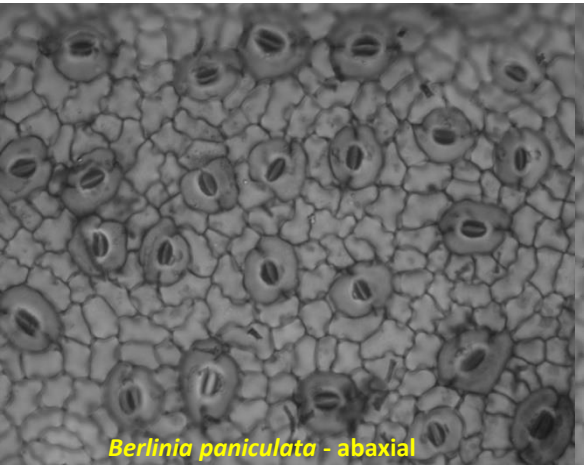
Berlinia paniculata - abaxial

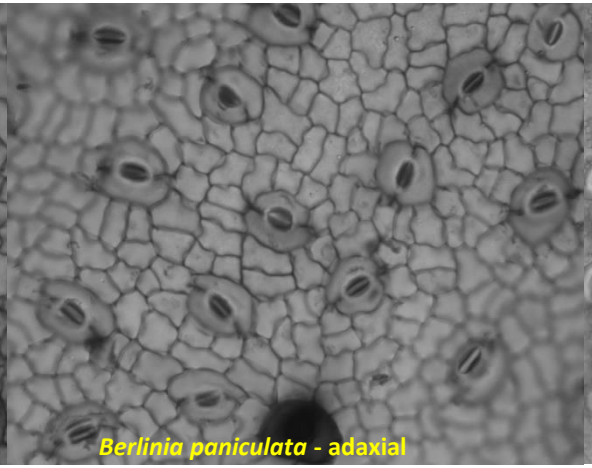
Berlinia paniculata - adaxial

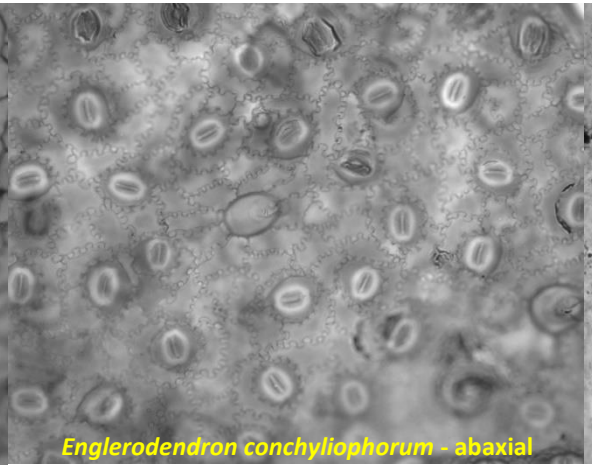
Englerodendron conchyliophorum - abaxial

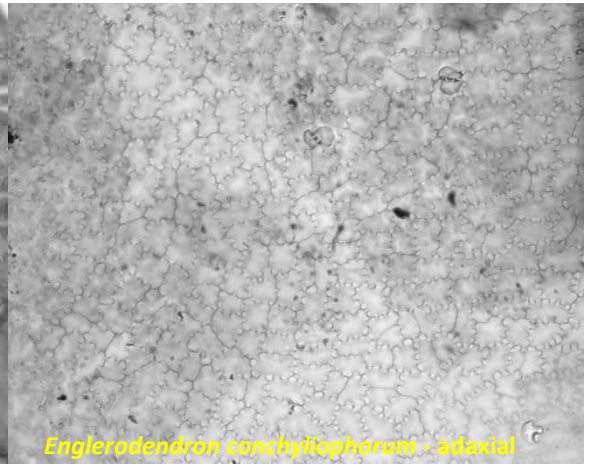
Englerodendron conchyliophorum - adaxial

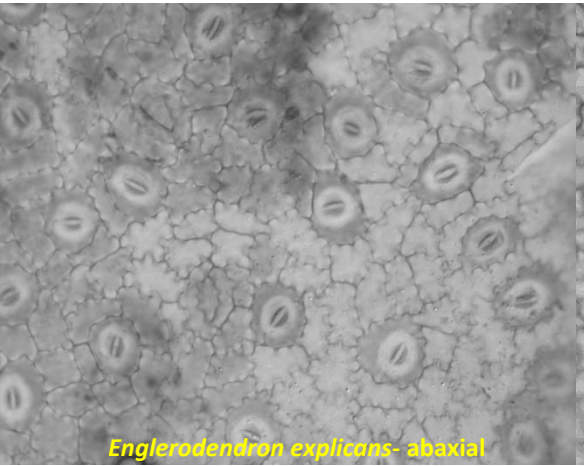
Englerodendron explicans- abaxial

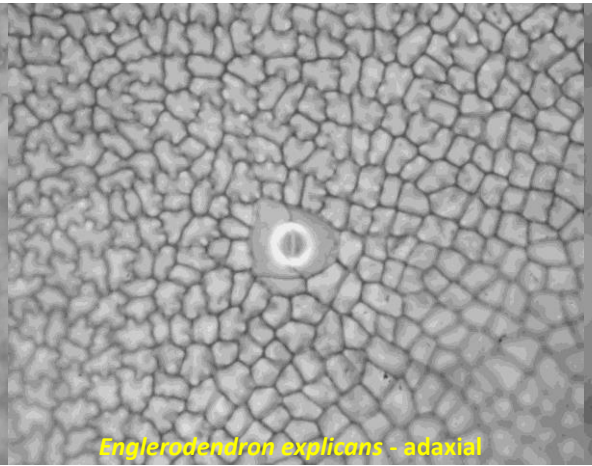
Englerodendron explicans - adaxial

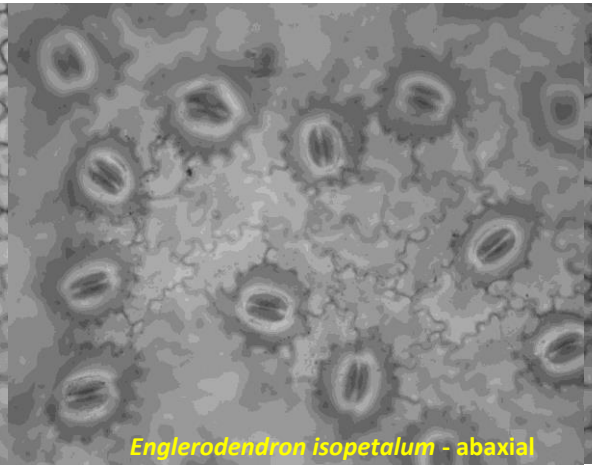
Englerodendron isopetalum - abaxial

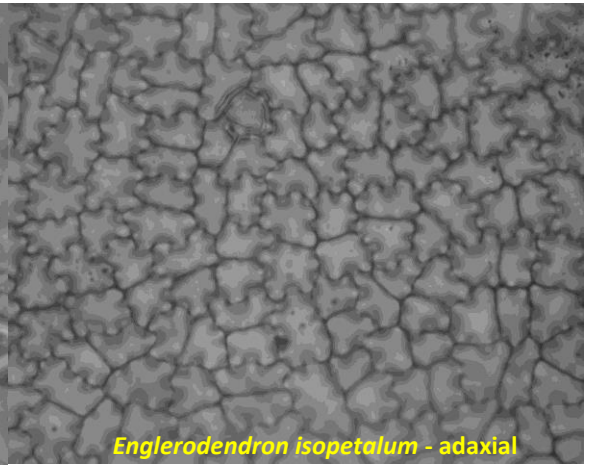
Englerodendron isopetalum - adaxial

Berlinia Clade – Subclade B

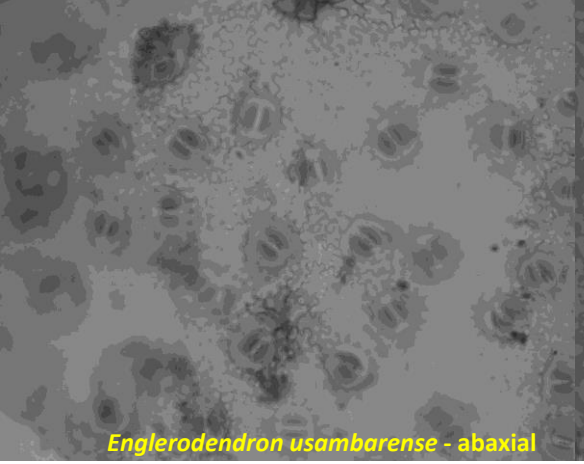

Englerodendron usambarense - abaxial

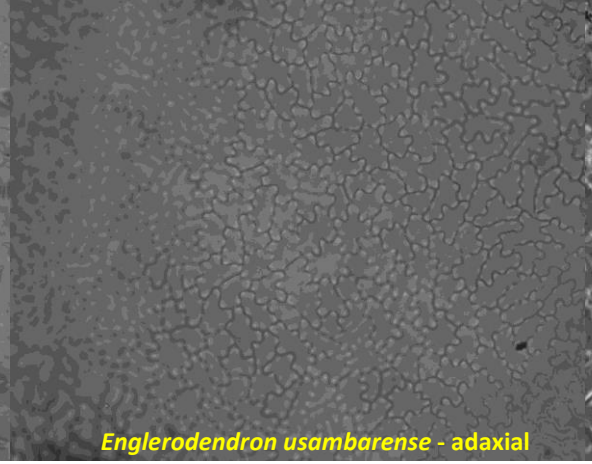

Englerodendron usambarense - adaxial

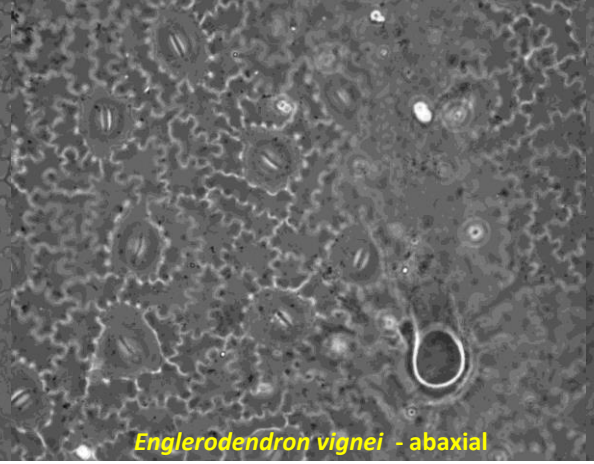

Englerodendron vignei - abaxial

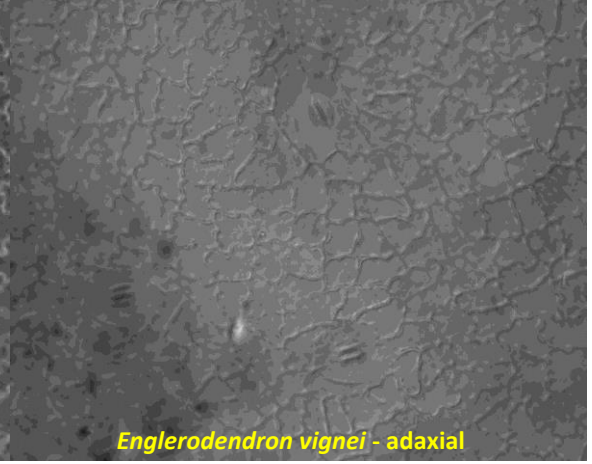

Englerodendron vignei - adaxial

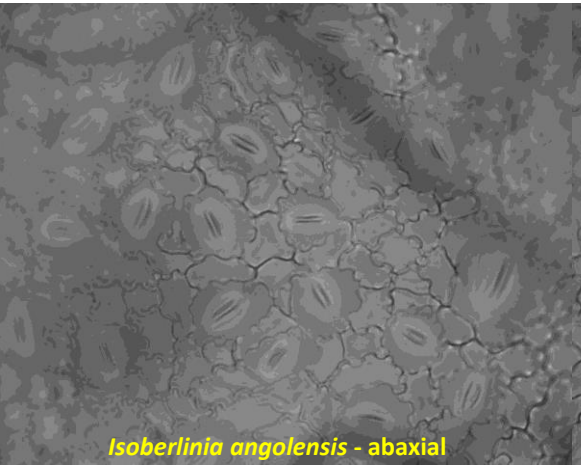

Isoberlinia angolensis - abaxial

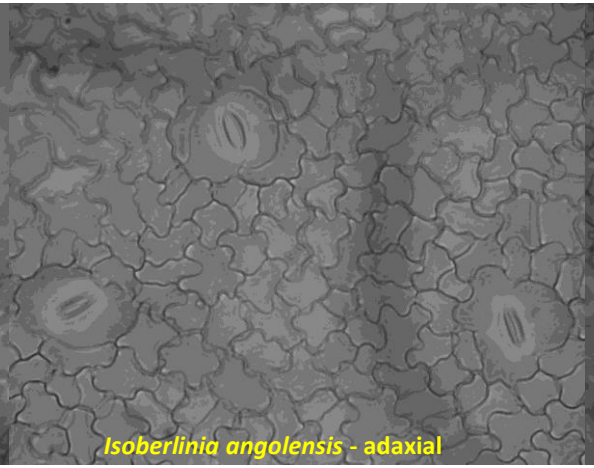

Isoberlinia angolensis - adaxial

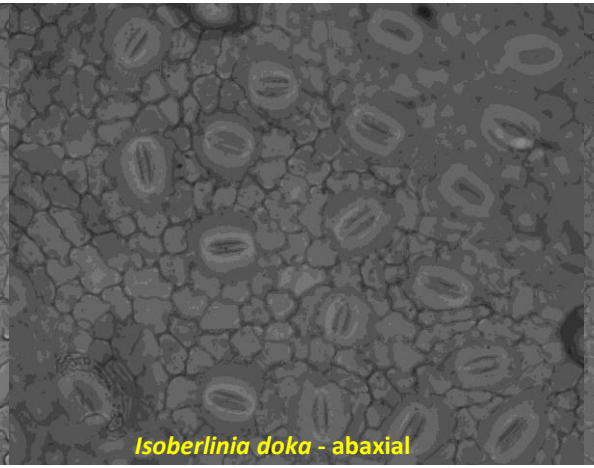

Isoberlinia doka - abaxial

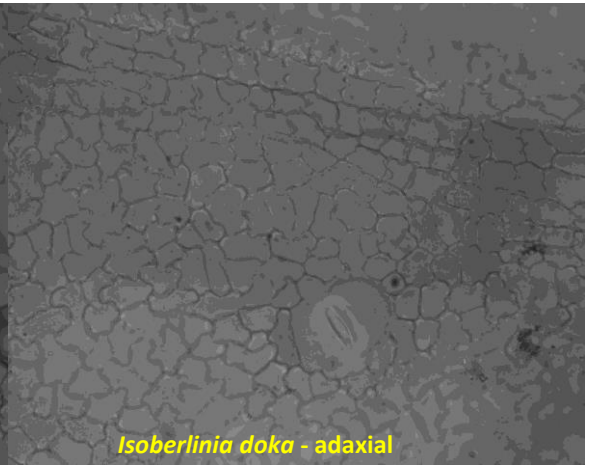

Isoberlinia doka - adaxial

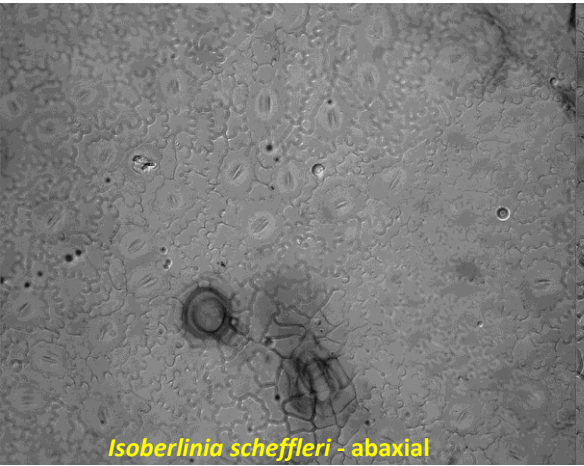

Isoberlinia scheffleri - abaxial

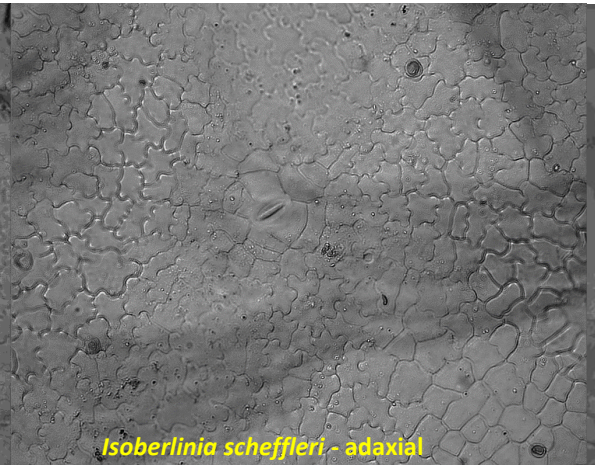

Isoberlinia scheffleri - adaxial

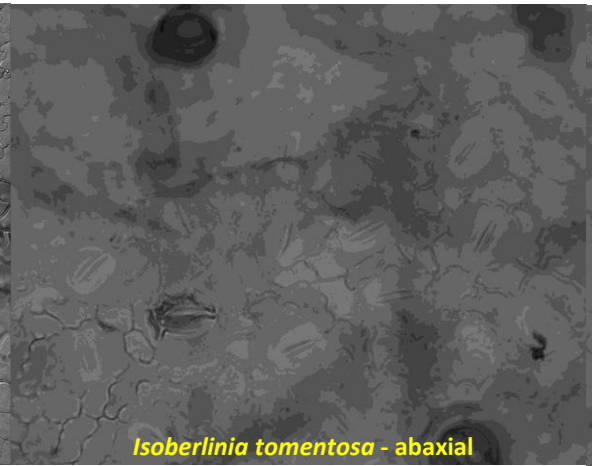

Isoberlinia tomentosa - abaxial

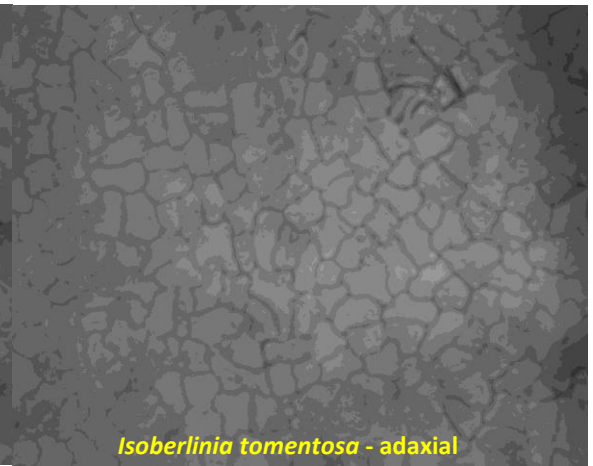

Isoberlinia tomentosa - adaxial

Berlinia Clade – Subclade B

Berlinia Clade – Subclade B

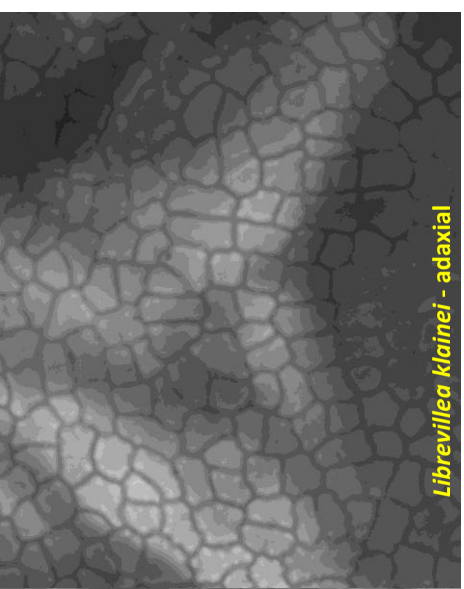

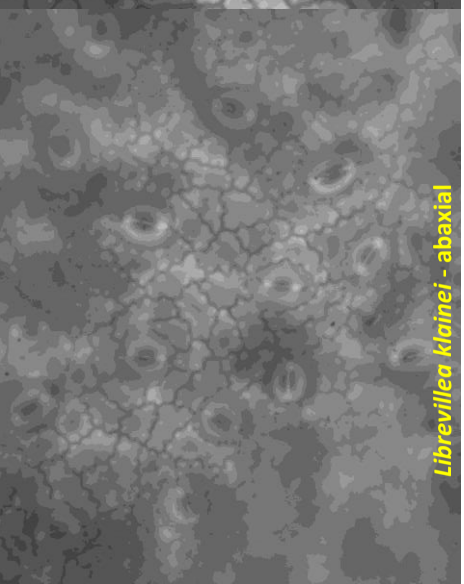

Supplement: S7 File — (PDF) [file pone.0279491.s007.pdf]
