# Supplementary material for: First evidence of a monodominant (Englerodendron, Amherstieae, Detarioideae, Leguminosae) tropical moist forest from the early Miocene (21.73 Ma) of Ethiopia
Source: PLoS One. 2023 Jan 11;18(1):e0279491. doi: 10.1371/journal.pone.0279491 (PMC9833558; doi:10.1371/journal.pone.0279491)
Supplement: Attachment — Submitted filename: PONE-D-22-26695_reviewer.pdf [file pone.0279491.s008.pdf]

PLOS ONE

FIRST EVIDENCE OF A MONODOMINANT (ENGLERODENDRON, AMHERSTIEAE, DETARIOIDEAE, LEGUMINOSAE) TROPICAL MOIST FOREST FROM THE EARLY MIOCENE (21.73 MA) OF ETHIOPIA

--Manuscript Draft--

**Manuscript Number:**	PONE-D-22-26695
**Article Type:**	Research Article
**Full Title:**	FIRST EVIDENCE OF A MONODOMINANT (ENGLERODENDRON, AMHERSTIEAE, DETARIOIDEAE, LEGUMINOSAE) TROPICAL MOIST FOREST FROM THE EARLY MIOCENE (21.73 MA) OF ETHIOPIA
**Short Title:**	MONODOMINANT FOREST FROM EARLY MIOCENE OF ETHIOPIA
**Corresponding Author:**	Aaron David Pan, Ph.D.<br>Texas Tech University<br>Lubbock, TX UNITED STATES
**Keywords:**	Africa; forest; forest succession; fossil; legume; Neogene; paleobotany
**Abstract:**	Many tropical wet forests are species-rich and have relatively even species frequency distributions. But, dominance by a single canopy species can also occur in tropical wet climates and can remain stable for centuries. These are uncommon globally, with the African wet tropics supporting more such communities than the Neotropics or Southeast Asia. Differences in regional evolutionary histories are implied by biogeography: most of Africa's monodominance-forming species are Amherstieae-tribe legumes; monodominance in Neotropical forests occur among diverse taxonomic groups, often legumes, but rarely Amherstieae, and monodominance in Southeast Asian forests occurs mostly among Dipterocarpaceae species. African monodominant forests have been characterized ecologically and taxonomically, but their deep-time history is unknown despite their significant presence and bottom-up ecological influence on diversity.<br>Herein we describe fossil leaflets of Englerodendron mulugeta sp. nov., an extinct species of the extant genus Englerodendron (Berlinia Clade, Amherstieae, Detarioideae) from the 21.73 Ma Mush Valley site in Ethiopia. We also document a detailed study of associated legume pollen, which originate from a single taxon sharing characters with more than one extant descendant. Taxonomically, the pollen is most comparable to that from some extant Englerodendron species and supports a likely affiliation with the Englerodendron macrofossils. The Mush Valley site provides the first fossil evidence of a monodominant tropical forest in Africa as represented by leaflets and pollen. Previous studies documented >2400 leaves and leaflets from localities at six stratigraphic levels spanning 50,000 – 60,000 years of nearly continuous deposition within seven meters of section; all but the basal level contain ≥ 50% E. mulugeta leaflets. Modern leaf litter studies in African mixed vs. monodominant forests indicates the likelihood of monodominance in the forests that surrounded the Mush paleolake, particularly after the basal level. Thus, we provide an early case for monodominance within the Amherstieae legumes in Africa.
**Order of Authors:**	Aaron David Pan, Ph.D.
	Bonnie Fine Jacobs, Ph.D.
	Rosemary T Bush, Ph.D.
	Manuel de la Estrella, Ph.D.
	Friðgeir Grímsson, Ph.D.
	Patrick S. Herendeen, Ph.D.
	Xander M. van der Burgt, Ph.D.
	Ellen D. Currano, Ph.D.
**Additional Information:**	

Powered by Editorial Manager® and ProduXion Manager® from Aries Systems Corporation

Question	Response
**Financial Disclosure**<br><br>Enter a financial disclosure statement that describes the sources of funding for the work included in this submission. Review the submission guidelines for detailed requirements. View published research articles from *PLOS ONE* for specific examples.<br><br>This statement is required for submission and **will appear in the published article** if the submission is accepted. Please make sure it is accurate.<br><br>**Unfunded studies**<br>Enter: *The author(s) received no specific funding for this work.*<br><br>**Funded studies**<br>Enter a statement with the following details:<br>• Initials of the authors who received each award<br>• Grant numbers awarded to each author<br>• The full name of each funder<br>• URL of each funder website<br>• Did the sponsors or funders play any role in the study design, data collection and analysis, decision to publish, or preparation of the manuscript?<br>• **NO** - Include this sentence at the end of your statement: *The funders had no role in study design, data collection and analysis, decision to publish, or preparation of the manuscript.*<br>• **YES** - Specify the role(s) played.<br><br>* typeset	Funded by U.S. National Science Foundation (https://www.nsf.gov/) grants EAR 1053549 (BFJ) and EAR 1052478 (EDC), National Geographic Society Grant (https://www.nationalgeographic.org/society/grants-and-investments/) CRE 8816-10 (EDC), and the Austrian Science Fund (https://www.fwf.ac.at/en/), FWF, P29501-B25 (FG). The funders had no role in study design, data collection and analysis, decision to publish, or preparation of the manuscript.
**Competing Interests**<br><br>Use the instructions below to enter a competing interest statement for this submission. On behalf of all authors, disclose any competing interests that could be perceived to bias this work—acknowledging all financial support and any other relevant financial or non-financial competing interests.	The authors have declared that no competing interests exist.

This statement is required for submission and **will appear in the published article** if the submission is accepted. Please make sure it is accurate and that any funding sources listed in your Funding Information later in the submission form are also declared in your Financial Disclosure statement.<br><br>View published research articles from *PLOS ONE* for specific examples.<br><br>**NO authors have competing interests**<br><br>Enter: *The authors have declared that no competing interests exist.*<br><br>**Authors with competing interests**<br><br>Enter competing interest details beginning with this statement:<br><br>*I have read the journal's policy and the authors of this manuscript have the following competing interests: [insert competing interests here]*<br><br>* typeset	
**Ethics Statement**<br><br>Enter an ethics statement for this submission. This statement is required if the study involved:<br><br>• Human participants<br>• Human specimens or tissue<br>• Vertebrate animals or cephalopods<br>• Vertebrate embryos or tissues<br>• Field research<br><br>Write "N/A" if the submission does not require an ethics statement.<br><br>General guidance is provided below. Consult the submission guidelines for detailed instructions. **Make sure that all information entered here is included in the Methods section of the manuscript.**	Field Research<br>Permit number: ARCCH (Authority for Research and Conservation of the Cultural Heritage) # 06/74-97/18<br>to conduct field work and collection of fossil plants and geologic specimens from the Upper and Lower Mush Valley, Amhara, Ethiopia.<br><br>All necessary permits were obtained for the described study, which complied with all relevant regulations.

Powered by Editorial Manager® and ProduXion Manager® from Aries Systems Corporation

**Format for specific study types**<br><br>**Human Subject Research (involving human participants and/or tissue)**<br>• Give the name of the institutional review board or ethics committee that approved the study<br>• Include the approval number and/or a statement indicating approval of this research<br>• Indicate the form of consent obtained (written/oral) or the reason that consent was not obtained (e.g. the data were analyzed anonymously)<br><br>**Animal Research (involving vertebrate animals, embryos or tissues)**<br>• Provide the name of the Institutional Animal Care and Use Committee (IACUC) or other relevant ethics board that reviewed the study protocol, and indicate whether they approved this research or granted a formal waiver of ethical approval<br>• Include an approval number if one was obtained<br>• If the study involved *non-human primates*, add *additional details* about animal welfare and steps taken to ameliorate suffering<br>• If anesthesia, euthanasia, or any kind of animal sacrifice is part of the study, include briefly which substances and/or methods were applied<br><br>**Field Research**<br><br>Include the following details if this study involves the collection of plant, animal, or other materials from a natural setting:<br>• Field permit number<br>• Name of the institution or relevant body that granted permission	
**Data Availability**<br><br>Authors are required to make all data underlying the findings described fully available, without restriction, and from the time of publication. PLOS allows rare exceptions to address legal and ethical concerns. See the PLOS Data Policy and FAQ for detailed information.	Yes - all data are fully available without restriction

A Data Availability Statement describing where the data can be found is required at submission. Your answers to this question constitute the Data Availability Statement and **will be published in the article**, if accepted.<br><br>**Important:** Stating 'data available on request from the author' is not sufficient. If your data are only available upon request, select 'No' for the first question and explain your exceptional situation in the text box.<br><br>Do the authors confirm that all data underlying the findings described in their manuscript are fully available without restriction?	
**Describe where the data may be found in full sentences. If you are copying our sample text, replace any instances of XXX with the appropriate details.**<br><br>• If the data are **held or will be held in a public repository**, include URLs, accession numbers or DOIs. If this information will only be available after acceptance, indicate this by ticking the box below. For example: *All XXX files are available from the XXX database (accession number(s) XXX, XXX.*).<br>• If the data are all contained **within the manuscript and/or Supporting Information files**, enter the following: *All relevant data are within the manuscript and its Supporting Information files.*<br>• If neither of these applies but you are able to provide **details of access elsewhere**, with or without limitations, please do so. For example:<br><br>*Data cannot be shared publicly because of [XXX]. Data are available from the XXX Institutional Data Access / Ethics Committee (contact via XXX) for researchers who meet the criteria for access to confidential data.*<br><br>*The data underlying the results presented in the study are available*	All relevant data are within the manuscript and its Supporting Information files.

from (include the name of the third party and contact information or URL).<br>• This text is appropriate if the data are owned by a third party and authors do not have permission to share the data.<br><br>* typeset	
Additional data availability information:	

Powered by Editorial Manager® and ProduXion Manager® from Aries Systems Corporation

Manuscript

Click here to access/download;Manuscript;Pan et al-Monodominant Eng - FINAL AND FORMATTED.docx

FIRST EVIDENCE OF A MONODOMINANT (*ENGLERODENDRON*, AMHERSTIEAE, DETARIOIDEAE, LEGUMINOSAE) TROPICAL MOIST FOREST FROM THE EARLY MIOCENE (21.73 MA) OF ETHIOPIA

Aaron D. Pan[1,2], Bonnie F. Jacobs[3], Rosemary T. Bush[4], Manuel de la Estrella[5], Friðgeir Grímsson[6], Patrick S. Herendeen[7], Xander M. van der Burgt[8], Ellen D. Currano[9]

1. Museum of Texas Tech University, Texas Tech University, 3301 4th Street, Lubbock, TX 79415, USA. https://orcid.org/0000-0002-3286-3105
2. Botanical Research Institute of Texas, 1700 University Drive, Fort Worth, TX 76107-3400, USA
3. Roy M. Huffington Department of Earth Sciences, Southern Methodist University, P0 Box 750395, Dallas, TX 75275-0395, USA. https://orcid.org/0000-0002-4770-0181
4. Department of Earth and Planetary Sciences, Northwestern University, 2145 Sheridan Road, Technological Institute, Evanston, IL 60208-3130, USA
5. Departamento de Botánica, Ecología y Fisiología Vegetal, Celestino Mutis, Campus de Rabanales, Universidad de Córdoba, E-14071, Córdoba, Spain. https://orcid.org/0000-0002-4484-3566
6. Department of Botany and Biodiversity Research, University of Vienna, Vienna, Austria. https://orcid.org/0000-0002-1874-6412
7. Division of Plant Science and Conservation, Chicago Botanic Garden, 1000 Lake Cook Road, Glencoe, IL 60022, USA. https://orchid.org/0000-0003-2657-8671
8. Africa Team, Herbarium, Royal Botanic Gardens, Kew, Richmond, TW9 3AE, United Kingdom. https://orcid.org/0000-0003-2712-3433
9. Department of Botany and Geology and Geophysics, University of Wyoming, 1000 E. University Avenue, Laramie, WY 82071, USA. https://orcid.org/0000-0002-5242-8573

ABSTRACT (300 words)

Many tropical wet forests are species-rich and have relatively even species frequency distributions. But, dominance by a single canopy species can also occur in tropical wet climates and can remain stable for centuries. These are uncommon globally, with the African wet tropics supporting more such communities than the Neotropics or Southeast Asia. Differences in regional evolutionary histories are implied by biogeography: most of Africa's monodominance-forming species are Amherstieae-tribe legumes; monodominance in Neotropical forests occur among diverse taxonomic groups, often legumes, but rarely Amherstieae, and monodominance in Southeast Asian forests occurs mostly among Dipterocarpaceae species. African monodominant forests have been characterized ecologically and taxonomically, but their deep-time history is unknown despite their significant presence and bottom-up ecological influence on diversity.

Herein we describe fossil leaflets of *Englerodendron mulugeta* sp. nov., an extinct species of the extant genus *Englerodendron* (Berlinia Clade, Amherstieae, Detarioideae) from the 21.73 Ma Mush Valley site in Ethiopia. We also document a detailed study of associated legume pollen, which originate from a single taxon sharing characters with more than one extant descendant. Taxonomically, the pollen is most comparable to that from some extant *Englerodendron* species and supports a likely affiliation with the *Englerodendron* macrofossils. The Mush Valley site provides the first fossil evidence of a monodominant tropical forest in Africa as represented by leaflets and pollen. Previous studies documented >2400 leaves and leaflets from localities at six stratigraphic levels spanning 50,000 – 60,000 years of nearly continuous deposition within seven meters of section; all but the basal level contain ≥ 50% *E. mulugeta* leaflets. Modern leaf litter studies in African mixed vs. monodominant forests indicates the likelihood of monodominance in the forests that surrounded the Mush paleolake, particularly after the basal level. Thus, we provide an early case for monodominance within the Amherstieae legumes in Africa.

INTRODUCTION

Modern moist and wet primary tropical forest formations are typically characterized by high to extremely high levels of biodiversity and can boast as many as 300 tree species within a single hectare [1, 2]. More unusual are natural moist and wet tropical forest formations that are dominated by a single tree species, often making up from 50% to nearly 100% of the canopy trees [3, 4]. While this phenomenon can be found in the tropics of Africa, the New World, and Asia, and monodominant species occur among at least 20 families and 47 genera worldwide, there are clear biogeographic differences among the three tropical regions. The majority of monodominant forests in the Neotropics and Africa are composed of species in the legume family (Fabaceae), but compared with Africa and Asia, Neotropical monodominant-forming forest taxa are more diverse; this is true whether considering all families or the diversity of subfamilies and tribes among legume dominants (Table 1) [5, 3, 6, 7]. African monodominant tropical forests are nearly always characterized by legume species in the tribe Amherstieae of the subfamily Detarioideae, many of which occur in the monophyletic and African endemic Berlinia Clade (Table 1) [8, 9]. A number of genera and species (even those not forming monodominant forest formations) within the Berlinia Clade have a tendency to form monotypic stands or are noted as often becoming gregarious where found [10] – thus, this African clade may have a propensity for forest ecological dominance. Tropical Asia is well-known and unique for the importance and Neogene diversity of the Dipterocarpaceae, and its monodominant forests are composed of species primarily in this family (Table 1) [11, 12].

Table 1. Monodominant Tropical Forests of the World and their dominant species component.

MONODOMINANT TROPICAL FOREST	SPECIES	HIGHER TAXONOMIC RANKINGS	CONTINENT
	Aucoumea klaineana	Burseraceae	AFRICA
	Terminalia superba	Combretaceae	AFRICA
	Aphanocalyx microphyllus	Fabaceae: Detarioideae: Amherstieae	AFRICA

	Brachystegia laurentii	Fabaceae: Detarioideae: Amherstieae	AFRICA
	Cynometra alexandri	Fabaceae: Detarioideae: Amherstieae	AFRICA
Mbau (*Gilbertiodendron dewevrei*) forest	*Gilbertiodendron dewevrei*	Fabaceae: Detarioideae: Amherstieae	AFRICA
Julbernardia seretii	*Julbernardia seretii*	Fabaceae: Detarioideae: Amherstieae	AFRICA
	Microberlinia bisulcata	Fabaceae: Detarioideae: Amherstieae	AFRICA
	Talbotiella gentii	Fabaceae: Detarioideae: Amherstieae	AFRICA
	Tetraberlinia bifoliolata	Fabaceae: Detarioideae: Amherstieae	AFRICA
	Tetraberlinia korupensis	Fabaceae: Detarioideae: Amherstieae	AFRICA
	Tetraberlinia tubmaniana	Fabaceae: Detarioideae: Amherstieae	AFRICA
	Gilletiodendron glandulosum	Fabaceae: Detarioideae: Detarieae	AFRICA
	Musanga cecropioides	Urticaceae	AFRICA
Kapur forest	*Dryobalanops aromatica*	Dipterocarpaceae	ASIA (Malesia)
	Parashorea malaanonan	Dipterocarpaceae	ASIA (Malesia)
	Shorea albida	Dipterocarpaceae	ASIA (Malesia)
	Shorea curtisii	Dipterocarpaceae	ASIA (Malesia)
Ulin forest	*Eusideroxylon zwageri*	Lauraceae	ASIA (Malesia)
	Palaquium gutta	Sapotaceae	ASIA (Malesia)
	Dimorphocalyx glabellus	Euphorbiaceae	ASIA (South Asia: Ind
	Strychnos nux-vomica	Loganiaceae	ASIA (South Asia: Ind
	Tricalysia sphaerocarpa	Rubiaceae	ASIA (South Asia: Ind
Sal forest	*Shorea robusta*	Dipterocarpaceae	ASIA (South Asia: Ind
	Hopea ferrera	Dipterocarpaceae	ASIA (Southeast Asia
	Backhousia bancroftii	Myrtaceae	AUSTRALIA
	Celaenodendron mexicanum	Euphorbiaceae	MESOAMERICA
	Quercus oleoides	Fagaceae	MESOAMERICA
	Pisonia grandis	Nyctaginaceae	OCEANIA (Micronesi
Ōhi'a forest	*Metrosideros polymorpha*	Myrtaceae	OCEANIA (Polynesia:
	Oxandra polyandra	Annonaceae	SOUTH AMERICA
	Astrocaryum macrocalyx	Arecaceae: Arecoideae: Cocoseae	SOUTH AMERICA
	Astrocaryum murumuru	Arecaceae: Arecoideae: Cocoseae	SOUTH AMERICA
	Attalea speciosa	Arecaceae: Arecoideae: Cocoseae	SOUTH AMERICA
	Euterpe oleracea	Arecaceae: Arecoideae: Euterpeae	SOUTH AMERICA

	Mauritia flexuosa	Arecaceae: Calamoideae: Lepidocaryeae	SOUTH AMERICA
	Jacaranda densicoma	Bignoniaceae	SOUTH AMERICA
	Tabebuia aurea	Bignoniaceae	SOUTH AMERICA
	Micrandra glabra	Euphorbiaceae	SOUTH AMERICA
	Mora gonggrijpii	Fabaceae: Caesalpinoideae	SOUTH AMERICA
	Mora oleifera	Fabaceae: Caesalpinoideae	SOUTH AMERICA
	Tachigali vaupesiana	Fabaceae: Caesalpinoideae	SOUTH AMERICA
	Mora excelsa	Fabaceae: Caesalpinoideae: *Dimorphandra* Group A	SOUTH AMERICA
	Dicymbe corymbosa	Fabaceae: Detarioideae: Amherstieae	SOUTH AMERICA
	Eperua falcata	Fabaceae: Detarioideae: Detarieae	SOUTH AMERICA
Jauacaná caatinga forest	*Eperua leucantha*	Fabaceae: Detarioideae: Detarieae	SOUTH AMERICA
Caatinga Forest (Jebarú)	*Eperua purpurea*	Fabaceae: Detarioideae: Detarieae	SOUTH AMERICA
	Peltogyne gracilipes	Fabaceae: Detarioideae: Detarieae	SOUTH AMERICA
cativales'	*Prioria copaifera*	Fabaceae: Detarioideae: Detarieae	SOUTH AMERICA
	Machaerium hirtum	Fabaceae: Faboideae: Dalbergieae	SOUTH AMERICA
	Spirotropis longifolia	Fabaceae: Faboideae: Ormosieae	SOUTH AMERICA
	Pentacletha macroloba	Fabaceae: Mimosoideae:	SOUTH AMERICA
	Vitex cymosa	Lamiaceae	SOUTH AMERICA
Igapó forest (black-water flooded forests)	*Eschweilera tenuifolia*	Lecythidaceae	SOUTH AMERICA
	Lueheopsis hoehnei	Malvaceae *sensu lato*	SOUTH AMERICA
	Pachira nitida	Malvaceae *sensu lato*	SOUTH AMERICA
	Brosimum rubescens	Moraceae	SOUTH AMERICA
	Phyllanthus elsiae	Phyllanthaceae	SOUTH AMERICA
	Triplaris weigeltiana	Polygonaceae	SOUTH AMERICA
	Ruizterania retusa	Vochysiaceae	SOUTH AMERICA

In addition to its biogeographic distinctiveness, Africa is unique among tropical regions with regard to the large areal extent of moist and wet monodominant forests [7, 13], which collectively occupy thousands of km^2 [5, 14, 15, 16]. They can be found in the Congo Basin to Cameroon and Nigeria and are dominated by, for example, *Gilbertiodendron dewevrei* (De Wild.) J.Léonard, 1952, *Julbernardia seretii* (De Wild.) Troupin, 1950, and *Brachystegia laurentii* (De Wild.) Louis ex J.Léonard, 1952 [5, 14, 15, 16]. A

number of causal mechanisms and species traits have been called upon to explain why in some circumstances monodominance emerges and then can persist for centuries. These include deep leaf litter and slow litter decomposition, large seed size, nutrient-poor soils, the presence of ectomycorrhizal symbionts, competitive advantages among the dominant taxa, and both shade and sun tolerance [4, 17, 18, 19]. Detarioideae legumes have a long evolutionary history in Africa and the Neotropics dating to the early Cenozoic [20, 21], one might expect monodominance to have occurred in the deep past, perhaps even in South America where this phenomenon is relatively rare today. None has been identified to date, leaving undocumented the origins and biogeographic history of monodominant tropical wet forests. In this paper, we describe an early Miocene taxon from Ethiopia (formerly referred to as "Legume 1" [22, 23]) and assign it to the Berlinia Clade. Previous work on the paleoecology of the locality that produced the new taxon found strong support for a forest community dominated by legume taxa overall, noting the relative abundance of "Legume 1" in particular, which comprises 1289 specimens (53%) of all leaves collected and documented (2427). Its long-term abundance through seven vertical meters of sampling prompted us to take a closer look at the structural nature of the forest community represented by all the fossil leaf assemblages to determine if monodominance is or is not represented at this locality, with the keystone role played by a Berlinia Clade taxon as happens in Africa today. We also looked at the dispersed pollen flora from the same sediments as the macrofossils to explore if Berlinia clade pollen occurs in the Mush record and if similar "monodominant" signals are reflected by the palynoflora.

MATERIALS AND METHODS

The Mush Valley fossil site is located in a region of the northwestern plateau of Ethiopia approximately 160 km northeast of Addis Ababa and between the towns of Debre Birhan and Debre Sina

[24, 25, 22, 23]. Abundant and diverse fossils including plants (leaf, wood, fruit, and seed compressions and pollen), vertebrates (rare mammals, anurans, and teleost fish compressions), and insect compressions are preserved in layers of lacustrine carbonaceous shale interbedded with thin layers of volcanic ash < ~3 cm thick [24, 25, 26, 23 27]; one exceptional interbedded ash horizon (fossil-bearing Level C) is 10 cm thick. The fossiliferous shales are constrained in age by $^{206}Pb/^{238}U$ dates on zircons within two volcanic ashes, one that is stratigraphically immediately below them and one five additional meters below that. These yield the following ages for the upper and lower ashes, respectively: 21.733 ± 0.060 Ma and 21.736 ± 0.015 Ma [28].

Fossil leaves and leaflets were quarried from benches excavated such that a 5 cm thick shale unit could be removed in large blocks. To retard desiccation and specimen damage, the blocks were wrapped and transported in trunks to the National Museum of Ethiopia after each field season and left to dry during intervals of about six months at a time. Subsequent splitting, preparation, photography, and classification took place at the museum for all leaves or leaflets having ≥ 50% of the lamina preserved. Small, ~0.5 cm^2, cuticle samples were removed from leaves to aid in classification. Fossil specimens are permanently housed at the National Museum of Ethiopia, Addis Ababa. Cuticle preparations and microscope slides from herbarium samples are housed in the Roy M. Huffington Department of Earth Sciences at Southern Methodist University, Dallas, TX, USA [24, 25, 23].

A total of 2427 leaves were classified (using morphotype names if not identified to taxon) from collections among two to three quarries at each of six stratigraphic levels (A-F) spanning 7 m of section [23]. A total of 49 morphotypes were classified, including "Legume 1", the most common taxon described and named in this paper (see also, [22]). Inventories of morphotype descriptions and number of specimens per quarry and level are provided in Currano et al. [23] (main text and supplemental materials). Frequency distributions, diversity measures, and isotopic measurements were analyzed to evaluate paleoecology and to identify any patterns of variation within and between stratigraphic levels,

thus gaining a spatial and temporal view of plant community characteristics [22, 23, 27]. The estimated time represented by these shale deposits is approximately one to three centuries per 5 cm-thick quarry collection, and approximately 50 – 60 kyrs in total, on the basis of comparison with dated Holocene and Pleistocene lake cores [23].

Fossil specimens were compared to extant Fabaceae specimens from herbaria housed at the United States National Museum of Natural History (US), Missouri Botanical Garden (MO), Botanical Research Institute of Texas (BRIT), and, via high resolution digital images from the Muséum national d'Histoire naturelle (MNHN) and Global Plants on JSTOR (S1). Additional comparisons were based on published material (citations in RESULTS). Methods for preparation of herbarium and fossil leaflet cuticle and venation are provided in Herendeen & Dilcher [29] and Pan et al. [25], respectively. Herbarium leaf cuticle slides are housed at the Chicago Botanic Garden, IL, USA.

Sedimentary samples for pollen analysis were collected from each of the stratigraphic levels (A-F) alongside the macrofossils. Sedimentary rock samples were processed and fossil pollen grains extracted according to the method explained in Grímsson et al. [30]. The fossil pollen grains were investigated both by light microscopy (LM) and scanning electron microscopy (SEM) using the single grain method as described by Zetter [31] and Halbritter et al. [32; pp. 121-123]. Ten fossil pollen grains were measured and studied with LM and SEM. For practical reasons, the fossil pollen is classified as a morphotype (MT) named after the locality where the grains were found. The fossil pollen from Mush is described in detail based on LM and SEM observations and compared to pollen from related extant taxa. Part of the original sediment and remaining organic residues from palynological preparations and the SEM stubs bearing the fossil pollen are curated in the Division of Structural and Functional Botany at the University of Vienna, Austria.

Pollen from extant flower material (S2) from the Royal Botanic Gardens, Kew (K) was prepared according to the protocol outlined in Grímsson et al. [33, 34] and Halbritter et al. [32; pp. 103-105,

Acetolysis the Fast Way]. Twenty-five pollen grains from each sample were measured and studied with LM and SEM. The pollen terminology follows Punt et al. [35; LM] and Halbritter et al. [32; SEM].

RESULTS

Fabaceae Lindley 1836

Detarioideae Burmeist. 1837

Amherstieae Benth. 1840

***Englerodendron* Harms 1907**

***Englerodendron mulugeta* Pan, Jacobs, Bush, Estrella, Grímsson, Herendeen, Burgt et Currano, *sp. nov*.**

Holotype: MU17-43 (Fig 1A). National Museum of Ethiopia, Addis Ababa

Paratypes: MU 23-37#1 (Cuticle), MU27-18#1 (Fig 1C), and MU29-40B #1 (Figs 1D)

Additional Material: MU7-6#3, MU7-10, MU7-21#1, MU7-35-A5A#23, MU7-40#5, MU13-1, MU13-28#1, MU13-28#3, MU17-26A, MU18-16A#1, MU23-20, MU23-59#1, and MU32-22#8

Diagnosis: Paripinnate leaves, symmetrical leaflets, oppositely inserted with twisted and terete pulvinate petiolules and acuminate leaflet tips. Secondary venation is eucamptodromous, often becoming brochidodromous towards the leaflet apex. Abaxial and adaxial cuticle with highly sinuous anticlinal cell walls, which also possess ‘knob-like’ thickenings. Stomata paracytic. Trichome bases present, but sparse.

Fig 1. Fossil and extant *Englerodendron* lamina

A. *Englerodendron mulugeta* sp. nov. paripinnate compound leaf (the twin leaflet is missing) with twisted pulvinate petiolule. HOLOTYPE– MU17-43

B. Terminal leaflet of paripinnate leaf of *Englerodendron korupense* the twin leaflet is missing). ISOTYPE – MNHN-P-P00634905. Scale bar – 10 mm. Note twisted pulvinate petiolule

C. Leaflet of *Englerodendron mulugeta* sp. nov. with detail of twisted pulvinate petiolule. MU27-18 #1. Scale bar – 10 mm

D. *Englerodendron mulugeta* sp. nov Leaflet with pulvinate petiolule. MU29-40B #1

E. Leaflet of *Englerodendron vignei* . MNHN-P-P01037798. Scale bar – 10 mm

Description: Modified and updated from Bush et al. [22]. Paripinnate leaves, possessing oppositely inserted symmetrical unlobed, untoothed (entire-margined), elliptic to slightly falcate leaflets with twisted and terete pulvinate short petiolules (Fig 1). Leaflet lamina generally range in size from circa 4.5 to 15.0 cm in length and 1.6 to 5.7 cm in width, typically with length: width ratios of 3:1 to 5:2. Leaflet midribs are canaliculate. The leaflet apex is acute and possesses an acuminate 'drip' tip. The leaflet base is straight to rounded in shape and ranges from symmetrical to slightly asymmetrical [22; Fig 1D]. Primary venation is pinnate (Fig 1). Secondary venation is eucamptodromous, often becoming brochidodromous distally [22; Fig 1]. The secondary veins are irregularly spaced, have excurrent attachment, and generally possess uniform acute angles (Fig 1). Leaflets typically possess (5 –) 6 – 7 (9 – 10) pairs of secondary veins. Inter-secondary veins are present, diverge from the midvein parallel to the secondary veins, are less than 50% of the length of the subjacent secondary, and have a frequency of < 1 per intercostal area (Fig 1). Tertiary venation is sinuous and generally opposite percurrent, rarely alternate percurrent (Fig 1). Fourth order veins are irregular reticulate, and fifth order veins freely ramify. CUTICLE: Stomatal complexes are paracytic (incorrectly identified as pericytic in Bush et al., [22]) with one subsidiary cell larger than the other (Fig 2A). Stomata are prominent on the abaxial surface and are rare on the adaxial surface (Fig 2E). Guard cells range in size from 7.5 to 10.5 µm in length [23; Fig 2A]. The anticlinal cell walls on both the abaxial and adaxial leaf surfaces are highly sinuous [22], typically with greater than 9 'folds' and often numbering ≥11 (Fig 2A, E). The abaxial epidermal cells are rectangular in shape and are 21.3 to 32.7 µm in length and 9.8 to 14.1 µm in width [22; Fig 2A]. Papillae are absent from both abaxial and adaxial leaf epidermal periclinal cell surfaces, but knob-like thickenings

appear to be present on the anticlinal walls (Fig 2A, E.; [36]). Hairs (trichomes) are present on the abaxial surface, but sparse, based on presence of thickened trichome bases (Fig 2A). Trichomes are commonly associated with the veins. Hair bases present in intercostal areas are surrounded by radial basal cells (Fig 2A). Hair bases associated with veins are surrounded by vein epidermal cells oriented similarly to adjacent vein cells (Fig 2A).

Fig 2. Fossil and extant *Englerodendron* lamina cuticle

A. *Englerodendron mulugeta* abaxial leaflet surface. MU 23-37#1

B. *Englerodendron conchpliophorum* abaxial leaflet surface. BM000081672

C. *Englerodendron usambarense* abaxial leaflet surface. K H/1194/96/56.

D. *Englerodendron isopetalum* abaxial leaflet surface. BM000081670

E. *Englerodendron mulugeta* sp. nov. adaxial surface. MU 23-37#1

F. *Englerodendron concyliophorum* adaxial surface. BM000081672

<u>Etymology:</u> The species name provides two inferences, ‘mulugeta’ means ‘he who rules’ in Amharic, referring to the ecological and temporal dominance of the species in the early Miocene Mush paleoenvironment and the species name also honors our friend and colleague, Dr. Mulugeta Feseha, of Addis Ababa University, who introduced us to the fossil locality.

<u>Comments:</u> Based on a suite of macro-morphological leaf characteristics, the fossil morphotype originally designated as “Legume 1” [22, 23] can be identified taxonomically as a member of the Berlinia Clade of the tribe Amherstieae (Fabaceae: Detarioideae), and specifically matches a number of genera in the Berlinia Clade: Subclade B, namely *Anthonotha*, *Berlinia*, *Englerodendron*, and *Isoberlinia* [37] (S3).

These characteristics include paripinnate compound leaves, oppositely inserted, symmetrical leaflets that are elliptic to slightly falcate in shape, the presence of a short twisted pulvinate petiolule, a symmetrical to slightly asymmetrical base, secondary venation being eucamptodromous becoming brochidodromous distally towards the apex, and an acuminate leaflet apex forming a drip tip [38, 22, 39] (Fig 1; S3) The fossil morphotype differs from *Oddoniodendron*, another member of Berlinia Clade Subclade B, in lacking a long petiolule and in not possessing alternately inserted leaflets [40, 37]. The African endemic moist forest genus *Gilbertiodendron* is similar to the fossil taxon and possesses many of the characteristics mentioned above, but differs from the Mush taxon in possessing leaflets with brochidodromous to festooned brochidodromous secondary venation and the presence of leaf marginal and/or submarginal glands [38, 41, 39, 42]. Other members in the Berlinia Clade, particularly genera in the 'bambjit' clade (*Brachystegia*, *Aphanocalyx*, *Michelsonia*, *Bikinia*, *Icuria*, *Tetraberlinia*) and *Microberlinia* possess asymmetrical leaflets [43, 38, 37] (S3), have a major basal vein or 'fan' of veins, and often have retuse or emarginate leaflet apices, all of which are absent from the fossil taxon. These characteristics commonly occur in a number of other Amherstieae taxa as well, and so taxa such as *Cynometra* clade A, *Cynometra* clade B, *Hymenostegia*, *Plagiosiphon*, *Scorodophloeus*, and *Talbotiella* can be eliminated as potential candidates for the fossil taxon [44, 38, 45, 46](S3). The presence of twisted pulvinate petiolules is a relatively rare character and can only be found in a handful of Amherstieae genera including *Crudia*, *Berlinia*, *Englerodendron*, *Gilbertiodendron*, *Librevillea*, *Oddoniodendron*, *Paramacrolobium*, and *Isoberlinia*. The fossil morphotype differs from *Crudia* leaflet symmetry, secondary venation (*Crudia* possesses brochidodromous/festooned brochidodrmous venation), and leaves (alternately inserted leaflets in *Crudia*); *Librevillea*, in possessing alternately inserted, asymmetrical leaflets and imparipinnate leaves; *Paramacrolobium* in leaflet symmetry and lacking a major basal vein; and *Gilbertiodendron* and *Oddoniodendron* in the characteristics noted above [38] (S3). It should be noted that *Gilbertiodendron*, *Berlinia, Englerodendron*, and *Isoberlinia* can possess

both twisted and terete petiolules on the same plant or even compound leaf [38], S3), unlike *Crudia*, where the character is constant. *Anthonotha*, *Berlinia*, *Englerodendron*, and *Isoberlinia* are the four genera having macro-morphological characteristics most similar to the fossil taxon, and among these, *Berlinia* and *Englerodendron* are the most closely comparable. *Isoberlinia* differs in typically possessing leaflets with noticeably asymmetrical leaflet bases and while leaflet apices may be acuminate (typically acute or rounded in savannah taxa), 'drip tips' are not present or not elongate.

The leaf epidermal cuticle characteristics allow the fossil to be placed in the genus *Englerodendron* as opposed to *Anthonotha*, *Berlinia*, and *Isoberlinia*. *Englerodendron*, and the more distantly related *Gilbertiodendron*, have highly sinuous anticlinal cell walls on both abaxial and adaxial leaf epidermal surfaces among species observed (Fig 2; S4). The undulations on the anticlinal cell walls in species of these two genera are typically 'Ω' in shape (Fig 2; S4). *Berlinia* possess sinuous anticlinal cell walls, but the undulations and fold amplitude are not as significant as in the fossil taxon, *Englerodendron*, or *Gilbertiodendron* and undulations are generally '∩' in shape [47] (S4). *Isoberlinia* anticlinal cell walls are also undulate, but not usually sinuous (although, *Isoberlinia scheffleri* can possess sinuous anticlinal cell walls; S4). *Anthonotha* differs from the fossil in being characteristically hairy on the abaxial leaf surface and in possessing papillae on abaxial periclinal cell surfaces (S4).

Mush Morphotype (MT) pollen, affinity close to *Englerodendron conchyliophorum* and *E. korupense*

<u>Description:</u> Pollen, monad, prolate, P/E ratio 1.3-1.8, shape lobate-ellipsoid, elliptic in equatorial view, trilobate in polar view; equatorial diameter 17-42 µm in LM, 20-36 µm in SEM, polar axis 30-53 µm in LM, 35-61 µm in SEM; tricolporate; colpi long, extending between poles; pori inconspicuous, elongate elliptic(LM); exine 0.8-1.8 µm thick, nexine thinner than sexine (LM); tectate, colmellate (SEM); sculpture striate in LM, striate, perforate in SEM; aperture membrane granulate, nano- to microgemmate/areolate/rugulate, rugulate (SEM) (Figure 3, Table 2).

Table 2. Pollen morphology of fossil and extant Berlinia Clade pollen investigated for this study

	Mush MT (fossil pollen)	*Englerodendron conchyliophorum*	*Englerodendron explicans*	*Englerodendron korupense*	*Englerodendron leptorrhachis*
Equatorial diameter (LM)	17-42	**40-48**	**35-38**	**40-46**	**38-42**
Equatorial diameter (SEM)	20-36	**28-43**	**32-35**	**34-44**	**34-41**
Polar axis (LM)	30-53	**48-52**	**36-41**	**52-56**	**34-38**
Polar axis (SEM)	35-61	**47-62**	**41-47**	**51-60**	**33-39**
P/E ratio measured (LM)	1.3-1.8	**1-1.3**	1-1.1	**1.1-1.4**	0.8-1
P/E ratio category (LM)	Prolate	Isodiametric to **prolate**	Isodiametric to **prolate**	**Prolate**	Oblate to Isodiametric
Outline in equatorial view (LM)	Elliptic	Circular to **elliptic**	Circular to **elliptic**	**Elliptic**	Circular to elliptic
Outline in polar view (LM)	Trilobate	**Trilobate**	**Trilobate**	**Trilobate**	Circular (to **trilobate**)
Pollen shape	Lobate-ellipsoid	**Lobate**-spheroid to **ellipsoid**	**Lobate-**spheroid to **ellipsoid**	**Lobate-ellipsoid**	Ellipsoid to spheroid
Exine thickness (LM)	0.8-1.8	**1.6-2.6**	**1.8-2.4**	**1.6-2.4**	**1.2-1.6**
Sculpture LM	Striate	**Striate**	**Striate**	**Striate**	**Striate**
Sculpture SEM	Striate, perforate	**Striate, perforate**	**Striate**	**Striate, perforate**	**Striate**
Striae width (SEM)	0.8-1.5	**0.5-1.7**	**1.2-1.6**	**1.2-2**	**0.4-1.1**
Apertures	Tricolporate	**Tricolporate**	**Tricolporate**	**Tricolporate**	**Tricolporate**
Visability pori (LM)	Inconspicuous	**Inconspicuous**	**Inconspicuous**	Conspicuous to **inconspicuous**	Conspicuous
Outline pori (LM)	Lolongate elliptic	**Lolongate elliptic**	**Lolongate elliptic**	**Lolongate elliptic**	**Lolongate elliptic**
Aperture membrane (SEM)	Granulate, nano- to microgemmate / areolate / rugulate, rugulate	**Granulate, nano- to microrugulate**	**Nanorugulate to regulate**	**Granulate, nanorugulate to rugulate**	**Granulate, nano- to microareolate / rugulate**
Overlapping features (LM & SEM)	N/A	17	16	17	12

Note: All measurements include only those from this study and are given in µm. Features of extant pollen that overlap with the fossil Mush MT appear in bold font. N/A = not applicable.

<u>Comparison:</u> The Mush MT pollen shows a considerable size range when considering the equatorial diameter and length of the polar axis, also many of the grains are small. None of the pollen from the

extant taxa shows such a wide size range nor are as small as the fossil pollen, but the Mush MT overlaps with most of them, and all size ranges measured for *Englerodendron explicans* pollen fall within the fossil MT. The Mush MT is clearly prolate, with a P/E ratio between 1.3-1.8, but pollen from most of the extant taxa are close to isodiametric, except for pollen of *E. conchyliophorum* and *E. korupense*, which are also prolate (Figs 3 – 5; Table 2). The Mush MT is trilete in polar view and lobate-ellipsoid in shape like pollen from most of the extant taxa, except pollen of *Oddoniodendron micranthum*, which is hexagonal in polar view and hexagonal-dipyramid in shape (Fig 3; Table 2). The exine thickness of the Mush MT pollen displays a wide range, consistent with the size range of the morphotype (Table 2). The exine thickness overlaps with measurements from all the extant taxa, except *Berlinia orientalis*, which has a considerably thicker exine (Table 2). The exine thickness of *Englerodendron leptorrhachis* falls completely within the Mush MT, and the exine thickness of *E. usambarense* is also very close to that of the fossil MT. All pollen from extant *Englerodendron*, *Berlinia*, and *Isoberlina*, are striate when observed with LM, in the same way as the fossil MT, but pollen of *Oddoniodendron micranthum* is rugulate and clearly different when observed with either LM or SEM (Table 2). Only *Englerodendron conchyliophorum*, *E. korupense*, and *E. usambarense* show the perforate sculpture between the striae observed with SEM that is typical for the Mush MT. The width of the striae in the Mush MT overlaps with that of all investigated extant taxa except *Oddoniodendron*, which has a rugulate and verrucate sculpture when observed with SEM. The width of the striae in *Englerodendron conchyliophorum* and *E. usambarense* is close to that of the Mush MT, but the width of striae in *Isoberlinia scheffleri* is identical to that of the fossil. Both the Mush MT and pollen from all the investigated extant taxa is tricolporate, but the fossil pollen grains have inconspicuous pori that are hardly observed in either LM or SEM (Fig 3 – 5; Table 2). This feature was also noticed for *Englerodendron conchyliophorum*, *E. explicans*, and *E. korupense* (Fig 3 – 4; Table 2). The pori are lolongate elliptic in outline in the Mush MT and in pollen from most of the extant taxa, except *Oddoniodendron micranthum*, where the pollen is lalongate elliptic. The sculpture of

the aperture membrane in the Mush MT is quite variable and overlaps with that observed in pollen from all the extant taxa. The combined features within each taxon shows that the Mush MT shares most morphological features with pollen of *Englerodendron conchyliophorum* (17 traits), *E. korupense* (17 traits), and *E. explicans* (16 traits), followed by *Isoberlinia scheffleri* (15 traits; Table 2).

Fig 3. Light microscopy (A–F) and scanning electron microscopy (G–O) micrographs of the fossil Mush *Englerodendron* MT pollen, from Mush Valley, Ethiopia, Africa

A-E. Equatorial views, compressed pollen grains.

F–G. Polar view, same compressed grain in LM and SEM.

H–K. Equatorial views, compressed prolate pollen grains.

L. Close-up of interapertural area, showing striate sculpture.

M. Close-up of (J), showing interapertural area with striate and perforate sculpture.

N. Close-up of (G), showing aperture with granulate and nano- to microgemmate/areolate sculpture membrane.

O. Close-up of aperture, showing nanorugulate to regulate sculpture membrane. Scale bars – 10 µm (A–K), 1 µm (L–O).

Fig 4. Light microscopy (A, B, E, F, I, J, M, N) and scanning electron microscopy (C, D, G, H, K, L, O, P) micrographs of extant Berlinia Clade pollen from Africa

A–D. *Berlinia orientalis* (from Tanzania, coll. FC Magogo & R Rose Innes, 409 [K000023099]). A. Polar views, high focus (left), optical cross-section (right). B. Equatorial views, high focus (left), optical cross-

section (right). C. Equatorial view. D. Close-up showing aperture membrane and adjacent striate sculpture.

E–H. *Englerodendron conchyliophorum* (from Cameroon, coll. s.n., s.n. [K000557101]). E. Polar views, high focus (left), optical cross-section (right). F. Equatorial views, high focus (left), optical cross-section (right). G. Equatorial view. H. Close-up showing aperture membrane and adjacent striate and perforate sculpture.

I–L. *Englerodendron explicans* (from Guinea, coll. Haba, 1257 [K001381154]). I. Equatorial views, high focus (left), optical cross-section (right). J. Equatorial views, high focus (left), optical cross-section (right). K. Equatorial view. L. Close-up showing aperture membrane and adjacent striate sculpture.

M–P. *Englerodendron korupense* (from Cameroon, coll. XM van der Burgt [K000264712]). M. Polar views, high focus (left), optical cross-section (right). N. Equatorial views, high focus (left), optical cross-section (right). O. Equatorial view. P. Close-up showing aperture membrane and adjacent striate sculpture. Scale bars – 10 µm (A–C, E–G, I–K, M–O), 2 µm (D, H, L, P).

Fig 5. Light microscopy (A, B, E, F, I, J, M, N) and scanning electron microscopy (C, D, G, H, K, L, O, P) micrographs of extant Berlinia Clade pollen from Africa continued

A–D. *Englerodendron leptorrhachis* (from Cameroon, coll. Satabié, 980 [K001341298]). A. Polar views, high focus (left), optical cross-section (right). B. Equatorial views, high focus (left), optical cross-section (right). C. Equatorial view. D. Close-up showing aperture membrane and adjacent striate sculpture.

E–H. *Englerodendron mengei* (from DR Congo, coll. J Louis, 3666 [K000557102]). E. Polar views, high focus (left), optical cross-section (right). F. Equatorial views, high focus (left), optical cross-section (right). G. Equatorial view. H. Close-up showing aperture membrane and adjacent striate sculpture.

I–L. *Englerodendron usambarense* (from Tanzania, coll. Greenway, 1061 [K000555978]). I. Equatorial views, high focus (left), optical cross-section (right). J. Equatorial views, high focus (left), optical cross-section (right). K. Equatorial view. L. Close-up showing aperture membrane and adjacent striate sculpture. M–P. *Isoberlinia scheffleri* (from Tanzania, coll. Greenway, 3319 [K000557103]). M. Polar views, high focus (left), optical cross-section (right). N. Equatorial views, high focus (left), optical cross-section (right). O. Equatorial view. P. Close-up showing aperture membrane and adjacent striate sculpture. Scale bars – 10 µm (A–C, E–G, I–K, M–O), 2 µm (D, H, L, P).

Comments: The morphology (LM and SEM) of the fossil Mush MT pollen is similar to those of extant pollen produced by *Englerodendron*, *Isoberlinia*, and *Berlinia* (compared in Table 2). The Mush MT has a much broader size and P/E range and shows more variability in the general sculpture and in the sculpture of the aperture membrane than pollen from extant taxa. The reason for this is uncertain, but might be explained by one of the following: 1) the fossil pollen grains originate from the same biological taxon but reflect a much greater sampling source and originate from different anthers/flowers/individuals as compared to a single anther providing the extant comparison material. 2) the fossil pollen grains originate from more than a single biological taxon but their pollen overlap in morphological features and cannot be distinguished. 3) The fossil pollen grains originate from a single biological taxon and their morphological plasticity reflects an ancestral trait partly preserved in different extant descendants. Whatever the reason, it is clear that the Mush MT is most comparable to pollen from some extant *Englerodendron* and therefore supports a likely affiliation with the *Englerodendron mulugeta* sp. nov. fossil leaves.

DISCUSSION

Taxonomic, evolutionary, and biogeographic, implications

Englerodendron is a moderately sized genus in the Amherstieae (Fabaceae: Detarioideae) consisting of 18 extant species belonging within a monophyletic subclade within the Berlinia Clade, referred to as Berlinia Clade Subclade B, which consists of *Anthonotha*, *Berlinia*, *Englerodendron*, *Isoberlinia*, *Librevillea*, and *Oddoniodendron* [48, 49, 50, 51, 52]. *Englerodendron* was formerly considered monospecific, consisting of a submontane forest species *E. usambarense*, endemic to the West Usambara Mountains in the Eastern Arc Mountains of Tanzania [53, 54]. The genus, which is sister to *Anthonotha*, now includes a number of species formerly included in the latter genus, as well as species of the now synonymized *Isomacrolobium* and *Pseudomacrolobium* [48, 50].

Englerodendron sensu lato can now be characterized ecologically more broadly as a moist and wet forest genus (although *E. explicans* can also occur in savanna woodlands) that is mainly restricted to the Guineo-Congolian region along with the isolated Eastern Arc *Englerodendron usambarense* [53, 54] (Fig 6). Species can occur in a variety of forest types including primary rain forest, semi-deciduous moist forests, riverine forest, gallery forest, submontane forest, and even lagoon-margin forest habitat [48, 49, 10, 52]. While extant species of *Englerodendron* do not develop into monodominant forest formations today, morphological and ecological characteristics hypothesized to assist in the development of such formations are prevalent within the genus and subclade. These characteristics include large seed size and short dispersal capabilities, shade-tolerant seedlings, ectomycorrhizal associations, conspecific gregariousness, and observations of coppicing within the genus are noted [55, 49, 56, 2].

Fig 6. Distribution map and terrestrial ecoregion occurrence of extant *Englerodendron* (circles) and location of the fossil species, *Englerodendron mulugeta* (star)

The early Miocene *Englerodendron mulugeta* sp. nov. is the only known fossil occurrence of the genus, although pollen attributed to closely related genera (*Anthonotha*, *Berlinia*, and *Isoberlinia*) is

known from the late Paleocene and early Eocene of Nigeria and the late Oligocene - early Miocene of northern Kenya – indicating a long evolutionary history of the Berlinia Clade: Subclade B in Africa [57, 58, 37, 59]. Some fossil lamina impressions from the early Miocene Bugishu Series in Uganda also appear to be similar to *Berlinia*, but definitively assigning a generic identification to this material is not possible. These fossil records and the early Miocene (21.73 Ma) occurrence of *Englerodendron mulugeta* sp. nov. indicate that late Pliocene or early Pleistocene evolutionary divergence time estimates for the genus and its sister taxon, *Anthonotha*, by Estrella et al. [37] need to be re-assessed.

In the Afrotropics, the majority of moist and wet monodominant forests consist of detarioid legume species as their principal constituent [60, 6, 4, 61, 62, 63, 64] (Table 1). Temporary monodominant forest formations dominated by non-legume taxa can develop in pioneer seral stage vegetation (e.g., *Musanga* spp. - Urticaceae; [3, 5, 65, 66]. Within the moist and wet detarioid legume African monodominant forest formations, all ecologically dominant species that fulfill this role belong within the Amherstieae, the largest and most diverse tribe in the Detarioideae composed of about 50 genera and about 570 species [44, 67]. Nevertheless, Subclade B of the Berlinia Clade of the tribe is absent among modern forest species dominants [37]. The majority of species that currently constitute monodominant forests in Africa are found in Subclade A of the Berlinia Clade, which is composed of the 'bambijt' clade + (*Gilbertiodendron* + *Didelotia*) [37]. The sole other species is found in the mid-level position in the phylogeny of the Amherstieae (*Cynometra alexandri*; [37]. Many genera within the Berlinia Clade of Amherstieae possess characteristics (as noted above) that could provide an ecological pathway to becoming an ecological dominant in moist and wet Afrotropical forests given an opportunity. Within the other speciose tribe within the Detarioideae, the Detarieae, multiple genera and species can be found in tropical African forest formations, but only one taxon, *Gilletiodendron glandulosum*, is known to dominate a forest community [68]. In this case, the community represents a relictual Sudanian-type of

dry forest and falls outside the purview of this study [68]. Three additional Detarieae genera, *Colophospermum*, *Copaifera*, and *Guibourtia*, develop monotypic stands in African communities [10].

Paleoecology and Evidence for a Monodominant Forest

Extensive analyses of plant macro- and microfossils, and isotope and organic geochemistry from the Mush Valley lacustrine deposits provided a view of the surrounding community and the lake itself, which accumulated fine sediments and organic matter over some 50 – 60 ka [22, 23]. The following results of those studies together indicated the ancient terrestrial community was a forest:

(1) The average leaf carbon isotope discrimination relative to atmospheric CO_2 ($\Delta^{13}C$) derived from organically preserved leaf compressions is 23.4 ‰, equal to that of modern tropical rainforests [22, 69].

(2) The reconstructed mean annual precipitation among Levels A - F, ranges from $1523\frac{+199}{-125}$ to $1638\,\frac{+215}{-125}$ mm/yr, comparable to values supportive of forest vegetation [22].

(3) Productive phytolith samples collected from 2-7 m above and below the macrofossil deposits are dominated by non-grass forest forms [23].

(4) The $\delta^{13}C$ values of terrestrially sourced C_{29} and C_{31} *n*-alkanes (angiosperm dicots) from bulk organic samples among Levels A-F are −35 to −32‰, indicative of purely C_3 vegetation [23].

(5) Terrestrial plant macrofossil identifications to this point include species limited to forest communities today: *Newtonia mushensis* [24], *Tacca umerii* [24, 25, 22, 23, 27], and now *Englerodendron mulugeta sp. nov*.

(6) Terrestrial fossil pollen analyzed using combined LM and SEM, and even TEM, for single-grains also include taxa occurring in Africa’s forests: *Aristogeitonia* / *Mischodon* / *Oldfieldia* / *Voatamalo* clade [70; fig. 19], *Sclerosperma* [71; fig. 3], and *Hagenia* [72; figs 6-8] .

Although Currano et al. [23, 27] concluded the Mush forest was a mixed-moist, semi-evergreen closed-canopy forest similar to those found today in West, Central, and eastern Africa [e.g., 74], the long-term abundance and consistent dominance of *Englerodendron mulugeta* sp. nov. leaflets at all lake levels sampled, and its placement in the Berlinia Clade, tribe Amherstieae, among which are species most commonly forming monodominant wet forests today, prompted our reconsideration of Mush forest structure. The consistent and overwhelming abundance of *E. mulugeta* leaflets among the fossil assemblages is a compelling aspect of the collections, and led to the hypothesis of a monodominant forest at Mush by Bush et al. [22]. This taxon comprises 53% of all classified leaves (1289 of 2427; leaflets are counted as leaves) and as much as 66% of specimens among the six stratigraphic levels ([23], and Table 3). Taphonomic processes, documented by studies of modern leaf fall, transport, litter accumulation, and preservation indicate that canopy leaves are more common than those from the understory in forest litter and local sedimentary deposits, and that leaves travel short distances before deposition, especially if thick and heavy; compound-leaved and deciduous trees can have an outsized impact on the composition of local (e.g., lakeside) deposits [74, 75, 76, 77]. Thus, to understand results of these processes as they influence leaf accumulations from monodominant vs. mixed forests, litter studies of comparable modern tropical wet forests must be consulted. Analyses of modern leaf litter in African forests are rare, but Peh et al. [78] compared the litterfall, its rate of decomposition, and representation of the dominant taxon in litter samples in single-dominant vs. mixed forest plots in Cameroon (Table 3). Monodominant forests of *Gilbertiodendron dewevrei* (a Berlinia Clade legume) produced leaf litter whose dry weight was 52% - 92% (average of 73% ± 23%) *G. dewevrei* leaves [78]. Peh et al. [78] did not measure the proportion of leaf litter belonging to the most abundant species present in each mixed forested sampled, but the proportion of leaves belonging to the most abundant taxon in modern litter censuses from diverse Neotropical forests ranges from 12.8 to 31.4% (Table 3).

Table 3. Comparison of diversity and dominance measures among fossil and modern localities [27, 78, 63]. Numbers shaded in blue highlight collections where the dominant taxon comprises ≥ 50%. Numbers shaded in green highlight leaf collections with Hill values ≥ 4.0.

Locality	Age	Data Type	Number of Leaves	Species Richness at 300 Leaves	Hill # q=2	Percent Dominant Taxon
Guang	27.1 Ma	Fossil leaves	433	34.8	**4.57**	43.7
Bull's Bellow	27.1 Ma	Fossil leaves	606	27.9	**6.38**	33.8
Mush A	21.7 Ma	Fossil leaves	349	25	**6.76**	30.9
Mush B	21.7 Ma	Fossil leaves	400	28.8	3.07	**55.8**
Mush C	21.7 Ma	Fossil leaves	95	NA	3.97	**49.5**
Mush D	21.7 Ma	Fossil leaves	532	30.9	3.50	**50.9**
Mush E	21.7 Ma	Fossil leaves	510	27.0	3.12	**55.7**
Mush F	21.7 Ma	Fossil leaves	541	20.4	2.16	**65.8**
BCI	Modern	Leaf litter	672	56.4	**15.9**	18.0
Yasuni	Modern	Leaf litter	951	50.7	**7.9**	31.4
Rio Negro	Modern	Leaf litter	932	70.1	**22.1**	12.8
Peh mono1	Modern	Leaf litter				**52**
Peh mono2	Modern	Leaf litter				**73**
Peh mono3	Modern	Leaf litter				**92**
Kearsley monodominant	Modern	Tree plots, dbh data			8.4 ± 1.2	65.3
Kearsley mixed	Modern	Tree plots, dbh data			21.2 ± 3.6	16.7

Measures of diversity are also lower for monodominant forests than mixed forests because of the presence of several species in low numbers (Table 3) [78,18, 63]. In assessing diversity among the Mush leaf assemblages we chose to examine both rarefied species richness and Shannon diversity (Hill number, q=2), which are commonly used in neoecological studies because values are more readily comparable among different communities [79]. A Hill number is the "effective number of species," which is defined as the number of equally abundant species needed to produce the given value of a diversity index [79, 23]. Rarefied richness gives equal weight to rare and common taxa, whereas Shannon diversity emphasizes abundant taxa and can be thought of as the number of equally abundant common species. Table 3 provides diversity comparisons among Mush assemblages, modern leaf assemblages, and Kearsley et al.'s [63] study from monodominant and mixed forests in the Democratic Republic of Congo.

Fossil leaf collections from an Oligocene Ethiopian site (Chilga) to the north of Mush, and that have diversity measures demonstrating a richer and more even frequency distribution of taxa, provide a fossil point of comparison. The two Chilga localities listed in Table 3, Guang and Bull's Bellow, are contemporaneous, but located about 1.5 km apart. They share no taxa, and represent diverse, mixed forests at different stages of succession [80]. The most dominant taxon among leaves collected at each site comprises 44% and 34% of 433 and 606 leaves, respectively [27]. The Hill numbers are in line with, albeit lower than, leaf litter collections from a very diverse modern mixed forest at Yasuni, Ecuador [27].

As noted, *Englerodendron mulugeta* sp. nov. is the most abundant taxon at each stratigraphic level collected at Mush. Level A, near the base of the section, contains only 31% *E*. *mulugeta* sp. nov. leaflets and has a higher Shannon diversity than either Chilga site (Table 3). Thus, we interpret the leaf assemblage from Level A as having come from a mixed rather than monodominant forest. Significant volcanic activity is associated with the formation of the paleolake, and it is possible that the forest at the time of Level A was on its way to monodominance. By the time of Level B, Shannon diversity drops significantly and *E*. *mulugeta* sp. nov. leaflets comprise 55.8% of the assemblage [23], within the range that Peh et al. [78] observed in *G*. *dewevrei* monodominant forests. Shannon diversity remains low, and the proportion of *E*. *mulugeta* sp. nov. leaflets high, for the remainder of the Mush stratigraphic levels. Perhaps the most compelling aspect of the Mush assemblages in support of monodominance is the consistent abundance of *E*. *mulugeta* sp. nov. over the 50 – 60 kyrs sampled [23]. This fact argues against the presence of a single tree overarching or close to the shore of the ancient lake. In addition, although compound-leaved legume taxa are generally the most abundant overall, only *E. mulugeta* sp. nov. dominates the assemblages at each stratigraphic level.

The presence of a long-lived, prehistoric monodominant forest in northwestern Ethiopia raises questions about the role of such forests in the evolutionary history and the paleoecological processes of

African forest vegetation during the early Miocene. Currently, no other Cenozoic tropical monodominant forests have been identified from before the Holocene. However, the likelihood that this assemblage represents the sole monodominant forest existing on the continent during the early Miocene is also relatively remote. As noted above, a number of monodominant forest types, differing in the dominant species constituent and type (persistence, seral stage, edaphically maintained, etc.), occur on the continent today and the only shared characteristic among them is that a single arborescent species makes up more than 50% of the canopy constituents of such forests [3, 81, 82]. Thus, monodominant forests do not harbor the magnitude of plant biodiversity and structural complexity as moist and wet mixed tropical forest formations, and consequently have bottom-up food web impacts for example, the diversity and intensity of frugivory and herbivory, as well as vertebrate biodiversity and biomass [83, 84, 85, 86]. In most modern studies, observations of primate abundances and species-richness, elephant densities and activities, and rodent species-richness are reduced in *Gilbertiodendron dewevrei*-dominated monodominant forest formations in comparison to adjacent mixed and secondary forest formations [84, 85, 87, 88]. Typically, mammals prefer secondary and mixed forests due to the abundance of food resources in such forest types in comparison to primary or late secondary monodominant forests [83, 84, 89]. Correspondingly, the reduction of faunal elements in some forested habitats may actually increase the likelihood of monodominant forests developing or expanding [90]. There is some evidence that reduced elephant activity in western Uganda may assist in the development of *Cynometra alexandri* monodominant forests [91].

The presence of monodominant forests also creates additional vegetation heterogeneity within the tropical African sphere and how this affected forest faunal distributions and evolution is not known, but provides an additional aspect to consider – particularly in understanding dynamic changes in tropical forest distribution during the Miocene and how forest extent and composition may have affected particular mammal groups such as primates. The origin, temporal record, and spatial scale of Cenozoic

monodominant forests in Africa are unknown, as Mush remains but a single datum point; however, this gap may be filled potentially by additional investigations of well-sampled, well-preserved plant macrofossil assemblages.

ACKNOWLEDGMENTS

We thank the Authority for Research and Conservation of Cultural Heritage for permission to conduct research in the Mush Valley and the director and staff of the National Museum of Ethiopia for facilitating our research. We are grateful to the people of Upper and Lower Mush for their hospitality, and to N. Tabor, M. Clemens, M. Feseha, L. Jacobs, J. Noret, D. Danehy, and T. Tesfamichael for contributions to field work. In particular we are grateful to M. Feseha for his role in bringing the Mush Valley locality to our attention. We are also grateful to D. Erwin, UC Museum of Paleontology, for providing images of fossils from the Bugishu Series in Uganda and T. Rehman, Botanical Research Institute of Texas, for providing herbarium material for us to examine.

REFERENCES

1. Gentry AH. Tree species richness of upper Amazonian forests. PNAS. 1988;86:156 – 159.

2. ter Steege H, Henkel TW, Helal N, Marimon BS, Marimon BH Jr, Huth A. et al. 2019. Rarity of monodominance in hyperdiverse Amazonian forests. Scientific Reports. 2019;9:13822. https://doi.org/10.1038/s41598-019-50323-9.

3. Connell JH, Lowman MD..Low-diversity tropical rain forests: some possible mechanisms for their existence. The American Naturalist. 1989;134:88 – 119.

4. Torti SD, Coley PD, Kursar TA.. Causes and consequences of monodominace in tropical lowland forests. The American Naturalist. 2001;157:141 – 153.

5. Richards PW. 1996. The tropical rain forest, 2nd edition. Cambridge (UK):Cambridge University Press; 1996.

6. Hart TB, Hart JA, Murphy PG. Monodominant and species-rich forests of the humid tropics: causes for their co-occurrence. The American Naturalist. 1989;133:613 – 633.

7. Nascimento MT, Proctor J. Soil and plant changes across a monodominant rain forest boundary on Maracá Island, Roraima, Brazil. Global Ecology and Biogeography Letters. 1997;1:387-395.

8. Mackinder BA, Saslis-Lagoudakis H, Wieringa JJ, Devey D, Forest F, Bruneau A. The tropical African legume Scorodophloeus clade includes two undescribed *Hymenostegia* segregate genera and *Micklethwaitia*, a rare, monospecific genus from Mozambique. South African Journal of Botany 2013;89:156 – 163.

9. Estrella M de la, Wieringa JJ, Mackinder B, van der Burgt X, Devesa JA, Brounot A. Phylogenetic analysis of the African genus *Gilbertiodendron* J. Léonard and related genera (Leguminosae-Caesalpinioideae-Detarieae). Intl J Plant Sci. 2014;175:975-985.

10. Lebrun J-P, Stork AL. Tropical African flowering plants: ecology and distribution, Volume 3: Mimosaceae – Fabaceae (incl. *Derris*). Conservatoire et Jardin botaniques de la Ville de Genève, Oceanography 2008;16:349 – 356.

11. Heckenhauer J, Samuel R, Ashton PS, Turner B, Barfuss MHJ, Jang T-S, et al. Phylogenetic analyses of plastid DNA suggest a different interpretation of morphological evolution than those used as the basis for previous classifications of Dipterocarpaceae (Malvales). Bot. J. Linn. Soc. 2017;185:1 – 26.

12. Ashton PS, Morley RJ, Heckenhauer K, Prasad V. The magnificent dipterocarps: précis for an epitaph? Kew Bulletin 2021.https://doi.org/10.1007/s12225-021-09934-7.

13. Couvreur TLP, Dauby G, Blach-Overgaard A, Deblauwe V, Dessein S, Droissart V, et al. Tectonics, climate and diversification of the tropical African terrestrial flora and fauna. Biological Reviews. 2021;96:16 – 51.

14. Makana J-R, Ewango CN, McMahon SM, Thomas SC, Hart TB, Condit R. Demography and biomass change in monodominant and mixed old-growth forest of the Congo. J Trop Ecol. 2011;27:447 – 461.

15. Masens da-Musa YB, Ngbolua K, Masens M, Tambu TM, Gédéon NB. Phytoecological study of Nzundu massif forest of Imbongo city, Kwilu Province, Democratic Republic of the Congo. Trop Plant Res. 2017;4:363 – 375.

16. Pfadenhauer JS, Klötzli F. Global vegetation: fundamentals, ecology and distribution. Berlin (Ger): Springer; 2020.

17. Peh KS-H, Lewis SL, Lloyd J. Mechanisms of monodominance in diverse tropical tree-dominated systems. J Ecol. 2011;99:891 – 898.

18. Djuikouo MNK, Peh KS-H, Nguembou CK, Doucet JL, Lewis SL, Bonaventure S. Stand structure and species co-occurrence in mixed and monodominant Central African tropical forests. J Trop Ecol. 2014; 30:447–455. https://doi.org/10.1017/S0266467414000352.

19. Gomes AL, Revermann R, Meller P, Gonçalves FMP, Aidar MPM, Lages F, et al. Functional traits and symbiotic associations of geoxyles and trees explain the dominance of detarioid legumes in miombo ecosystems. New Phytologist. 2021;230:510 – 520.

20. Crawley M. Palaeocene wood from the Republic of Mali. Bulletin of the British Museum of Natural History (Geology). 1988;44: 3 – 14.

21. Herrera F, Carvalho MR, Wing SL, Jaramillo C, Herendeen PS. Middle to late Paleocene leguminosae fruits and leaves from Colombia. Australian Systematic Botany. 2019;32:385 – 408.

22. Bush RT, Wallace J, Currano ED, Jacobs BF, McInerney FA, Dunn RE, et al. Cell anatomy and leaf $\delta^{13}C$ as proxies for shading and canopy structure in a Miocene forest from Ethiopia. Palaeogeography, Palaeoclimatology, Palaeoecology. 2017;485:593 – 604.

23. Currano ED, Jacobs BF, Bush RT, Novello A, Feseha M, Grímsson, et al. Ecological dynamic equilibrium in an early Miocene (21.73 Ma) forest, Ethiopia. Palaeogeogrpahy, Palaeoclimatology, Palaeoecology. 2020; https://doi.org/10.1016/j.palaeo.2019.109425

24. Pan AD, Currano ED, Jacobs BF, Feseha M, Tabor N, Herendeen PS. Fossil *Newtonia* (Fabaceae: Mimoseae) seeds from the early Miocene (22 – 21 Ma) Mush Valley in Ethiopia. International Journal of Plant Sciences. 2012;173:290 – 296.

25. Pan AD, Jacobs BF, Currano ED. Dioscoreaceae fossils from the late Oligocene and early Miocene of Ethiopia. Bot J Lin Soc. 2014;175:17 – 28.

26. Engel MS, Currano ED, Jacobs BF. The first mastotermitid termite from Africa (Isoptera: Mastotermitidae): a new species of *Mastotermes* from the early Miocene of Ethiopia. Journal of Paleontology. 2015;89:1038–1042.

27. Currano ED, Jacobs BF, Pan AD. Is Africa really an "odd man out"? Evidence for diversity decline across the Oligocene-Miocene boundary. International Journal of Plant Sciences. 2021;https://doi.org/10.1086/714308.

28. Tesfamichael T, Jacobs BF, Tabor NJ, Michel L, Currano ED, Feseha M, et al. Settling the issue of "decoupling" between atmospheric carbon dioxide and global temperature: $[CO_2]_{atm}$ reconstructions across the warming Paleogene-Neogene divide. Geology. 2017;45:999 – 1002.

29. Herendeen PS, Dilcher DL. Fossil mimosoid legumes from the Eocene and Oligocene of southeastern North America. Review of Palaeobotany and Palynology. 1990;62:339-61.

30. Grímsson F, Denk T, Zetter R. Pollen, fruits, and leaves of *Tetracentron* (Trochodendraceae) from the Cainozoic of Iceland and western North America and their palaeobiogeographic implications. Grana. 2008;47:1–14. https://doi.org/10.1080/00173 13070 1873081

31. Zetter R. Methodik und Bedeutung einer routinemäßig kombinierten lichtmikroskopischen und rasterelektonenmikroskopischen Untersuchung fossiler Mikrofloren. Courier Forschungsinstitut Senckenberg. 1989;109:41–50.

32. Halbritter H, Ulrich S, Grímsson F, Weber M, Zetter R, Hesse M, et al. Illustrated pollen terminology, 2nd ed. Springer. 2018; https://doi.org/10.1007/978-3-319-71365-6

33. Grímsson F, Grimm GW, Zetter R. Tiny pollen grains: First evidence of Saururaceae from the Late Cretaceous of western North America. *PeerJ* 5: e3434. https://doi.org/10.7717/peerj.3434

34. Grímsson F, Grimm GW, ZetterR. 2018. Evolution of pollen morphology in Loranthaceae. Grana 2017;57:16–116. https://doi.org/10.1080/00173 134.2016.1261939

35. Punt W, Hoen PP, Blackmore S, Nilsson S, Le Thomas A. Glossary of pollen and spore terminology. Rev Pal Pal. 2007;143:1–81. https://doi.org/10.1016/j.revpalbo.2006.06.008

36. Dilcher, D.L. Approaches to identification of angiosperm leaf remains. Bot. Rev. 1974;40:1–157.

37. Estrella M de la, Cervantes S, Janssens SB, Forest F, Hardy OJ, Ojeda DI. The impact of rainforest area reduction in the Guineo-Congolian region on the tempo of diversification and habitat shifts in the Berlinia clade (Leguminosae). J Biogeogr. 2020;47:2728 – 2740.

38. Hawthorne W, Jongkind C. Woody plants of Western African Forests: a guide to the forest trees, shrubs, and lianes from Senegal to Ghana. Kew (London): Kew Publishing: Kew; 2006.

39. Estrella M de la, Devesa JA, Wieringa JJ. A morphological re-evaluation of the taxonomic status of the genus *Pellegriniodendron* (Harms) J. Léonard (Leguminosae-Caesalpinioideae-Detarieae) and its inclusion in *Gilbertiodendron* J. Léonard. S Afr J Bot 2012;78:257 – 265.

40. Ngok Banak L, Breteler FJ. Novitates Gabonenses 50. The genus *Oddoniodendron* (Leguminosae, Caesalpinioideae) from Lower Guinea: a taxonomic revision with description of two new species from Gabon. Adansonia. 2004;26:241 – 250.

41. Burgt XM van der, Eyakwe M, Motoh J. *Gilbertiodendron newberyi* (Leguminosae: Caesalpinioideae), a new tree species from Korup National Park, Cameroon. Kew Bulletin. 2012;67:51 – 57.

42. Burgt XM van der, Mackinder BA, Wieringa JJ, Estrella M de la. The *Gilbertiodendron ogoouense* species complex (Leguminosae: Caesalpinioideae), Central Africa. Kew Bull. 2015;70:29. https://doi.org/10.1007/s12225-015-9579-4

43. Wieringa JJ. *Monopetalanthus* Exit.: a Systematic Study of *Aphanocalyx, Bikinia, Icuria, Michelsonia* and *Tetraberlinia* (Leguminosae, Casalpinioideae [dissertation]. Wageningen University and Research ProQuest Dissertations Publishing, 1999. 28233563.

44. Mackinder B. Tribe Detarieae. In: Lewis G, Schrire B, Mackinder B, Lock M, eds. Legumes of the world. Kew: Royal Botanic Gardens. 2005. p. 69–109.

45. Radosavljevic A, Mackinder BA, Herendeen PS. Phylogeny of the detarioid legume genera *Cynometra* and *Maniltoa*. Sys Bot. 2017;42:1 – 10.

46. Radosavljevic A. The rise of *Cynometra* (Leguminosae) and the fall of *Maniltoa*: a generic re-circumscription and the addition of 4 new species. PhytoKeys. 2019;127:1 – 37.

47. Obembe OA. Structural variation of stomata in some dicotyledonous trees. Global J Biol, Agr & Hlth Sci. 2015;4:173 – 181.

48. Breteler FJ. 2006. Novitates Gabonenses 56. Two *Anthonomtha* species from Gabon transferred to *Englerodendron* (Fabaceae, Caesalpinioideae). Adans 2006;28:105 – 111.

49. Breteler FJ. Revision of the African genus *Isomacrolobium* (Leguminosae: Caesalpinioideae). Plant Ecol Evol. 2011;144:64 – 81.

50. Estrella M de la, Wieringa JJ, Breteler FJ, Ojeda DI. Re-evaluation of the genus *Englerodendron* (Leguminosae – Detarioideae), including *Isomacrolobium* and *Pseudomacrolobium*. *Australian* Syst Bot. 2019;32:564 – 571.

51. Ojeda DI, Koenen E, Cervantes S, Estrella M de la, Banguera-Hinestroza E, Janssens SB, et al. Phylogenomic analyses reveal an exceptionally high number of evolutionary shifts in a florally diverse clade of African legumes. Mol Phylogenet Evol. 2019;137:156 – 167.

52. Jongkind CCH, Breteler FJ. *Englerodendron libassum* (Leguminosae-Detarioideae-Amherstieae), a new critically endangered tree species from coastal Liberia. Plant Ecol Evol. 2020;153:487 – 491.

53. Schulman, L.; Junikka, L.; Mndowla, A.; Rajabu, I. Trees of Amani Nature Reserve, NE Tanzania. Ministry of Natural Resources and Tourism. Helsinki: Helsinki University Printing House; 1998.

54. Lovett JC, Ruffo CK, Gereau RE, Taplin JRD. Field guide to the moist forest trees of Tanzania. York (UK): York Publishing Services; 2006.

55. Burgt XM van der, Eyakwe MB, Newbery DM. *Englerodendron korupense* (Fabaceae, Caesalpinioideae), a new tree species from Korup National Park, Cameroon. Adansonia. 2007;29:59 – 65.

56. Tedersoo L, Brundrett MC. Evolution of ectomycorrhizal symbiosis in plants. Ecol Stud. 2017;230:407 – 467.

57. Morley RJ. Origin and evolution of tropical rain forests. Chichester (UK): Wiley; 2000.

58. Vincens A, Tiercelin J-J, Buchet G. New Oligocene – early Miocene microflora from the southwestern Turkana Basin: palaeoenvironmental implications in the northern Kenya Rift. Palaeogeogr, Palaeoclim, Palaeoecol. 2006;239:470 – 486.

59. Romero IC, Kong S, Fowlkes CC, Jaramillo C, Urban MA, Oboh-Ikuenobe F, et al. Improving the taxonomy of fossil pollen using convolutional neural networks and superresolution microscopy. PNAS. 2020;117:28496 – 28505. https://doi.org/10.1073/pnas.2007324117.

60. Eggeling WJ. Observations on the ecology of the Budongo Rain Forest, Uganda. J Ecol. 1947;34:20 – 87.

61. Poorter L, Bongers F, Kouamé FNG, Hawthorne WD. Biodiversity of West African forests: an ecological atlas of wood plant species. CABI:2004. e-book: https://cabidigitallibrary.org/doi/book/10.1079/9780851997346.0000

62. Newbery DM, Burgt XM van der, Worbes M, Chuyong GB. Transient dominance in a central African rain forests. Ecol Monogr. 2013;83:339 – 382.

63. Kearsley E, Verbeeck H, Hufkens K, Van de Perre F, Doetterl S, Baert G, et al. Functional community structure of African monodominant *Gilbertiodendron dewevrei* forest influenced by local environmental filtering. Ecol Evol. 2016;7:295 – 304. https://doi.org/10.1002/ece3.2589 .

64. Burgt XM van der. Co-dominant Detarioideae (Leguminosae) tree species in the rain forests of Korup National Park [dissertation]. Oxford (UK): Oxford Brookes University; 2018.

65. Furuichi T, Hashimoto C, Tashiro Y. Fruit availability and habitat use by chimpanzees in the Kalinzu Forest, Uganda: examination of fallback foods. Int J Primatol. 2001;22:929 – 945.

66. Pangou SV, Maury-Lechon G, Moutanda A. Monodominant forests of *Aucoumea klaineana* and *Terminalia superba* in the Chaillu Forest (SW Congo, Africa). Pol Bot J. 2003;48:145 – 162.

67. Estrella M de la, Forest F, Wieringa JJ, Fougère-Danezan M, Bruneau A. Insights on the evolutionary origin of Detarioideae, a clade of ecologically dominant tropical African trees. New Phytol. 2017;214: 1722-1735. https://doi.org/10.1111/nph.14523

68. Duvall CS. Habitat, conservation, and use of *Gilletiodendron glandulosum* (Fabaceae, Caesalpinioideae) in southwestern Mali. Syst Geogr Plants. 2001;71:699 – 737.

69. Diefendorf, A.F., Mueller, K.E., Wing, S.L., Koch, P.L., Freeman, K.H., Global patterns in leaf C-13 discrimination and implications for studies of past and future climate. PNAS. 2010;107:5738‐5743.

70. Grímsson FG, Graham SA, Coiro M, Jacobs BF, Xafis A, Neumann FH, et al. Origin and divergence of Afro-Indian Picrodendraceae: linking pollen morphology, dispersal modes, fossil records, molecular dating and paleogeography. Grana 2019;58:227 – 275.

71. Ulrich S, Grímsson F. The single-grain method: adding TEM to the equation. Grana 2020;59:44 – 57.

72. Grímsson FG, Ulrich S, Coiro M, Graham SA, Jacobs BF, Currano ED, et al. *Hagenia* from the early Miocene of Ethiopia: Evidence for possible niche evolution. *Ecology and Evolution* 2021;11:5164 – 5186.

73. White, F. The Vegetation of Africa, a Descriptive Memoir to Accompany the UNESCO/AETFAT/UNSO Vegetation Map of Africa (3 Plates, Northwestern Africa, Northeastern Africa, and Southern Africa, 1: 5,000,000). Paris (France): Unesco; 1983.

74. Ferguson, D.K. The origin of leaf-assemblages-new light on an old problem. Rev Pal Palynol. 1985;46:117-88.

75. Spicer RA. The formation and interpretation of plant fossil assemblages. Adv Bot Res. 1989;16:96-191.

76. Burnham R, Relationships between standing vegetation and leaf litter in a paratropical forest: implications for paleobotany. Rev Pal Palynol. 1989;58:5-32.

77. Greenwood DR. The taphonomy of plant macrofossils. In: Donovan, SK, editor. The Processes of Fossilization. New York: Columbia University Press; 1991.p. 141-169.

78. Peh KS-H, Sonké B, Séné O, Djuikouo M-NK, Nguembou CK, Taedoumg H, et al. Mixed-forest species establishment in a monodominant forest in Central Africa: implications for tropical forest invasibility. PLOS One. 2014;9:e97585. https://doi.org/10.1371/journal.pone.0097585.g001.

79. Jost L. Partitioning diversity into independent alpha and beta components. Ecology. 2007;88:2427–2439.

80. Currano, E.D., Jacobs, B.F., Pan, A.D., Tabor, N.J., Inferring ecological disturbance in the fossil record: a case study from the late Oligocene of Ethiopia. Palaeogeogr. Palaeoclimatol. Palaeoecol. 2011;309: 242–252.

81. Torti SD, Coley PD. Tropical monodominance: a preliminary test of the ectomycorrhizal hypothesis. Biotropica. 1999;31:220 – 228.

82. McGuire KL. Common ectomycorrhizal networks may maintain monodominance in a tropical rain forest. Ecol. 2007;88:567 – 574.

83. Thomas SC. Population densities and patterns of habitat use among anthropoid primates of the Ituri Forest, Zaïre. Biotropica. 1991;23:68 – 83.

84. Gubista KR. Small mammals of the Ituri forest, Zaire: diversity and abundance in ecologically distinct habitats. J Mammal. 1999;80:252 – 262.

85. Morgan D, Sanz C, Ononponga JR, Strindberg S. Ape abundance and habitat use in the Goualougo Triangle, Republic of Congo. Int J Primatol. 2006;27:147 – 179.

86. Mockrin MH, Rockwell RF, Redford KH, Keuler NS. Effects of landscape features on the distribution and sustainability of ungulate hunting in northern Congo. Cons Biol. 2011;25:514 – 525.

87. Devos C, Sanz C, Morgan D, Onononga J-R, Laporte N, Huynen M-C. Comparing ape densities and habitats in northern Congo: surveys of sympatric gorillas and chimpanzees in Odzala and Ndoki Regions. Amer J Primatol. 2008;70:1 – 13.

88. Breuer T, Breuer-Ndoundou Hockemba M, Strinderg S. Small-scale dung survey reveals high forest elephant density and preference for mixed species forest in an intact protected area. Biodivers Conserv. 2021: http://doi.org/10.1007/s10531-021-02214-7.

89. Melletti M, Mirabile M, Penteriani V, Boitani L. Habitat use by forest mammals in Dzanga-Ndoki National Park, Central African Republic. Afr J Ecol. 2009;47:797 – 800.

90. Kurten EL, Carson WP. Do ground-dwelling vertebrates promote diversity in a Neotropical forest? Results from a long-term exclosure experiment. BioSci. 2015;65:862 – 870.

91. Sheil D, Salim A. Forest tree persistence, elephants, and stem scars. Biotropica. 2004;36:505 – 521.

92. Swaine MD, Hall JB. The monospecific tropical forest of Ghanaian endemic tree *Talbotiella gentii*. In: Synge H, editor. The biological aspects of rare plant conservation. London (UK): J. Wiley; 1981. p. 355 – 363.

93. Voorhoeve AG. Some notes on the tropical rainforest of the Yoma-Gola National Forest near Bomi Hills, Liberia. Commonwealth For Rev. 1964;43:17 – 24.

94. van Rooyen M, van Rooyen N, Orban B, Nsongola G, Miabangana ES, Gaugris J. Floristic composition, diversity and structure of the forest communities in the Kouilou Département, Republic of Congo. Trop Ecol. 2016;57:805 – 824.

95. Nik Norafida NA, Nizam MS, Wan Juliana WA, Faezah P. Species richness and floristic variation of tree communities in *Dryobalanops aromatica* Gaertn. f. dominated forests of Peninsular Malaysia. Malayan Nature J. 2018;70:323 – 331.

96. Appanah S, Turnbull JM. A review of dipterocarps: taxonomy, ecology and silviculture. Bogor (Indonesia): Center for International Forestry Research; 1998.

97. Yamada I. Tropical rain forests of Southeast Asia: a forest ecologist's view (Monographs of the Center for Southeast Asian Studies, Kyoto University). Manoa: University of Hawaii Press; 1997.

98. Kondo T, Otani T, Lee SL, Tani N. Pollination system of *Shorea curtisii*, a dominant species in hill dipterocarp forests. J Trop For Sci. 2016;28:318 – 323.

99. Haryati JR, Azizah IN, Arisoesilaningsih E. *Eusideroxylon zwageri* (Ulin) as key species in two zones of Sangkima Rain Forest, Kutai National Park, East Kalimantan. J Trop Life Sci. 2011;11: 47 – 50.

100. Gamble JS. Gutta Percha trees of the Malay Peninsula. Bull. Misc. Inform. Kew: Royal Botanic Gardens, Kew;1907: 109 – 121.

101. Anbarashan M, Parthasarathy N. Tree diversity of tropical dry evergreen forests dominated by single or mixed species on the Coromandel coast of India. Trop Ecol. 2013;54:179 – 190.

102. Rahman MM, Nishat A, Rahman GMM, Ruprecht H, Vacik H. Analysis of spatial diversity of sal (Shorea robusta Gaertn. F) forests using neighbourhood-based measurements. Comm Ecol. 2008;9:193 - 199.

103. Lamotte S, Gajaseni J and Malaisse F. Structure diversity in three forest types of north-eastern Thailand (Sakaerat Reserve, Pak Tong Chai). Biotechnology, Agronomy and Society and Environment. 1998;2:192–202.

104. Bradford M, Murphy HT. The importance of large-diameter trees in the wet tropical rainforests of Australia. PLoS One. 2019;14(5): e028377. https://doi.org/10.1371/journal.pone.0208377

105. Martijena NE, Bullock SH. Monospecific dominance of a tropical deciduous forest in México. J Biogeogr. 1994;21:63 – 74.

106. Boucher DH. Seed predation by mammals and forest dominance by *Quercus oleoides*, a tropical lowland oak. Oecologia. 1981;49:409 - 414.

107. Craig RJ. Ecological characteristics of a native limestone forest on Saipan, Mariana Islands. Micronesica. 1992;25:85 - 97.

108. Mueller-Dombois D, Jacobi JD, Boehmer HJ.. `Ohi`a lehua rainforest: born among Hawaiian volcanoes, Evolved in Isolation: the story of a dynamic ecosystem with relevance to forests worldwide. Manoa (HI): Friends of the Joseph Rock Herbarium; 2013.

109. Anderson AB. Use and management of native forests dominated by Açaí palm (*Euterpe oleracea* Mart.) in the Amazon estuary. Adv Econ Bot. 1988;6:144 - 154.

110. Ragsac AC, Farias-Singer R, Freitas LB, Lohmann LG, Olmstead RG. Phylogeny of the Neotropical tribe Jacarandeae (Bignoniaceae). Am J Bot. 2020;106:1589 - 1601.

111. Degagne RS, Henkel TW, Steinberg SJ, Fox III L. Identifying *Dicymbe corymbosa* monodominant forests in Guyana using satellite imagery. Biotropica. 2009;41:7 – 15.

112. Nascimento MT, Barbosa RI, Dexter KG, de Castilho CV, da Silva Carvalho C. Is the *Peltogyne gracilipes* monodominant forest characterized by distinct soils? Acta Oecologica. 2017;85:104 – 107.

113. Condit R, Hubbell SP, Foster RB. Mortality and growth of a commercial hardwood 'el cativo', *Prioria copaifera*, in Panama. *Forest Ecology and Management* 1993;62:107 – 122.

114. Fonty E, Molino J-F, Prévost M-F, Sabatier D. A new case of neotropical monodominant forest: *Spirotropis longifolia* (Leguminosae-Papilionoideae) in French Guiana. J Trop Ecol. 2011;27:641 – 644.

115. Dantas AR, Guedes MC, de Cruz Vasconcelos C, Isacksson JGL, Pastana DNB, Lira-Guedes AC, et al. Morphology, germination, and geographic distribution of Pentaclethra macroloba (Fabaceae): a hyperdominant Amazonian tree. Rev Biol Trop. 2021;69:181 – 196.

116. Barnett AA, Boye SA, Kinap NM, dos Santos-Barnett TC, Camilo TT, Parolin P, et al. Buds, bugs and bienniality: the floral biology of *Eschweilera tenuifolia* (O. Berg) Miers in a black-water flooded forest, central Amazonia. Forest. 2020;11:1 - 28.

S1. Herbarium specimens examined

S2. Herbarium specimens sampled for micro-morphological (leaf cuticle) and palynological analysis

S3. Table of comparing African Amherstieae (Legmuminosae, Detarioideae) leaf and leaflet characteristics

S4. Abaxial and adaxial leaf cuticle images of Berlinia Clade (Leguminosae, Detarioideae, Amherstieae) taxa

Fig 1

Click here to access/download;Figure;Fig 1.TIF

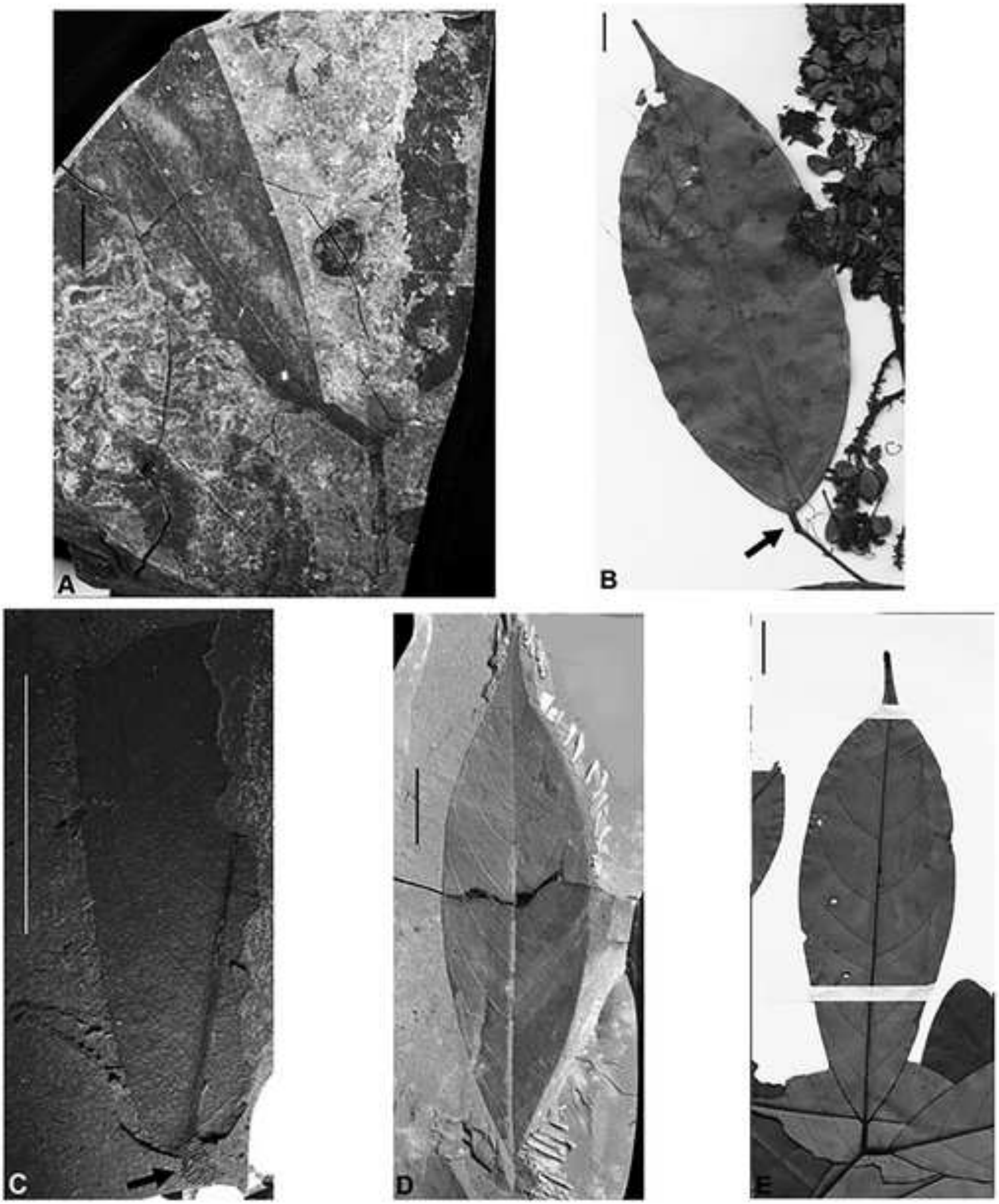

Click here to access/download;Figure;Fig 2.tif

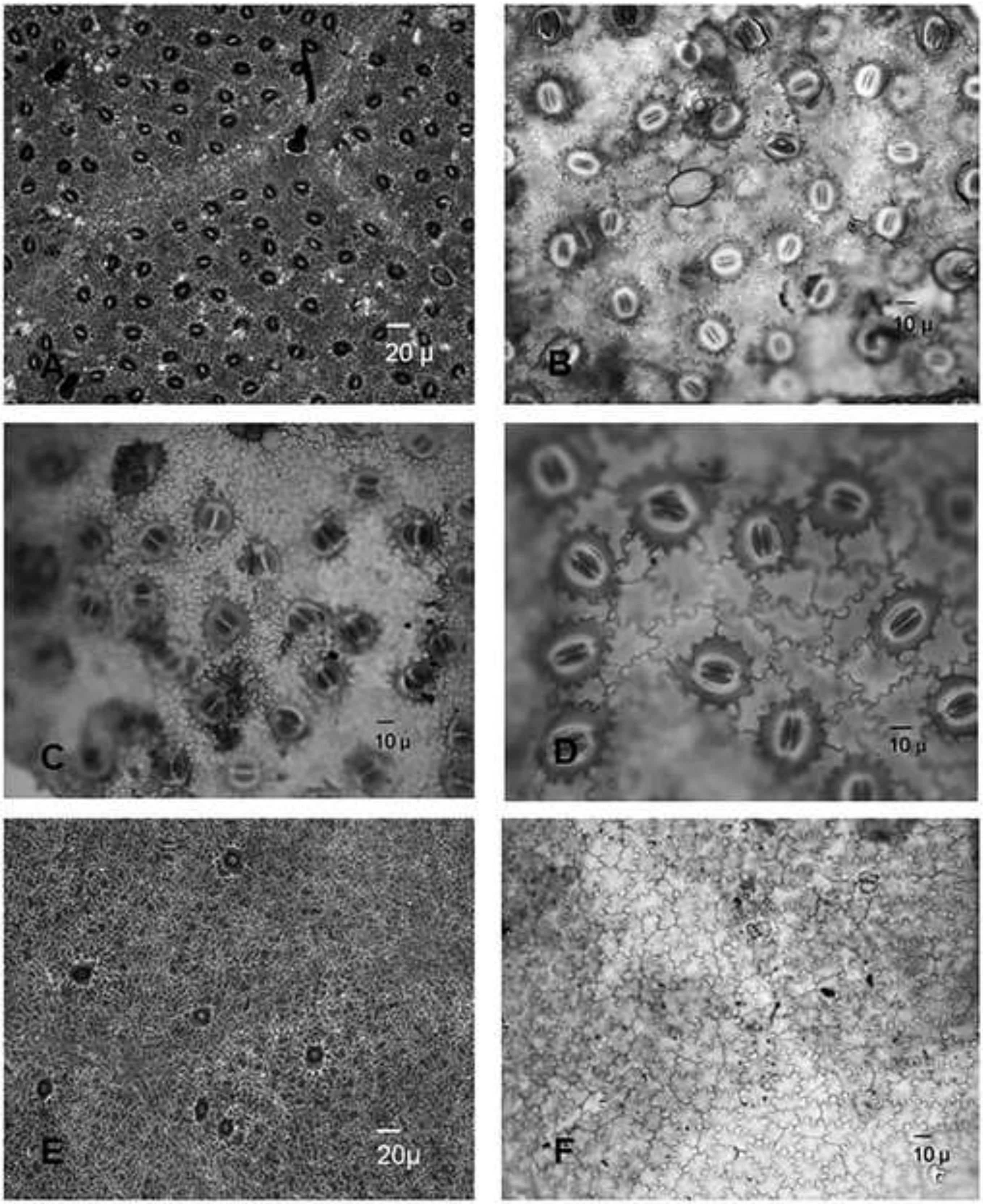

Fig 3

Click here to access/download;Figure;Fig 3.tif

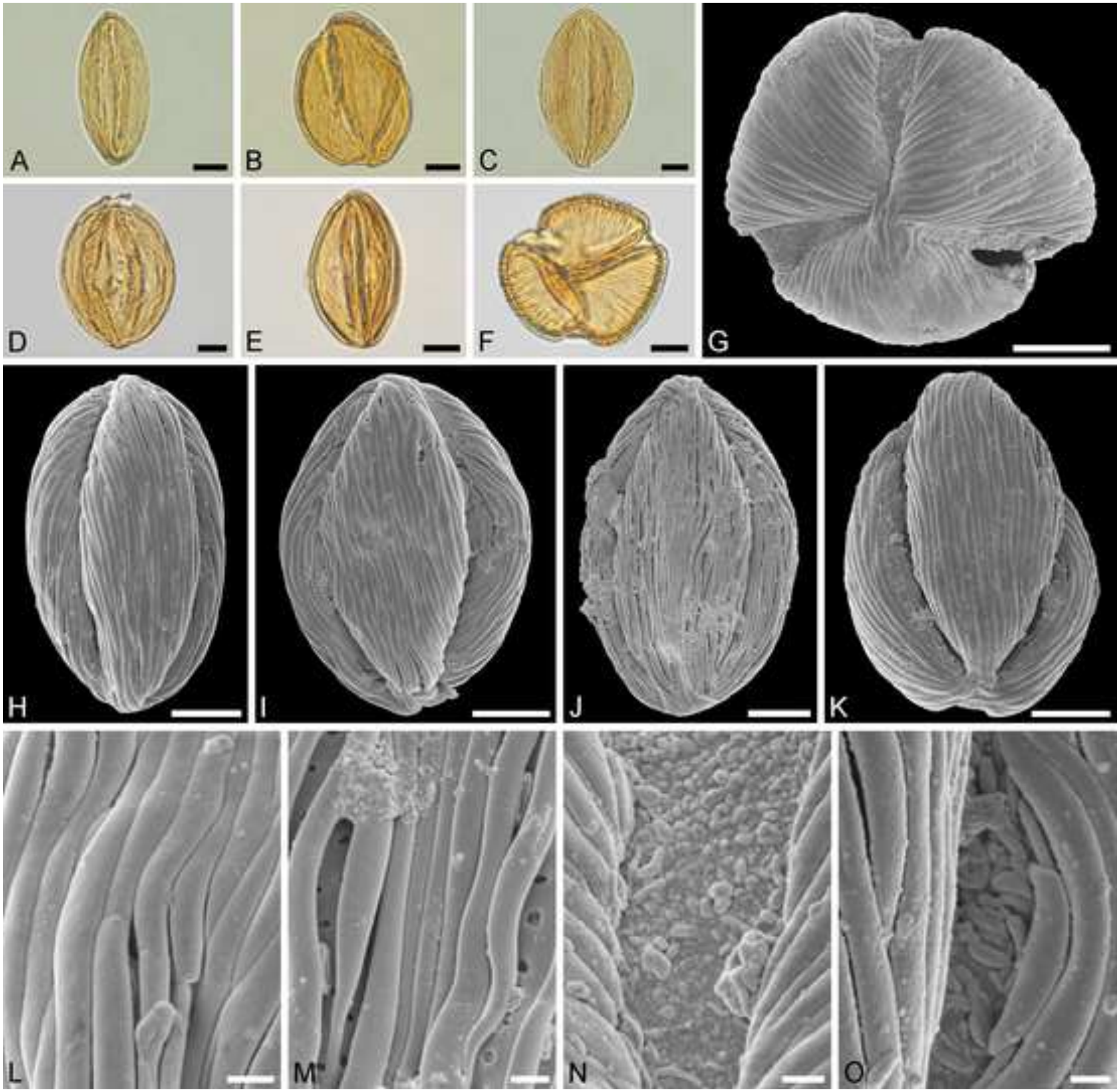

Fig 4

Click here to access/download;Figure;Fig 4.tif

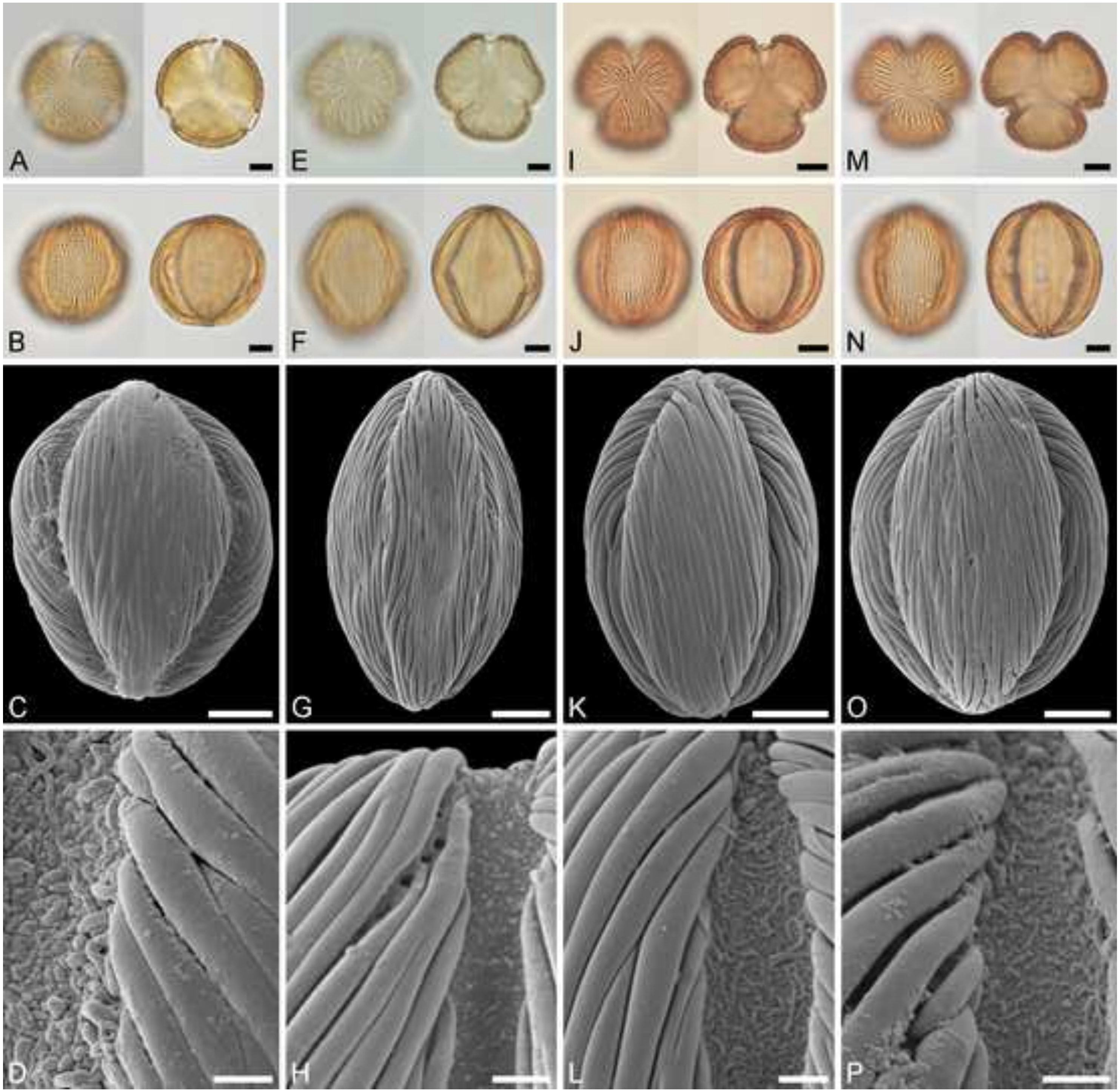

Fig 5

Click here to access/download;Figure;Fig 5.tif

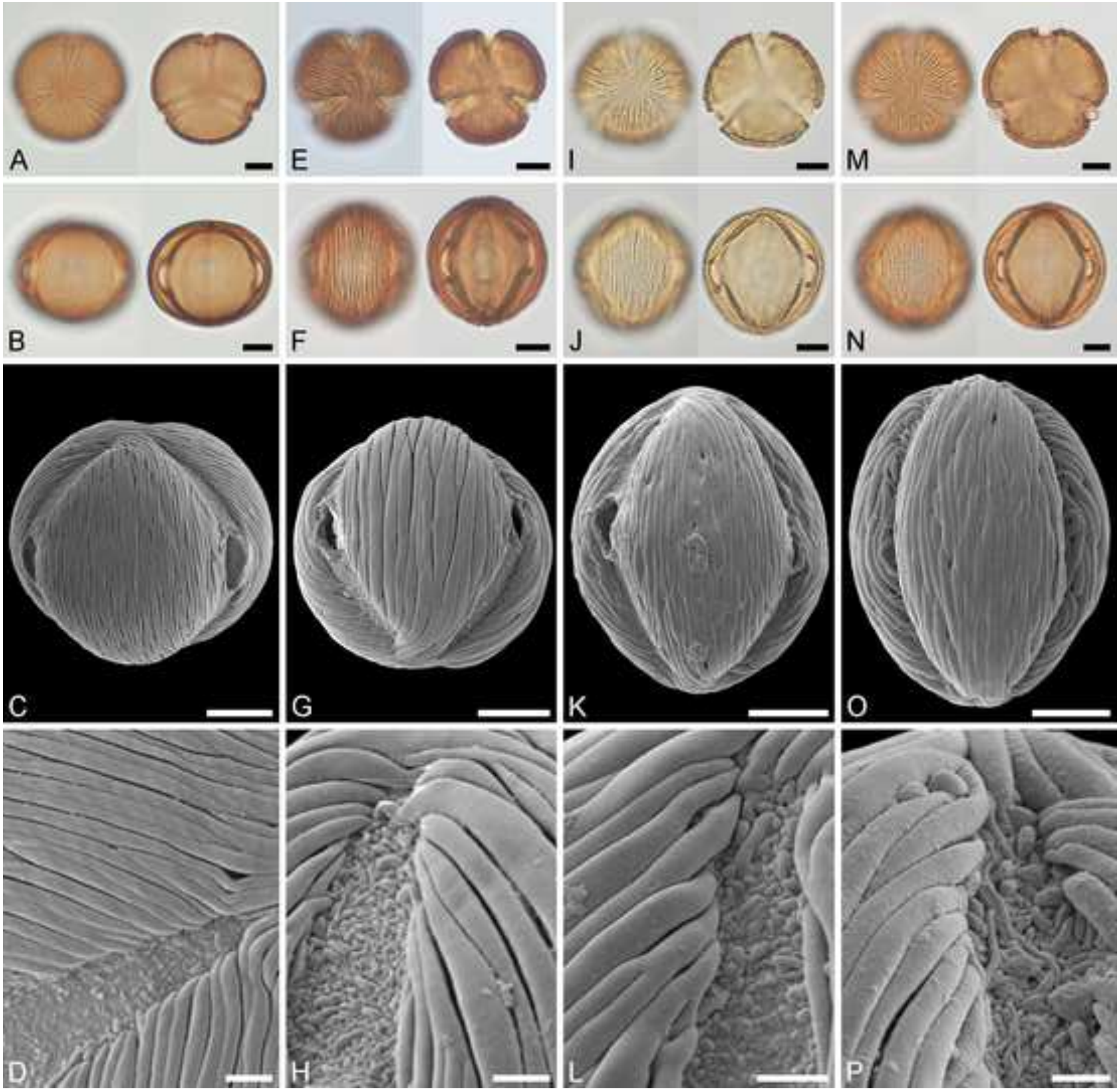

Fig 6

Click here to access/download;Figure;Fig 6.tif

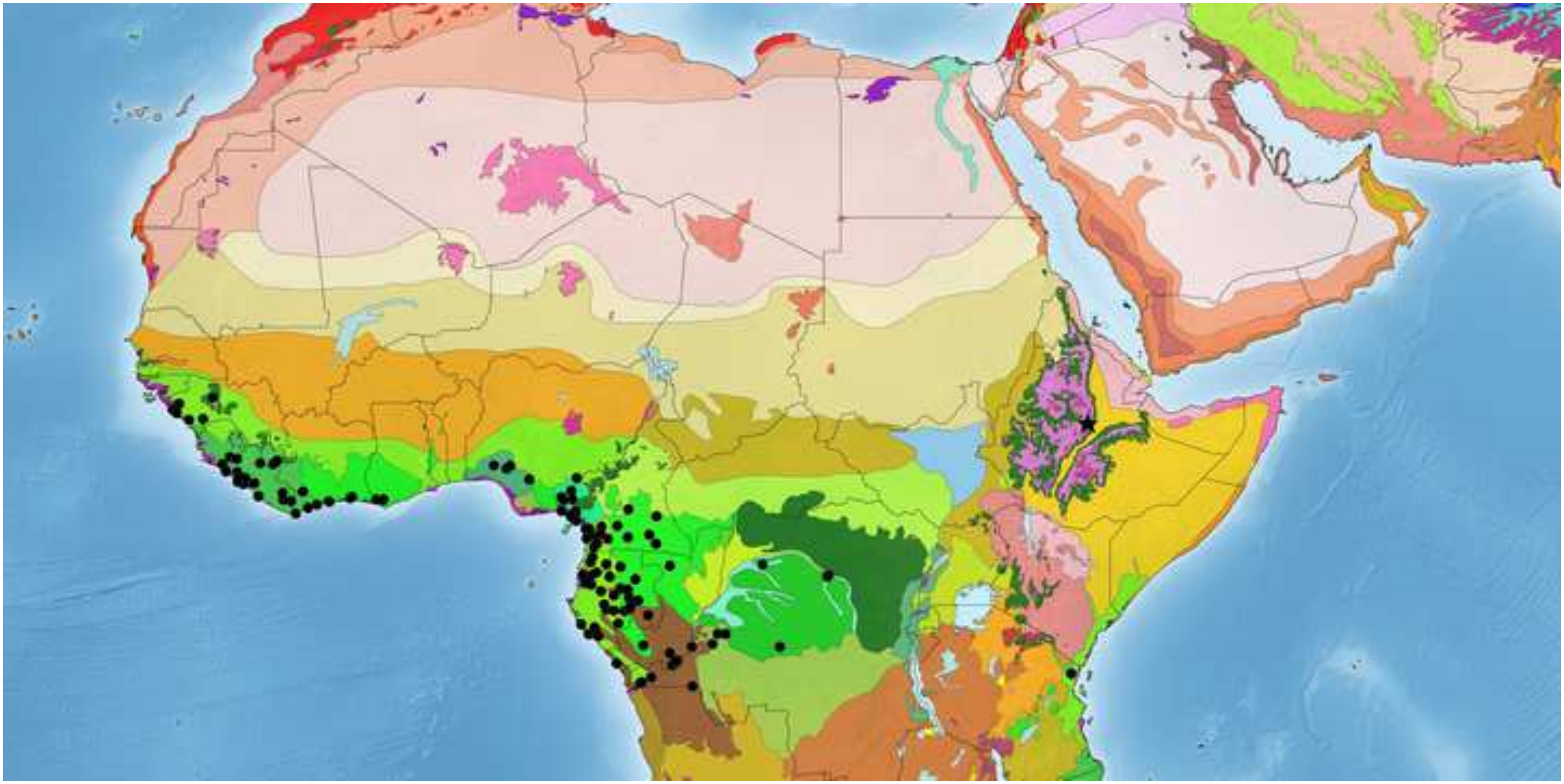

S1

Click here to access/download
Supporting Information
S1.xlsx

S2

Click here to access/download
Supporting Information
S2.xlsx

S3

Click here to access/download
Supporting Information
S3.xlsx

S4

Click here to access/download
Supporting Information
S4.pdf
