# Supplementary material for: First evidence of a monodominant (Englerodendron, Amherstieae, Detarioideae, Leguminosae) tropical moist forest from the early Miocene (21.73 Ma) of Ethiopia
Source: PLoS One. 2023 Jan 11;18(1):e0279491. doi: 10.1371/journal.pone.0279491 (PMC9833558; doi:10.1371/journal.pone.0279491)
Supplement: Attachment — Submitted filename: Rebuttal letter.docx [file pone.0279491.s009.docx]

November 5, 2022

Dr. Gongle Shi, Academic Editor, and Reviewer 1 and Reviewer 2:

Thank you very much for your review of our manuscript [PONE-D-22-26695]: First evidence of a monodominant (*Englerodendron*, Amherstieae, Detarioideae, Leguminosae) tropical moist forest from the early Miocene (21.73 Ma) of Ethiopia, on behalf of *PLOS ONE*. We appreciate the opportunity to submit the minor revision of our paper for your consideration. Below we respond to the editor’s and reviewers’ comments and note corresponding changes we have made in our paper.

We have reviewed our revised manuscript to make sure that it meets *PLOS ONE*’s style requirements. We have included a completed copy of PLOS’ questionnaire on inclusivity in global research with the revised manuscript and have included it in Supporting Information as S1. We have included our full ethics statement in the Methods section.

*PLOS ONE* and the academic editor had some concern about Figure 6, now Figure 7 in the revised manuscript, and that it might copyrighted. This figure was created solely using Simplemappr, which is in the public domain (similar to USGS National Map Viewer, The Gateway to Astronaut Photography of Earth, etc.) and which we have seen used in other article published by *PLOS ONE*. The following language is included on the website: “All versions of SimpleMappr map data found on this website are in the Public Domain. You may use the maps in any manner, including modifying the content and design, electronic dissemination, and offset printing. The primary author, David P. Shorthouse has waived all copyright, related or neighboring rights, and financial claim to the maps and invites you to use them for personal, educational, and commercial purposes. No permission is needed to use SimpleMappr. Crediting the author is unnecessary.” (<https://www.simplemappr.net/#tabs=6>). In the figure legend we have included the following language, “This map was created with Simplemappr (<https://www.simplemappr.net/)>.”

We have included the language required for new species in a “Nomenclature” sub-section in the Methods section as required by *PLOS ONE*. In addition, we have changed all ‘Fabaceae’ to ‘Leguminosae’ for consistency. In addition, small grammatical or punctuation errors that we have found in the course of this revision have been corrected.

We have reviewed the reference section and have made no changes. We have updated some information in the Supporting Information to provide additional images as requested by Reviewer 2.

Below you will find our responses to the comments and concerns of Reviewer 1 and Reviewer 2:

Reviewer 1 (R1) concern:

“The tiff files of figures 1 &2 I was able to download are not of the best resolution. I had difficulty seeing some characters mentioned in the diagnosis and detailed description. I would also like to see additional closeups of some of the leaflets (e.g., twisted pulvinus, secondary and higher order venation.) Since this is the flora’s most dominant taxon, there should be plenty of images. It looks like this “Legume 1 – Englerodendron mulugeta sp. nov.” has been informally described/figured in other articles. However, the reader of this paper should see all the relevant images given that a new species is being erected.”

Authors’ Response:

We have redone Fig 1 and have made an additional figure (which is now Fig 2) so that the characters in the diagnosis and description can be more easily seen. This includes high resolution and larger images of the fossil species, as well as extant species. We have specifically added an image of MU29-41 #4 (Figure 2C), which has very good details of the canaliculate midvein, and the secondary, tertiary, and quaternary venation. 5^th^ level venation of the taxon can be observed in Figure 3F of Bush et al. (2017). In addition, in the Supporting information we have provided images of 4 more specimens that provide additional viewing of the characteristics noted in the manuscript.

Reviewer 1 (R1) concern:

“The images of the fossil cuticles of the new species appear blurry in the Tiff file. Please consider new photos of the fossil cuticles at 10 μm to facilitate the comparisons with living taxa.”

Authors’ Response:

We have selected new images of the fossil cuticle and have made sure that the resolution is at 300 dpi.

Reviewer 1 (R1) concern:

“I have a few concerns regarding the monodominance of the forest, and I like that the authors stated, “it is possible that the forest at the time of Level A was on its ways to monodominance”. Although the percentages of the fossil leaflets are relatively large, they don’t appear to be entirely the dominant taxon when compared with the few data available from tropical Africa (Table 3, Peh mono 1-3). Considering that the leaflets were counted as leaves needs additional treatment in the statistical analyses.”

Authors’ Response:

The dominance of the fossil *Englerodendron* in the Mush plant assemblages is particularly notable, not only in terms of the abundance of the morphotype/taxon in Levels B - F, and prevalence in Level A, but also in representing the most abundant taxon at around 5 – 6.5 times (excluding Level A, which is 3 times) the level of the next most abundant species over a duration of tens of thousands of years. The taxon does not appear to be a short-term proliferating species that has been sampled in a single large stratum in abundance, but in multiple levels, likely showing an estimated 40,000 – 50,000 years of dominance. In regards to comparisons with Peh et al.’s (2012) study, we believe that it is valid. The dominant species in his work is *Gilbertiodendron dewevrei*, which is also a paripinnate-compound leaved legume species in the Berlinia Clade. *Gilbertiodendron dewevrei*, is typically 3-jugate, so each leaf usually consists of 6 leaflets. In the leaf litter fall studies whole and partial leaves (including leaflets) are used in litterfall weighing. In addition, the new fossil *Englerodendron* species is not the only compound-leaved legume in the flora, with the next two taxa (‘Short-drip tip’ and ‘Interrupt’) each making up 4 – 10% of the material in each level, but never to the extent of the fossil *Englerodendron* species. Conversely, other taxa present in the fossil flora, *Newtonia mushensis* (Pan et al. 2012) and *Zanthoxylum* sp., are only represented as fruits and seeds. Both genera, *Newtonia* (Leguminosae: Mimoseae) and *Zanthoxylum* (Rutaceae), are noted for possessing diagnostic leaves (with diagnostic leaflets) that are pinnately-compound. No potential candidates representing either of these genera have been found amongst the fossil leaf(let) material from Mush. We believe the statistical analyses in which we have used Hill numbers for Mush and Chilga (Guang and Bull’s Bellow) provide evidence for the presence of monodominance of *Englerodendron* at Mush. We also note that the dominant taxa at Chilga are *Cynometra chaka* (Guang) and an *Albizia*-like legume leaf type (Bull's Bellow), both of which have compound leaves.

Reviewer 1 (R1) concern:

“It would be nice if the coauthors added a new figure plotting “Percent Dominant Taxon” against the stratigraphic levels.”

Authors’ Response:

We have added a bar graph figure, Fig 8, which shows percent of abundance of the fossil *Englerodendron* leaflets plotted against stratigraphic height. In addition, similar bar plots for abundances of the taxa can be found in Currano et al. (2020) as Figure 6.

In addition, we have made almost all of the recommended changes that Reviewer 1 (R1) noted in the manuscript. We have kept the description of some of the petioles as ‘terete’ instead of ‘interpreted or likely terete’ as suggested by R1, because some of the fossils, particularly as seen in Fig 2A and S5, show straight pulvinate petioles indicating the presence of this characteristic, along with some specimens possessing twisted pulvinate petiolules in the fossil *Englerodendron* species.

Reviewer 2 (R2) concern:

“1. Please revise the international code of botanical nomenclature for correct epithet assignation. Is ‘mulugeta’ treated as a latinized noun in apposition? The reference that the authors provide for this epithet is “he who rules”, which sounds more like an adjective instead. If this is the case (adjective), the adequate spelling should be “mulugetum”. If naming in honor of Dr. Mulugeta Fesesha it should be “mulugetanum”.”

Authors’ Response:

We fully agree and thank R2 for informing us on this issue. We have changed the proposed species name to *Englerodendron mulugetanum* and have changed the etymology section accordingly.

Reviewer 2 (R2) concern:

“2. Figure 1 – The quality of images is quite poor and therefore the species description and morphological comparisons are hard to follow. The little detail that was visible is consistent with the species description, but I suggest that the authors include better images and/or consider including an illustration of the type specimen. Particularly the fine venation is impossible to figure out.”

Authors’ Response:

This is similar to the concerns of R1 and we have changed the images accordingly to make sure that we have provided images to assist the reader in recognizing and viewing the characteristics of the new species. We have redone Fig 1 and added another figure (now Fig 2) so that the characters in the diagnosis and description can be determined more easily, including high resolution images of the fossil and extant species. Specimen MU29-41 #4 (Figure 2C) has been added and provides very good details of the canaliculate midvein, and the secondary, tertiary, and quaternary venation. 5th level venation of the taxon can be observed in Figure 3F of Bush et al. (2017). We have also provided images of 4 additional specimens in Supporting information.

Reviewer 2 (R2) concern:

“3. Even though the authors include a Table with detailed morphological comparison between the fossils and living species of Detarioideae, I encourage the authors to include a supplementary figure with leaves of the species they compared the fossils with, as a means to provide stronger evidence for their taxonomic assessment.”

Authors’ Response:

We have created an additional document in Supporting Information that includes a number of examples of other Amherstieae leaf(let) images and characteristics to assist the reader. This was a very good idea. Thank you!

Again, we want to thank the academic editor and the 2 reviewers for very helpful suggestions to assist in making this a better paper. If you have any additional questions or need more information, please let us know. Thank you again for consideration of our revised manuscript.

Kind regards,

Aaron D. Pan, Ph.D.

Executive Director

Museum of Texas Tech University

Texas Tech University
